# Toward Better Reproducibility in Experimental Research on New Agents for Pulmonary Hypertension. An Analysis of Data from Four Hundred Animal Studies

**DOI:** 10.1007/s10557-020-07109-3

**Published:** 2020-12-09

**Authors:** Magdalena Jasińska-Stroschein

**Affiliations:** Department of Biopharmacy, Medical University of Łódź, ul. Muszyńskiego 1, 90-151 Lodz, Poland

**Keywords:** Experimental, Animal model, Pulmonary hypertension, New agents, Bias

## Abstract

**Purpose:**

Pre-clinical data can provide a rationale for subsequent clinical trials and they are the first step in drug development; however, the therapeutic effect observed during animal studies does not necessarily translate to similar results in humans.

**Methods:**

Taking the example of pulmonary hypertension, the present study explores whether the methodological aspects of preclinical experiments can determine the final result.

**Results:**

The present paper describes a systematic analysis of 409 studies conducted on a variety of animal models to identify potential drug candidates for PH treatment; it explores the influence of various aspects of study design on the final outcome, e.g. type of animal model of PH, dosage schedules of tested agents, type of anesthesia, measurement of exercise intolerance or animal survival.

**Conclusions:**

The animal models of PH used for pre-clinical studies are diverse and there are several methodological items within the established protocols that can determine the obtained result.

**Figure:**
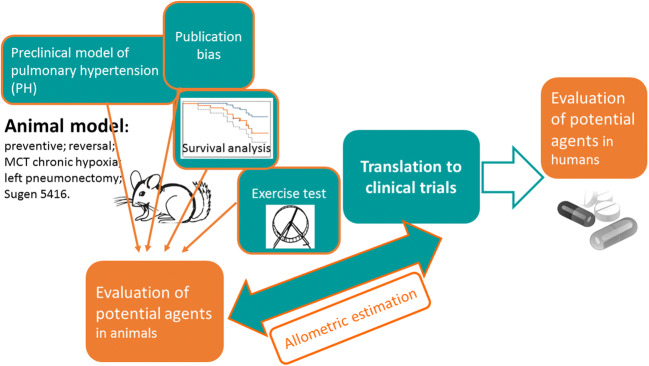
Graphical abstract

**Supplementary Information:**

The online version contains supplementary material available at 10.1007/s10557-020-07109-3.

## Introduction

As the first stage in the process, preclinical studies play a crucial role in drug development, as they designate potential candidates for further clinical assessments. Far, beyond predicting clinical effects, they can also clarify the appropriate application of a drug, dosage or indication. Unfortunately, the success rate of drug submissions has been declining over the past 30 years, and it has been estimated that only 16% of drugs entering clinical development reach the pharmaceutical market [1]; the lowest rates have been observed for one cardiovascular agents, with only 7–8% being approved [2]. Over 50% of agents entering clinical trial fail due to lack of efficacy. Such losses are commonly observed in the first or second phase of clinical studies, with some therapeutic effect observed during preclinical studies conducted on animals failing to translate to similar results in humans. As discussed previously, such phenomena can be attributed to poor planning, conduct and reporting of most preclinical studies [3]. Hence, translation from animal to human models is not precise, and this difference can affect preclinical studies. According to previous reports, only 50% of preclinical experiments are reproducible and errors involving biological reagents and reference materials, study design, data analysis and reporting or laboratory protocols can contribute to such irreproducibility [4].

Pulmonary hypertension (PH) is a progressive and incurable disease with a multifactorial etiology and poor prognosis characterized by excessive pressure in the pulmonary vasculature; however, when affecting the small pulmonary arterioles, it is classified as pulmonary arterial hypertension (PAH). Treatment options have improved significantly in the last 20 years, but for many PAH patients, disease management often fails due to heart failure. There is therefore a constant need to develop novel therapies. As a result, to explore pathomechanism of the disease and to identify potential effective substances for further clinical evaluations, a considerable number of preclinical observations, including experiments on animal models, have been performed. However, although a number of tested substances have demonstrated a range of potential therapeutic benefits in these early stages, many fail to demonstrate clinical benefit in further studies involving patients with PH. Some notable disappointments in the introduction of novel agents for this disease include terguride, 5-HT2A and 5-HT2B antagonist, HMG-CoA reductase inhibitors (statins), aviptadil, vasoactive intestinal polypeptide (VIP) [5], or a selective, orally available (apoptosis signal-regulating kinase 1) ASK1 inhibitor [6].

The failure in clinical translation is in some part associated with the limitations associated with the usage of animal models; as PH typically describes a highly heterogeneous and complex clinical manifestation, no one single model is considered a perfect substitute. Several rodent models are available to study PH. Unfortunately, none have been classified according to a similar taxonomy as the WHO PH categorization, and they are unable to model the comprehensive hemodynamic and histological lesions in human PH [7]. In addition to the choice of particular animal model, testing of novel agents can also be influenced by a range of methodological aspects including the choice of strain, time course, allometric scaling and dosage, among others.

In line with these findings, the aim was to explore whether certain methodological and statistical aspects of preclinical experiments can determine the final result. The present survey examines a number of methodological items within study design; however, some statistical aspects such as power, data dispersion, sample size determination, sufficient number of animals, and etc. will be discussed in the separate paper. No attempt is made to compare the efficacy of particular drug candidates toward PH: such detailed analyses have been provided in recent papers [8].

The obtained results will help add rigor and improve the quality of preclinical studies. The methodological considerations for preclinical studies were based on 409 studies performed from 1991 to 2018 to identify novel potential agents for pulmonary hypertension.

## Methods and Materials

The systematic review comprised Medline (1991–2018) and ISI Web of Science (1991–2018). The databases were searched with no language restrictions using the following search terms in titles and abstracts: (mice OR rat OR mammals) AND (pulmonary hypertension) AND (RVP OR PAP OR RVSP OR RVH OR wall thickness OR RV/LV + S OR pulmonary artery pressure OR remodeling).

### Inclusion and Exclusion Criteria

**Fig. 1:**
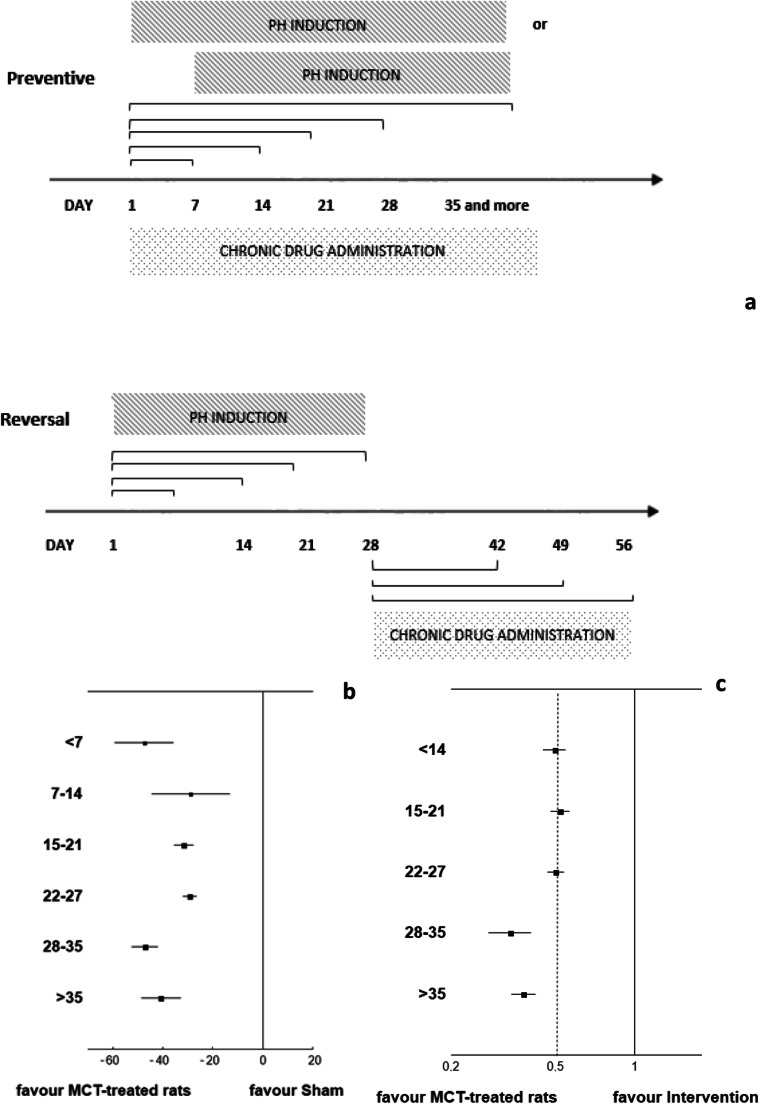
The influence of experimental period on the overall effect of candidate drugs for PH (*N = 250 studies*) on the example of the monocrotaline-based protocols. **a** The variety of possible schedules included in the meta-analysis. Preventive regimen – the administration of tested agents begins with or few days before induction of PH; reversal regimen – the PH induction is followed by a drug exposure period. For both regimens – induction and drug administration can last from seven to 35 days or more.  indicates the possible options for duration of particular procedures (PH induction and drug administration). **b** Tree plot for the effect size: mean difference for Vehicle (MCT-treated rats) indicates the relationship between duration of the induction procedure and overall result. **c** Tree plot for the effect size: response ratio for Intervention (MCT + tested agent) indicates the relationship between duration of the whole experiment and overall result. PH was induced by a single injection of monocrotaline, most commonly 60 mg/kg bw. In general, the animal performance worsened due to induction of PH as compared to the healthy rats (Sham) (b) (*P* < 0.0005) (effect size increased), while potential medication agents (Intervention group) normalized animal parameters (*P* < 0.0001) as compared to the MCT-treated rats (the effect size of 0.5 indicate that the examined agents reduced the mean value of the parameter by only half, while R = 1.0 would define one agent ability to completely reverse altered PH parameters) (c). A prolonged induction period was accompanied with increased effect size, i.e. pronounced development of PH-linked lesions, in Vehicle (Q = 47.07; df = 4; P < 0.0001) as compared to the Sham (b). In contrast, for longer experiments, a poorer response to potential medical agents was observed in the Intervention group (Q = 39.56; df = 4; P < 0.0001) as compared to the Vehicle (c). Such tendency reverses after 35 day, when the effect size is slightly reduced in the Vehicle, and augmented in the Intervention group

The results included experiments where animals had been exposed to several interventions to prevent or reverse disease with potential significant haemodynamic improvement in the pulmonary circulation. The outcome of an intervention in a cohort of animals was compared with that of a cohort of healthy animals (Sham) and an untreated group exposed to one or more procedures to introduce PH (Vehicle). The exclusion criteria were as follows: experiments on pregnant, newborn or genetically manipulated animals, studies in which acute PH was induced or several medications were examined after their acute administration, and studies that lacked data such as number of animals or dosage of the tested drugs. Also, papers that assessed pathophysiological aspects rather than drug efficacy were not included in the analysis. Detailed information is provided in Supplementary data (Fig. 1).

### Data Extraction

For the purposes of present survey, the following data were recorded: animal model of pulmonary hypertension (inductor, dose), anesthetic(s) used during haemodynamic measurements, name of tested substance to prevent or reverse PH (agent, dosage, time and route of administration), outcomes (alterations in right ventricle mean/systolic pressure – RVP/RVSP, in mean pulmonary artery pressure – mPAP, and/or right ventricle hypertrophy (RVH) – usually expressed as RV/LV + S ratio), and animal mortality. The outcome measure included mean, SD or SEM, and number of animals per group.

### Quantitative Data Synthesis

The analyses were conducted using STATISTICA 13.1 software. For each pairwise comparison between two treatments, the relative effect was calculated with a 95% confidence interval (CI). When the results of more than one comparison, i.e. tested agent vs. Vehicle, were reported in one study, e.g. due to different experimental conditions (e.g. dosage, route of administration), such a comparison was regarded as a separate intervention. In cases where the outcome measures were reported as median and range or 95% CI, mean and standard SD values were estimated according to Wan et al. (2014) [9]. Effect size for Vehicle group was assessed according to net changes in measurements:

1 $$ D=\overline{X_3}-\overline{X_1} $$

effect size for Intervention group was expressed as response ratio according to the following formula:

2 $$ R=\frac{{\bar{X}}_2-{\bar{X}}_1}{{\bar{X}}_3-{\bar{X}}_1}, $$

effect size for Intervention group for exercise studies was assessed according to net changes in measurements:

3 $$ D=\overline{X_2}-\overline{X_1} $$

where R – response ratio; ${\overline{X}}_1$– mean response in the Vehicle group (animals that were involved pulmonary hypertension; positive control); ${\overline{X}}_2$– mean response in the Intervention group (animals that underwent PH and were exposed to the tested agent); ${\overline{X}}_3$– mean response in the Sham group (negative control, healthy animals). The proposed formula (2) allows to minimize factors that are related to particular model of PH and assess properties of tested agents more independently [10].

Sub-group analyses and meta-regression [10] were performed to assess the impact of particular methodological items (type of study regimen, route of drug administration, dose of PH inductor, etc.) on the final outcome. A random-effects model was used to compensate for the heterogeneity of studies. Heterogeneity was quantitatively assessed using Cochran’s Q and I^2^ statistics.

### Other Analyses

The Kaplan-Meier method was used, and the significance of difference in survival according to animal group or established protocol was verified using the log-rank test.

Allometric estimations according to doses used in preclinical and clinical studies were performed using the following formula:

4 $$ HED\left( mg/ kg\right)= Animaldose\left( mg/ kg\right)\times \left(\frac{(AnimalKm)}{(HumanKm)}\right) $$

where HED is the human equivalent dose, Km factor express the body weight (kg) and body surface area (m^2^) ratio [11].

A two-tailed *P* value less than 0.05 was considered statistically significant.

### Publication Bias

Potential publication bias was examined using a visual inspection of Begg’s funnel plot asymmetry, Begg’s rank correlation, and Egger’s weighted regression. Duval and Tweedie ‘trim and fill’ was used to adjust the analysis for the effects of publication bias.

## Results

The section provides an overview of the influence of the study design on the final result, in particular, the selection of the animal model of the disease, major outcomes and type of anesthesia, animal survival, the additional outcomes, e.g. exercise capacity, and dosage regimens of the tested agents (i.e. dose and route of administration) with allometric estimations. The last section demonstrates publication bias.

### General Design

The animal model of the disease: in the majority (92.3%) of searched experiments, the most common intervention was a single pathological insult used with a single monocrotaline injection (58.5%) at a dose of 60 mg/kg bw or more (48.2%). The researchers most commonly explored the potential efficacy toward pulmonary arterial hypertension (51.9%) or pulmonary hypertension due to lung diseases and/or hypoxia (24.4%). Major outcomes included hemodynamic improvements, i.e. pulmonary artery pressure (PAP) and right ventricle (systolic) pressure (RV(S)P) (88.2%), In addition, assessments were made of myocardial hypertrophy (79.8%). Qualitative or quantitative histopathological assessments were performed in 43.2% of the studies. In more than half of the protocols (27.87%), the authors stated about blinding during histopathological assessments. Interestingly, the drug-induced PH prevention or reversal was significantly less pronounced (*P* = 0.0029) where such additional analyses were performed as compared to the protocols that did not combine hemodynamic and histopathological measurements. The assessments of exercise intolerance were performed in fewer than 2% of the reviewed studies. In preventive experiments (71.8%), the reagents were given before the induction of pulmonary hypertension, or when it was initiated. For reversal regimens, the drug candidates were usually administered to reverse the course of pulmonary hypertension 14–21 days after the beginning of the induction period. The median duration of administration of candidate drugs was 21 days (14–28). The median number of days for the whole experiment was 28 (21–28) (Supplementary Data Table 1 and Table 2). Figure 1a presents the variety of possible schedules that were applied in the reviewed studies according to therapeutic regimen (preventive or reversal) and periods for PH induction.

The efficacy of tested agents was assessed according to formula (2). The values of R = 0.5 indicate that one agent was able to reduce by half the mean value of particular parameter changed due to application of corresponding method of PH induction, while R = 1.0 would define one agent ability to completely reverse altered PH parameters. This formula allows different activities of the tested agent to be compared, e.g. its vasodilatory or anti-proliferative efficacy; it also allows the agent to be examined with regard to tissue target, e.g. pulmonary arteries or right ventricle, and for inter-studies heterogeneity to be reduced. The overall efficacy (size) of candidate interventions, expressed as R ratio, was R = 0.51 (95% CI, 0.48–0.54; *P* < 0.00001). Ninety-three percent of experiments were performed in SPI models, where PH developed under chronic hypoxia or from single monocrotaline injection. When the “one-hit” studies were matched to the “second hit” protocols – according to duration of the experiment (median number of days – 35), a different responses to the potential agents for PH were observed (*P* = 0.0043) (Table 2 Supplementary data). The observed efficacy was found to be slightly determined by a dose of PH inductor (Supplementary data, Fig. 2) and significantly determined by the duration of experiment (Fig. 1b and c). Prolonged induction caused pronounced development of PH-linked lesions (*P* < 0.0001). It was accompanied with poorer response to potential medical agents in intervention groups (P < 0.0001). A detailed comparison of the efficacy of example therapeutic groups with regard to the drug administration period is given in Fig. 3 (Supplementary data).

More than 20 different anesthesia schedules, including both monotherapy or combination, were used for the surgical procedures. Of the five most popular anesthetics used in the analyzed studies, i.e. isoflurane, urethane, pentobarbital sodium, xylazine plus ketamine and chloral hydrate, the anesthetic agent did not determine the overall effect. However, the animals examined under anesthesia by chloral hydrate demonstrated the strongest response to agents that normalized PH (effect size expressed as response ratio: R = 0.59; 95% CI, 0.48–0.72) (Supplementary data, Fig. 4).

### Animal Survival

**Fig. 2:**
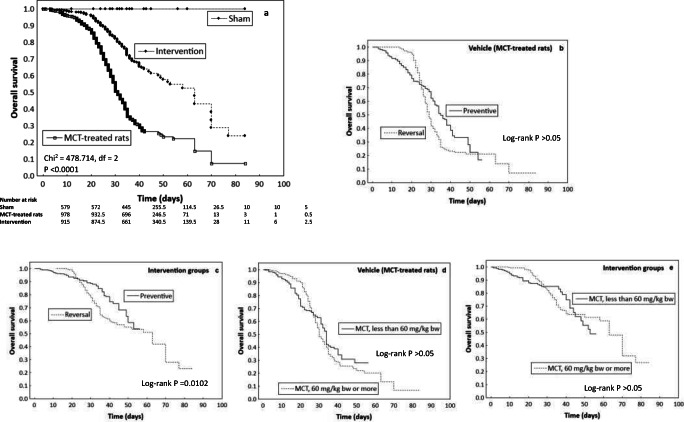
Kaplan-Meier survival curve of the overall survival of animals that were treated with candidate drug therapies for pulmonary hypertension (*N = 39 studies*). **a** The differences in animal survival among Intervention group, Vehicle (MCT-treated rats) and Sham (healthy rats) (*n* = 2472 animals). **b**, **c**, **d**, **e** subgroup analyses according to protocol (preventive vs reversal) and dose of PH inductor. b and d – Vehicle (MCT-treated rats, *n* = 978); c and e – animals receiving MCT + several candidate drug therapies (Intervention groups) (*n* = 934). The Intervention group demonstrated significantly greater overall survival than the Vehicle group (P < 0.0001). Tick marks indicate individuals whose survival times have been censored (a). The impact of protocol regimen (preventive vs. reversal) on overall survival was insufficient when only Vehicle animals were considered (*P* > 0.05) (b). The animal survival was significantly increased when the agents were administered for prevention of PH-mediated lesions as compared to the reversal protocol (*P* = 0.0102). The gap in the curve for reversal protocol results from delay in medication administration that started approximately 7–14 days after PH induction (c). Higher doses of monocrotaline, i.e. 60 mg/kg bw or more, did not significantly worsen the survival of animals from Vehicle or Intervention groups compared to lower doses (P > 0.05) (d – e). Survival curves indicate that preventive protocols and those in which PH induction was carried out using MCT at 50 mg/kg bw or less tended to use a shorter experiment period (b – e)

**Fig. 3:**
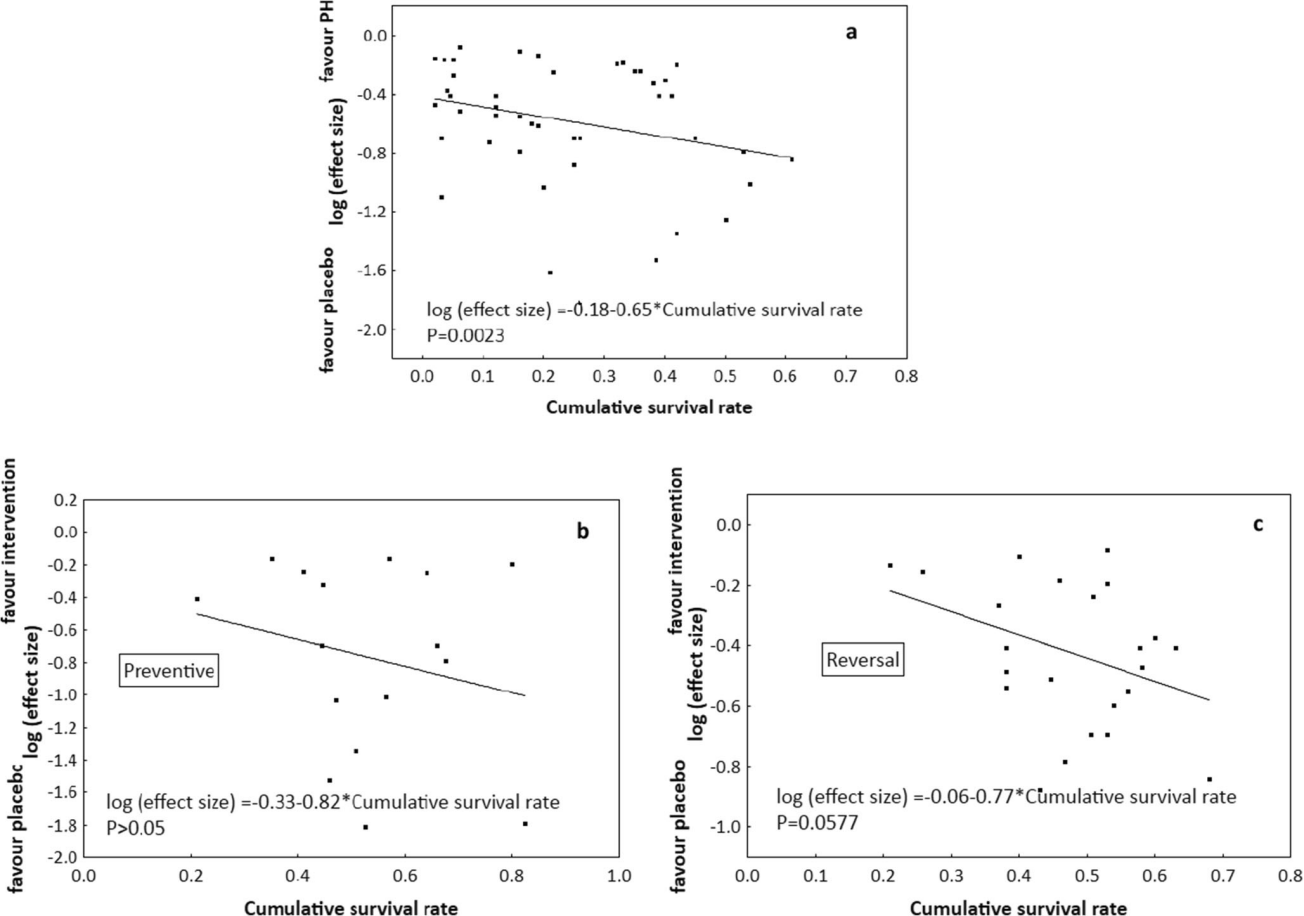
Bubble plot with fitted meta-regression line of the effect size (log) according to cumulative survival rate. Events were assumed to occur independently of one another, and the probabilities of surviving from one interval to the next were multiplied together to give the cumulative survival probability (*N = 39 studies*). **a** Vehicle group: together with an increased cumulative survival rate, the animals developed weaker model of pulmonary hypertension (*P* = 0.0023). Animals from Intervention groups were considered separately in subgroup analyses: **b** preventive protocol and **c** reversal protocol. The cumulative survival rate slightly decreased as the response improved to medical agents given to reverse PH (*P* = 0.0577) (b); this trend was not observed for drugs given to prevent pulmonary hypertension (*P* = 0.3937) (c)

Data about animal mortality during the experiment were provided in 76 out of 409 studies (18.6%), and Kaplan-Meier plots were given in 39 studies. This information concerned animal performance due to exposure to placebo (Sham), induction of pulmonary hypertension (Vehicle) and a number of tested agents (Intervention) that were given for the prevention or reversal of PH-linked lesions. Overall survival was found to significantly increase in the Intervention group compared to the Vehicle (*P* < 0.0001). Animal survival was determined by the study protocol; reversal protocols were linked to significantly greater animal mortality than those where potential medical agents were administered for the prevention of PH-mediated lesions (*P* = 0.0102). The majority of data about survival was provided by experiments where pulmonary hypertension was induced by monocrotaline injection; however, no significant discrepancies in animal mortality between higher and lower doses of monocrotaline were shown for the Vehicle or Intervention groups (Fig. 2). Figure 3 presents the relationship between cumulative survival rate and effect size; the strength of PH induction in the Vehicle group and the response to tested agents in the Intervention group. Together with the increased cumulative survival rate, the animals in the Vehicle group developed weaker model-induced pulmonary hypertension (decrease in effect size) (*P* = 0.0023), and subjects in the Intervention group responded slightly worse (*P* = 0.0577, reversal protocol) to potential agents for PH.

### Exercise Test

**Fig. 4:**
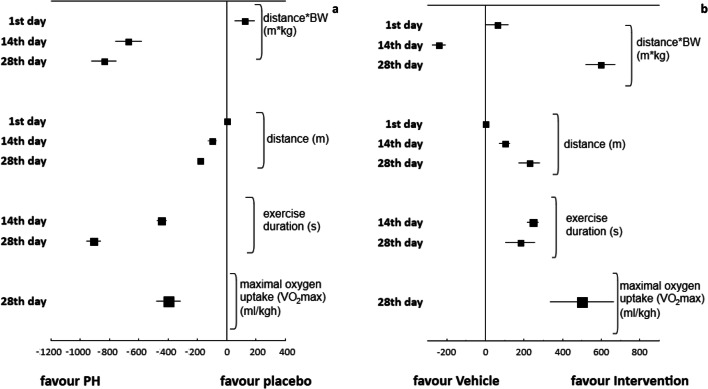
Studies on animal exercise capacity (*N = 8 studies*). Tree plot for the effect size for the Vehicle (**a**) and several medical agents (**b**) in accordance with the variety of protocols to assess exercise capacity. The induction of PH reduced the animal capacity (a) (*P* < 0.0001) as compared to the Sham (healthy rats), while potential medication agents (Intervention) normalized exercise tolerance (P < 0.0001) (b). Significantly different results were obtained from the exercise test depending on the measured parameter (distance in meters, exercise duration in seconds, etc.) (a – Q = 17.55; df = 3, *P* = 0.0005; b – Q = 15.34; df = 3; *P* = 0.0015) as well as time point (1st, 14th or 28th day of the study) (a – Q = 14.20; df = 2; *P* = 0.0008; b – Q = 10.32; df = 2; *P* = 0.0057) for both comparisons: Vehicle vs. Sham (a) and Intervention vs. Vehicle (b)

Exercise tests were performed in eight of the 409 studies included in the meta-analysis. A variety of protocols were established (Supplementary data Table 3). Several methods were used, such as a motor-driven treadmill or swimming pool. The exercise tests were performed at least once prior to induction of pulmonary hypertension, and again at the end of the experiment, on the animals randomized to Sham (negative control), Vehicle (positive control) and Intervention group. The final result of the exercise test was expressed as treadmill distance (m), treadmill distance multiplied by body weight (mkg), exercise duration (min or sec) or maximal oxygen uptake parameter (ml/kgh). Induction of pulmonary hypertension reduced exercise capacity, and the potential medication agents significantly decreased exercise intolerance. The results of the exercise test varied significantly with regard to the chosen method (*P* < 0.005). The exercise endurance of vehicle animals tended to decrease as the period of PH induction lengthened (*P* = 0.0008), while extended exposure to tested medical agents normalized exercise capacity (*P* = 0.0057) (Fig. 4).

### Dosage Regimens

**Fig. 5:**
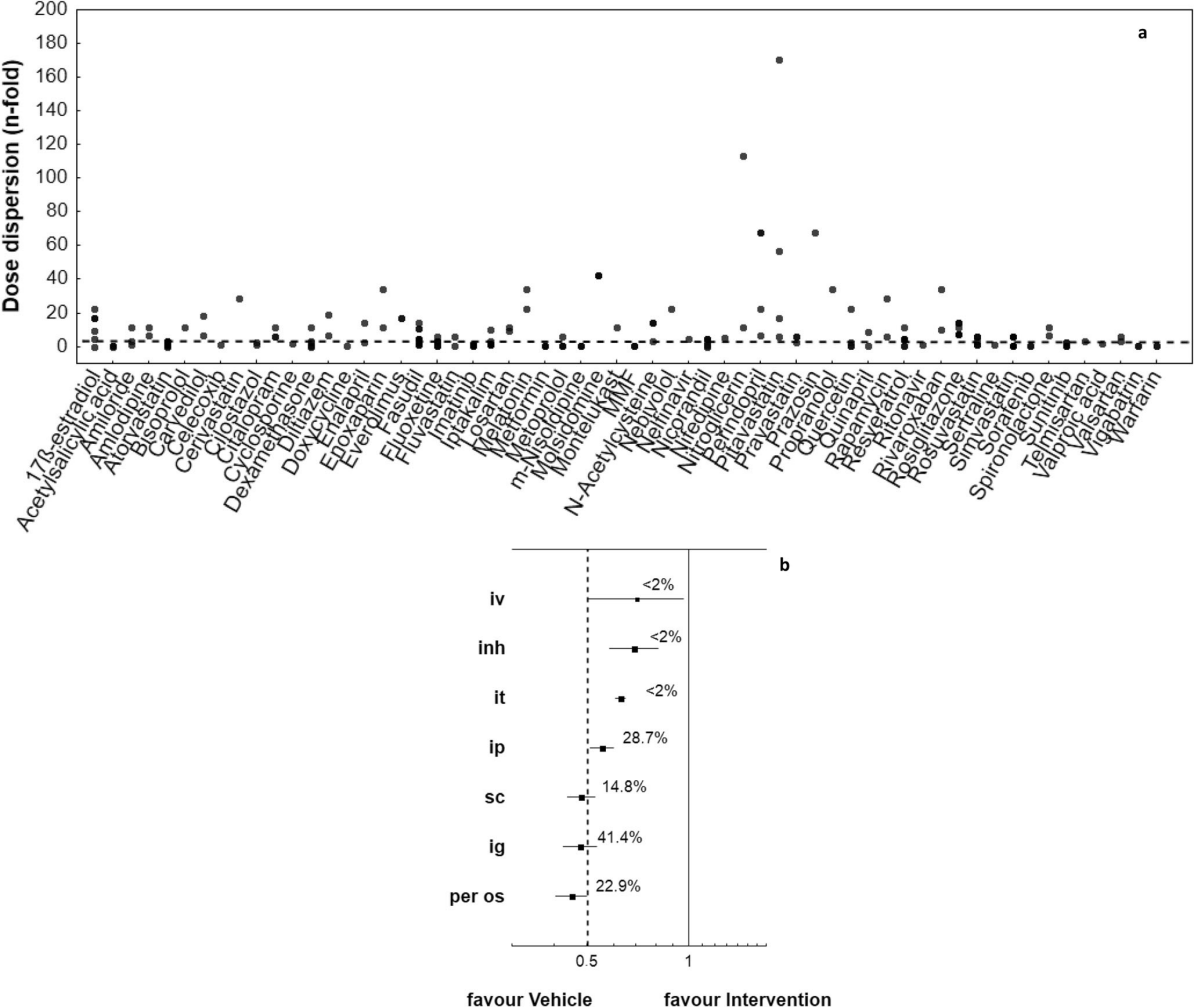
The dosage of candidate drug administration and overall benefit of treatment. **a** Dispersion of doses of example agents (•) that were used in the experiments with regard to the human doses (dashed line -----) (*N = 155 studies*). The *n-fold change* is the ratio of the particular animal dose converted according to allometric scaling (Eq. 5) and mean human dose. **b** Tree plot for the effect size (R) indicates the significant relationship between the route of the tested agent administration and final effect size (P < 0.0001; Q = 98.13; df = 7). Oral administration reduced the mean value of a particular parameter by less than half (response ratio, R < 0.5); this means the relatively weakest effect regarding the reduction or prevention of PH-related changes (*N = 409 studies*)

The doses used for the agents demonstrated wide dispersion, with the relative standard deviation of diltiazem, rivaroxaban, terguride, imatinib, resveratrol, nicorandil, simvastatin or rosiglitazone exceeding 100% (Supplementary data Table 4). Figure 5a presents the dispersion of the applied doses of example agents tested for PH in relation to those used for humans. The more pronounced discrepancies concerned HMG-CoA reductase inhibitors (statins), RAAS inhibitors (e.g., losartan, carvedilol), calcium channel blockers (e.g., diltiazem, nilsodipine), or RhoA/ROCK inhibitor (fasudil), rosiglitazone, MMF or everolimus.

The route of drug administration was found to significantly influence effect size (*P* < 0.0001). The predominant route of administration was intragastric (41% of analyzed interventions). In approximately 20% of cases the tested agents were given in drink water, resulting in the relatively weakest effect regarding the reduction or prevention of PH-related changes. In another 30% of interventions, the drugs were injected intraperitonealy, resulting in a response ratio exceeding 0.5 (Fig. 5b and Supplementary data Fig. 5).

### Publication Bias

The results of analyses of publication bias are demonstrated in Supplementary Data (Fig. 6) with subgroup of animals, e.g. subjects that were exposed to monocrotaline or these for which survival data or exercise test were reported. In majority the funnel plot of study standard error by random effect shows asymmetry.

## Discussion

As rodent models are commonly used to understand human pathophysiology and for preclinical assessment of experimental therapies, they should reflect the particular disease with regard to its histology, molecular biology or therapeutic response. Such models have also provided key insights into the pathogenesis of pulmonary hypertension. Although they do not mimic the complexity of pathophysiological lesions of this disease, they still play a dominant role in preclinical evaluations for PH therapies. Studies with rodents can be performed using SPI (“one-hit”) models, where PH is induced by a single pathological insult (e.g., monocrotaline – MCT, chronic hypoxia), MPI (“second-hit”) models caused by multiple-pathological insult (e.g., MCT + pneumonectomy, MCT + chronic hypoxia, chronic hypoxia + Sugen 5416), knockout models (e.g., morphogenetic protein receptor-2 – BMPR-2, vasoactive intestinal peptide) and overexpression models (e.g., interleukin-6, angiopoeitin-1).

Due to diverse and multifactorial etiology and mechanistic background of the disease, rodent models should be classified by a similar taxonomy as for humans. The majority of SPI and MPI models are indicated as being suitable for pulmonary arterial hypertension, with none being suitable for WHO 1′ (patients with pulmonary venoocclusive disease and pulmonary capillary haemangiomatosis), group 2 (PH is secondary to elevation of left atrial pressures due to left heart disease) or group 4 (secondary to chronic thromboembolic disease) [12]. Although chronic hypoxia is a model of group 3 (pulmonary hypertension owing to lung diseases and/or hypoxia), it can be incorrectly extrapolated to represent those of group 1. Hypoxic rats develop medial hypertrophy of pulmonary arteries with minimal evidence of vascular obstruction and no intimal fibrosis or plexiform lesions [7]. Conversely, approximately one quarter of the reviewed experimental therapies were intended for group 1 – arterial pulmonary hypertension, but were tested only in chronically hypoxic rats.

Furthermore, more than 90 % of experiments were performed in SPI models, where PH developed under chronic hypoxia or from single monocrotaline injection. As previously explored, such models do not completely display the severity of PH observed in humans with respect to the histological and/or hemodynamic parameters. Some authors claim that SPI may very well correlate with milder forms of human PH, a stage that is often missed at the time of diagnosis [13]. Ryan et al. (2011) [7] conclude that in experiments based on the single animal model of chronic hypoxia, most tested agents were revealed to be ‘effective’ in regressing PH because this model is not reflective of the challenges that exist in improving the vascular bed, haemodynamic status or survival in humans. Similarly, previous survey found that PH-linked lesions tended to be more developed when the experiments were performed on an animal model of monocrotaline than chronic hypoxia [14]. At the same time, a review of the efficacy of 522 interventions with more than 200 unregistered drugs found only 41 to be ineffective [8]. One of the most promising rodent models of increased pulmonary flow is created by unilateral left pneumonectomy combined with a “second hit” of MCT or Sugen 5416 [15, 16]. Such a combination produces severe PH by increased turbulent pulmonary blood flow and vascular remodeling with subsequent endothelial dysfunction. A few reviewed protocols included MPI approaches, and it was impossible to perform head-to-head comparisons between one-hit” and “two-hit” model. However the preliminary analyses suggest the significant differences in resultant efficacy of tested agents depending on whether SPI or MPI model was used, and further examinations are highly encouraged.

The dosage of the agents used to induce PH might influence the replicability of the result in human models. The most commonly-used agent, monocrotaline (MCT). After its activation to the reactive pyrrole metabolite dehydromonocrotaline (MCTP) in the liver via cytochrome P-450 (CYP3A4) it provokes the development of “MCT syndrome” manifested as acute lung injury interstitial pulmonary fibrosis, pulmonary hypertension with right ventricle hypertrophy and myocarditis [17, 18]. As currently explored, the median number of experimental days in the reviewed protocols was 28. In this case the disease progression toward death might be too short for compensatory mechanisms to develop. This has been addressed by reducing the dose of MCT (20–30 mg/kg), which is known to cause compensated right ventricle hypertrophy. In humans such compensatory mechanism initially allow the right ventricle to withstand the increase in after-load [19]. The minimal dose of monocrotaline that was used in approximately 250 of the reviewed experiments was 40 mg/kg bw (about 5 % of all MCT-linked models) and it was not possible to determine the effect of lower doses, i.e. 20 or 30 mg/kg bw, on the overall result of experiment. When doses of MCT ranging from 40 to 80 mg/kg bw were considered, neither survival analysis nor subgroup meta-analyses indicated that dosage schedules had any significant impact on the development of PH-linked lesions in the Vehicle group or on the response to potential therapeutic agents in the Intervention group. It is possible that both the length of PH induction, and as a consequence, duration of the experiment as a whole, had a stronger influence on the results obtained in the reviewed studies with doses of MCT ranging from 40 to 80 mg/kg bw. The current findings indicate that prolonging the induction period beyond 28 days was accompanied with further development of PH in Vehicle animals. Induction periods longer that 35 days were associated with less significant PH induction than shorter durations, however.

This phenomena can be at least partially explained by increased animal mortality caused by the toxicity of the substances used to induce pulmonary hypertension. It is likely that the animals with extremely developed pathophysiological profiles died before the end of the experiment, and the final analysis involved subjects that respond less to the procedures leading to PH or develop compensated right ventricle hypertrophy. This fact could account for the study heterogeneity and presence of over- or underestimations in finding, e.g. the results of the fitted meta-regression found the Vehicle animals with poorer PH-linked parameters but increased cumulative survival rate. Similarly, more reproducible data concerning reversal or prevention of PH-linked lesions in the Intervention group can be obtained when the experiment lasted from 14 to 28 days.

Next, it is possible that the choice of a preventive or reversal study regimen can also influence the final outcome. The majority of hemodynamic and hypertrophic parameters demonstrated normalization especially when potential agents for PH were tested in preventive rather than therapeutic models [8], as indicated in previous study. A current analysis of Kaplan-Meier plots reveals significant differences in animal mortality between these two regimens in favor of the preventive ones.

The main characteristic of PH is exercise intolerance, and therefore, assessments of animal capacity seem to be a valuable contribution to the common experiments that are performed in PH models. In humans, such exercise tests include the six-minute walk test (6MWT). The 6MWT is a repeatable test and plays a key role in the evaluation and treatment of PH patients, together with symptom monitoring, assessment of functional class or hemodynamic parameters, and measurement of biological markers. 6MWT is also an independent determinant of survival. Currently, the therapeutic goal for 6MWT in PH patients has been accepted to be 380–440 m [11].

Despite the crucial role played by exercise intolerance assessments in PH patients, only a few protocols (2 %) established for animal PH models have included such measurements in current review. In addition, these single exercise tests have been performed according to a variety of protocols as no standardized animal model of exercise test currently exists for PH. The most popular was the treadmill running test. It was performed in a continuous manner with fixed or progressively increasing parameters such as inclination, speed and duration that differed between particular protocols. Another method was the modified forced swimming test. The animals were placed in a cylinder beaker, of defined height and diameter, filled with water. Only two authors reported about using a preliminary phase of adaptation to certain modality of exercise. The experiments were performed by requiring the animal to run at some submaximal speed/work rate until fatigue, or exhaustion, i.e. the inability to keep pace with the treadmill. In one experiment, maximal oxygen uptake (VO_2_) was monitored and the highest VO_2_ achieved on a maximal exercise test was reported. Despite a variety of experimental protocols, the induction of PH reduced the exercise capacity in a statistically significant manner, while the potential medication agents significantly decreased exercise intolerance. Again, the important determinant of final outcome was the induction period and duration of the whole experiment.

Dosage schedules, including dose size and route of administration, can also be considered as potential source of heterogeneity between studies. The present survey demonstrates that the agent was most commonly administered intragastrically and, in one quarter of all studies, in drinking water; then the observed effect was the weakest. This poor response may be associated with the animals used in the studies not being housed separately: in this case, the amount of drug given in drinking water could not be strictly controlled by the researcher. The administered doses of the test agents appeared to vary widely. In addition, according to the United States Food and Drug Administration (USFDA) recommendations for dosing based on surface area, the applied doses exceeded those used for humans by 5–10 times or more [20]. Such wide dispersions might result from errors made with isometric scaling and direct extrapolation from animals to humans on a mg/kg basis. The FDA recommend that the correct dose of a drug should be proportional to the surface area of the two species rather than their body weight.

Present results are in line with those of Lythgoe et al. (2016) [5]. The authors propose that unsatisfactory outcome observed for statins in treating PH may, at least partially, be attributed to the improper selection of agent dose for animal experiments. Allosteric modeling indicates that the doses of simvastatin used in animal studies translated into 22 to 220 mg/day in humans, while the maximum licensed dose of simvastatin was 80 mg daily. These higher doses, being less tolerated, may also be insufficient to effectively inhibit farnesyltransferase, which in turn is an essential component of the statin-related beneficial effects in PH.

Previous studies have highlighted the problem associated with the limited application of data from pre-clinical studies as evidence supporting the progression to clinical trials, due to their possible lack of reproducibility and various forms of research bias that incorrectly estimate the final outcome [4]. They note the inability to completely replicate causes of human pulmonary hypertension and its manifestations, need for transparent reporting of experiment design and the importance of using standardized techniques to ensure robust data [21, 22]. Nevertheless, the careful planning of experimental design according to limitations of each model, as well as combining in vivo animal experiments with in vitro human cell and tissue studies, could provide more appropriate identification of novel promising substances for human diseases.

The current survey only discusses some methodological items that should be taken into consideration when establishing the study protocol to minimize the risk of wrongly estimating the final outcome regarding the efficacy of tested agents. There are several potential weaknesses to the present study that should be mentioned. First, the present survey is based on the analysis of data that were extracted from the literature according to the particular inclusion and exclusion criteria, that is why therefore it cannot completely represent all the researches studies in the area of pulmonary hypertension. The evaluated models varied according to their impact on PH-linked parameters. According to the previous findings the author proposes that a response ratio should be used as a measure of drug efficiency rather than net changes in measurements between vehicle and treated group. Although the proposed approach reduced the study heterogeneity, the measurement of response ratio did not eliminate all crucial items that could be responsible for study bias. To account for the enormous variation that characterizes the particular models of PH, settings in which PH was induced acutely or experiments on genetically manipulated animals were excluded. Next, as the examined studies used different protocols to evaluate efficacy of candidate agents, it was not possible to analyze and compare “head to head” some data findings from trials that have been performed according to specific type and length of induction, experimental period, dosage schedule, and etc. Another limitation is that in the majority of monocrotaline-based experiments, the animals were exposed to MCT at 50 and 60 mg/kg bw. Hence, it was not possible to evaluate the impact of smaller doses or the possible compensatory effects for PH development. Finally, due to small number of experiments that included exercise test, were not able to analyze the relationship between main outcome and animal capacity, nor the impact of a particular PH model on the degree of exercise intolerance.

## Conclusions

The animal models of PH used for pre-clinical studies are diverse: there are several methodological items within the established protocols that can determine the obtained result. In details it can be concluded that:

- Although SPI models based on single MCT injection or chronic hypoxia remain the most popular option for pre-clinical studies in PH, MPI models are recommended to better reflect the severity of PH observed in humans. The preliminary analyses suggest significant differences in the resultant efficacy of a particular substance was observed, depending on whether “one-hit” or “two-hit” model was used. Further head-to-head comparisons between these two approaches are highly encouraged.
- The preventive and reversal protocols can differ according to the final outcomes. The former can display better animal survival, and more promising results according to normalization of haemodynamic and hypertrophic parameters can be obtained. This might be a consequence of a shorter experimental period and less pronounced induction of PH-related changes.
- The methods used for PH induction should allow for the development of compensatory mechanisms rather than increase animal mortality. Prolonged induction periods can worsen animal survival when higher doses of PH inductor are used. Reducing the dose of inductor (e.g., MCT) and extending the experimental period might result in development of such mechanisms and better mimic human disease. Further studies on such protocols are encouraged.
- Isometric scaling instead of allometric one might result in overdosing and overestimation of the true intervention effect. In addition, there is a risk of wide dispersion of final outcome between protocols and poor study reproducibility. Substances administered to animals in drinking water may produce weaker effects as compared to the other routes.
- The results of the exercise test could vary significantly with regard to the chosen method. Any standardization of exercise tests according to such points as method to assess exercise capacity (e.g., forced swimming test, motor-driven treadmill, etc.), effort to overcome, the end-point measurement (e.g., until the rat fatigue, response to electric stimulus, etc.) or data about initial adaptation to the effort, would help establish an appropriate protocol for PH studies.

**Table:** 

For better reproducibility of studies on pulmonary hypertension, - the particular animal model should reflect categorization of human disease as regards future indication of potential therapies - it is recommended for experiments to be carried out according to both preventive and therapeutic protocols - in cases where doses of MCT range from 40 to 80 mg/kg bw, the induction of pulmonary hypertension should not exceed 28–35 days; any further increase in MCT dose does not seem to determine final outcome to the same degree as length of PH induction - animal mortality should be closely monitored and reported - the animal doses should be applied according to the allometric scaling - the route of drug administration could mimic a further dosage schedule in humans; intragastric route of agent administration should be chosen instead of per os, in case where the amount of drug given in drinking water could not be strictly controlled - the researcher might consider performing the exercise test; the correlation between the normalization of hemodynamic or hypertrophic parameters and resultant animal capacity would be a valuable addition to the obtained result.	

## Supplementary Information

ESM 1 (DOC 1273 kb)
