# Supplementary material for: Toward Better Reproducibility in Experimental Research on New Agents for Pulmonary Hypertension. An Analysis of Data from Four Hundred Animal Studies
Source: Cardiovasc Drugs Ther. 2020 Dec 9;35(4):707–18. doi: 10.1007/s10557-020-07109-3 (PMC8266793; doi:10.1007/s10557-020-07109-3)
Supplement: Supplementary file 1 — (DOC 1273 kb) [file 10557_2020_7109_MOESM1_ESM.doc]

**Supplementary data.**

**Table 1.** Study characteristics report * – concerns more than one comparison in the study.

| **Author** | **Year** | **Species** | **PH animal model** | **Protocol** | **Inductor dose (mg/kg bw)** | **Human PH indication (class)** | **Substance** | **Daily dose**  **(per kgbw)** | **Route** |
| --- | --- | --- | --- | --- | --- | --- | --- | --- | --- |
| Abid S | 2012 | Mouse | Chronic Hypoxia | Preventive | - | None | LP533401  Citalopram | 250 mg  10 mg | *i.g.*  *i.g.* |
| Agard C | 2009 | Rat | Monocrotaline/  Chronic Hypoxia* | Preventive | 60/  - | 1 | Metformin | 100 mg | *i.p.* |
| Ahmed LA | 2014 | Rat | Monocrotaline | Preventive | 60 | 1 | Diosgenin | 100 mg | *per os* |
| Ahmed LA | 2014 | Rat | Monocrotaline | Preventive | 60 | None | L-Arginine  Naringenin | 500 mg  50 mg | *per os*  *per os* |
| Aiello RJ | 2017 | Rat | Monocrotaline/  SU5416+ Chronic Hypoxia | Reversal | 60/  20 | 1 | KAR5417 | 50, 100  or 200 mg | *per os* |
| Akagi S | 2015 | Rat | Monocrotaline | Preventive | 60 | 1 | Imatinib | 1 mg | *i.t.* |
| Alencar AK | 2013 | Rat | Monocrotaline | Preventive | 60 | 1 | LASSBio-1359 | 50 mg | *i.g.* |
| Alencar AK | 2014 | Rat | Monocrotaline | Reversal | 60 | 1 | LASSBio-1386 | 50 mg | *i.g.* |
| Alencar AK | 2018 | Rat | Monocrotaline | Reversal | 60 | None | LASSBio-1359 | 34 or 170 µmol | *per os* |
| Alencar AK | 2017 | Rat | Monocrotaline | Reversal | 60 | None | G1 - G protein-coupled estrogen receptor (GPER) agonist | 0.4mg | *s.c.* |
| Alencar AK | 2018 | Rat | Monocrotaline | Reversal | 60 | None | G1- G protein-coupled estrogen receptor (GPER) agonist | 0.4mg | *s.c.* |
| Al-Hiti H | 2013 | Rat | Chronic Hypoxia | Preventive | - | 3 | L-Arginine | 500 mg | *i.g.* |
| Ambade AS | 2018 | Rat | Monocrotaline | Preventive | 60 | 1 | BIBF1000 | 50 mg | *per os* |
| Amirjanians M | 2017 | Rat | Monocrotaline | Reversal | 60 | 1 | BAY 41-8543 | 10 mg  1 or 3 mg | *per os*  *i.t.* |
| Andersen CU | 2005 | Rat | Chronic Hypoxia | Preventive | - | None | Molsidomine | 15 mg | *per os* |
| Atlı Ö | 2016 | Rat | Monocrotaline | Reversal | 60 | None | Everolimus | 3 mg | *i.g.* |
| Bai Y | 2017 | Rat | Monocrotaline | Preventive | 60 | 1 | 4-Chronic hypoxialoro-DL-phenylalanine (PCPA) | 50 or 100 mg | *i.p.* |
| Bai Y | 2017 | Rat | Monocrotaline | Preventive | 60 | 1 | 4-Chronic hypoxialoro-DL-phenylalanine (PCPA) | 50 or 100 mg | *i.p.* |
| Bai Y | 2014 | Rat | Monocrotaline | Preventive | 60 | 1 | 4-Chloro-DL-phenylalanine (PCPA) | 50 mg | *i.v.* |
| Baliga RS | 2008 | Rat | Chronic Hypoxia | Preventive | - | 3 | Ecadotril | 60 mg | *i.g.* |
| Banasová A | 2008 | Rat | Chronic Hypoxia | Preventive | - | 3 | Disodium cromoglycate | 40 mg | *i.p.* |
| BauerEM | 2014 | Mouse | Chronic Hypoxia | Preventive | - | None | Interferon alpha-2b | 10 000 IU | *s.c.* |
| Behringer A | 2016 | Rat | Monocrotaline | Reversal | 60 | 1 | Pioglitazone | 2.5 mg | *per os* |
| Bhat L | 2017 | Rat | Monocrotaline | Preventive | 60 | 1 | RP5063 (serotonin receptor modulator) | 2, 6  or 20 mg | *i.g.* |
| Bhat L | 2017 | Rat | Monocrotaline | Preventive | 60 | 1 | RP5063 (serotonin receptor modulator) | 20 mg | *i.g.* |
| Bi LQ | 2013 | Rat | Monocrotaline | Preventive | 60 | 1 | Ruscogenin | 0.1, 0.4  or 0.7 mg | *per os* |
| Blumberg FC | 2001 | Rat | Chronic Hypoxia | Reversal | - | 3 | Molsidomine | 15 mg | *per os* |
| Boehm M | 2018 | Mouse | Su5416 +Chronic Hypoxia | Reversal | 20 | 1 | Eplerenone | 0.1% | *per os* |
| Bogaard HJ | 2010 | Rat | Su5416+Chronic Hypoxia /Monocrotaline* | Reversal | 20/  60 | 1 | Carvedilol | 15 mg | *i.g.* |
| Bombicz M | 2017 | Rat | Monocrotaline | Preventive | 60 | 1 | Allium ursinum | 2% | *per os* |
| Bonnet S | 2003 | Rat | Chronic Hypoxia | Preventive | - | 3 | Dehydroepiandrosterone (DHEA) | 30 mg | *per os* |
| Breitling S | 2015 | Rat | Monocrotaline | Preventive | 40 | 1 | cAng (1–7) | 30, 100, 300 or 900 µg | *s.c.* |
| Bruce E | 2015 | Rat | Monocrotaline | Reversal | 50 | None | Compound 21 (C21) | 0.3 mg | *i.p.* |
| Bubb KJ | 2014 | Mouse | Chronic Hypoxia | Reversal | - | None | BAY 60-7550 | 10 mg | *i.g.* |
| Campos C | 2017 | Rat | Monocrotaline | Reversal | 60 | 1 | Copaiba Oil | 400 mg | *i.g.* |
| Campos-Carraro | 2018 | Rat | Monocrotaline | Reversal | 60 | 1 | Copaiba Oil | 400 mg | *i.g.* |
| Cha SA | 2018 | Rat | Monocrotaline | Preventive | 50 | 1 | Angiotensin-(1–9) | 0.58 mg | *s.c.* |
| Chabert C | 2018 | Rat | Chronic Hypoxia | Reversal | - | 3 | I-BET151 (Bromodomain inhibitor) | 10 mg/ml | *i.g.* |
| Chan MC | 2011 | Rat | Chronic Hypoxia | Preventive | - | 1 | Phenamil | 15 or 30 mg | *s.c.* |
| Chang H | 2018 | Rat | Monocrotaline | Preventive | 60 | 1 | Magnolol | 10 mg | *i.p.* |
| Chang LT | 2008 | Rat | Monocrotaline | Reversal | 75 | 1 | Cilostazol | 10 or 20 mg | *per os* |
| Chaumais MC | 2014 | Rat | Monocrotaline | Reversal | 60 | None | N-Acetylcysteine | 500 mg | *i.g.* |
| Chen F | 2018 | Rat | Chronic Hypoxia | Preventive | - | 1 | 3-Bromopyruvate | 15 mg | *i.p.* |
| Chen F | 2018 | Rat | Monocrotaline | Preventive | 60 | 1 | Grape seeds | 10 ml | *i.p.* |
| Chen L | 2011 | Rat | Monocrotaline | Preventive | 60 | 1 | Angiotensin (1–7) | 24 µg/h | *s.c.* |
| Chen R | 2017 | Mouse | Apoe+Chronic Hypoxia | Reversal | - | 3 | Apelin | 10 nmol | *i.p.* |
| Chen R | 2018 | Rat | Monocrotaline | Reversal | 60 | None | Docosahexaenoic acid | 100 mg | *i.p.* |
| Chen WS | 2012 | Mouse | Chronic Hypoxia | Preventive | - | 3 | Vardenafil | 10 mg | *i.p.* |
| Chen X | 2003 | Rat | Chronic Hypoxia | Preventive | - | 3 | Montelukast | 10 mg | *i.g.* |
| Chen XY | 2008 | Rat | Monocrotaline | Preventive | 60 | None | m-Nisoldipine | 0.5, 1  or 2 mg | *i.g.* |
| Chen YC | 2016 | Rat | Monocrotaline | Preventive | 60 | 1 | Salvianolic acid A | 0.3, 1 or 3 mg | *per os* |
| Cheng D | 1996 | Rat | Chronic Hypoxia | Preventive | - | 3 | Ginkgolide B | 5 mg | *i.p.* |
| Cheng Y | 2018 | Rat | Monocrotaline | Preventive | 60 | 1 | Isosorbide 5-mononitrate  Bardoxolone Methyl | 0.78 μg  2.1 μg | *inh*  *inh* |
| Chung HH | 2010 | Rat | Monocrotaline | Preventive | 60 | 1 | KMUP-1 | 5 or 1 mg | *per os/i.p.* |
| Church AC | 2015 | Rat | Monocrotaline/  Chronic Hypoxia* | Preventive | 60/  - | None | SB203580 PH-797804 | 20 mg | *i.p.* |
| Courboulin A | 2012 | Rat | Monocrotaline | Preventive | 60 | 1 | Plumbagin | 4 mg | *i.g.* |
| Cowan KN | 2000 | Rat | Monocrotaline | Reversal | 60 | None | ZD0892 M249314 | 240 mg | *i.g.* |
| Crossno JT | 2007 | Rat | Chronic Hypoxia | Preventive | - | 1 | Rosiglitazone | 5 mg | *per os* |
| Csiszar A | 2009 | Rat | Monocrotaline | Preventive | 60 | 1 | Resveratrol | 25 mg | *i.g.* |
| Dahal BK | 2010 | Rat/  Mouse | Monocrotaline/  Chronic Hypoxia* | Reversal | -  - | None | Azaindole-1 | 10 mg | *i.g.* |
| Dai G | 2018 | Rat | Monocrotaline | Preventive | 60 | None | Oxymatrine | 25, 50  or 100 mg | *per os* |
| Dai L | 2011 | Rat | Chronic Hypoxia | Preventive | - | 3 | Atorvastatin | 10 mg | *i.g.* |
| Dai ZK | 2010 | Rat | Monocrotaline | Preventive | 60 | 1 | KMUP-1 | 5 mg | *i.p.* |
| de Lima-Seolin BG | 2018 | Rat | Monocrotaline | Reversal | 60 | 1 | Bucindolol | 2 mg | *i.p.* |
| De Man FS | 2012 | Rat | Monocrotaline | Reversal | 60 | 1 | Bisoprolol | 10 mg | *i.g.* |
| De Raaf MA | 2014 | Rat | Su5416  +Chronic Hypoxia | Preventive | 20 | 1 | Trichronic hypoxiaostatin A | 450 mg | *i.p.* |
| Dean A | 2016 | Rat | Su5416+Chronic Hypoxia | Reversal | 20 | 1 | Metformin | 100 mg | *i.g.* |
| Delbeck M | 2011 | Rat | Monocrotaline | Preventive | 60 | 1 | Rivaroxaban  Warfarin  Enoxaparin | 9 or 30 mg  0.1-0.2 mg  20 or 60 mg | *per os*  *per os*  *s.c.* |
| Dumitrascu R | 2011 | Rat | Monocrotaline | Preventive | 60 | 1 | Terguride | 0.8 or 2.4 mg | *i.p.* |
| Eba S | 2013 | Mouse | Chronic Hypoxia | Preventive | - | 3 | Oltipraz | 50 mg | *i.g.* |
| Elias-Al-Mamun M | 2014 | Rat | Monocrotaline | Preventive /Reversal | 60 | None | Fasudil | 30 mg | *per os* |
| Elmedal B | 2004 | Rat | Chronic Hypoxia | Preventive | - | 3 | Molsidomine | 15 mg | *per os* |
| Fan Z | 2015 | Rat | Chronic Hypoxia | Preventive | - | 3 | YM155 | 6.5 mg | *s.c.* |
| Farhat M | 1993 | Rat | Monocrotaline | Preventive | 60 | None | 17β-estradiol | 10 mg | *s.c.* |
| Faul JL | 2000 | Rat | Left Pneumonoctomy  +Monocrotaline* | Preventive | 60 | 1 | Triptolide | 0.25 mg | *i.p.* |
| Feng Z | 2018 | Rat | Monocrotaline | Preventive | 60 | 1 | Acidic oligosaccharides | 10-20 mg | *i.p.* |
| Ferreira AJ | 2009 | Rat | Monocrotaline | Preventive | 50 | None | XNT | 0.12 mg | *s.c.* |
| Fowler ED | 2018 | Rat | Monocrotaline | Reversal | 60 | 1 | Metoprolol | 10 mg | *per os* |
| Fowler ED | 2018 | Rat | Monocrotaline | Reversal | 60 | 1 | Metoprolol | 10 mg | *per os* |
| Francis BN | 2018 | Rat | Monocrotaline | Preventive | 60 | None | Tetrahydrobiopterin (BH4 ) | 10 or 100 mg | *per os* |
| Frump AL | 2017 | Rat | Chronic Hypoxia | Preventive | - | 3 | 17β-estradiol | 3 mg | *s.c.* |
| Frump AL | 2015 | Rat | Su5416  +Chronic Hypoxia* | Preventive | 20 | 1 | 17β-estradiol4,4’,4”[4-Propyl-(1H)-pyrazole-1,3,5-triyl] trisphenol (PPT) diarylpropionitrile (DPN) | 0.075 mg  0.075 mg | *s.c.*  *s.c.* |
| Gao H | 2018 | Rat | Monocrotaline | Preventive | 60 | 1 | Acetylsalicylic acid  PD98059 | 2.5 mg  1 mg | *i.p.*  *i.p.* |
| Gao H | 2012 | Rat | Monocrotaline | Reversal | 50 | 1 | Quercetin | 100 mg | *No data* |
| Garat CV | 2013 | Rat | Chronic Hypoxia | Preventive | - | 1 | LY294002  Triciribine | 1 mg  0.5 mg | *s.c.*  *s.c.* |
| Gary-Bobo G | 2010 | Rat | Monocrotaline/  Chronic Hypoxia | Preventive | 60  - | None  3 | Amprenavir  Ritonavir  Nelfinavir | 100 mg  30 mg  500 mg | *i.g.*  *i.g.*  *i.g.* |
| Geng J | 2016 | Rat | Chronic Hypoxia | Preventive | - | 3 | Sarpogrelate | 50 mg | *i.g.* |
| Girgis RE | 2007 | Rat | Chronic Hypoxia | Preventive | - | 3 | Simvastatin | 20 mg | *i.g.* |
| Girgis RE | 2003 | Rat | Chronic Hypoxia | Preventive | - | 3 | Simvastatin | 20 mg | *per os* |
| Green DE | 2012 | Mouse | Chronic Hypoxia | Reversal | - | 3 | GKT137831  Rosiglitazone | 30-60 mg  10 mg | *i.g.*  *i.g.* |
| Grzegorzewska AP | 2017 | Rat | Su5416 +Chronic Hypoxia | Reversal | 20 | 1 | Dimethyl fumarate | 90 mg | *i.p.* |
| Guerard P | 2006 | Rat | Monocrotaline | Preventive | 60 | None | Pravastatin | 10 mg | *per os* |
| Guignabert C | 2005 | Rat | Monocrotaline | Preventive | 60 | None | GR127935  RS12744  Ketanserin  Fluoxetine | 2 mg  2 mg  2 mg  2 or 10 mg | *i.g*  *i.g*  *i.g*  *i.g.* |
| Guo S | 2017 | Rat | Chronic Hypoxia | Preventive | - | 1 | TRAM-34 | 0.3 or 0.6 mg | *i.p.* |
| Gupta N | 2017 | Rat | Su5416 +Chronic Hypoxia | Reversal | 20 | 1 | Fasudil | 3 or 5 mg | *i.t.* |
| Hamidi S | 2011 | Rat | Monocrotaline | Reversal | 60 | 1 | Vaso  active intestinal peptide (VIP) | 0.5 mg | *i.p.* |
| Hampl V | 2003 | Rat | Chronic Hypoxia | Preventive | - | 3 | Dehydroepiandrosterone (DHEA) | 0.009 mg | *per os* |
| Hansen MS | 2017 | Rat | Su5416 +Chronic Hypoxia | Reversal | 25 | 1 | Levosimendan | 3 mg | *per os* |
| Hara Y | 2011 | Mouse | Chronic Hypoxia | Reversal | - | 1 | MK571 | 5 or 25 mg | *per os* |
| Harrington LS | 2010 | Rat | Chronic Hypoxia | Preventive | - | 3 | GW0742 | 30 mg | *i.g.* |
| He Q | 2018 | Rat | Chronic Hypoxia | Preventive | - | 3 | Tsantan Sumtang | 0.3, 0.6, 0.9 or 1.2 mg/mL | *per os* |
| He Y | 2015 | Rat | Chronic Hypoxia | Reversal | - | 1 | Quercetin | 1 mg | *i.g.* |
| He YY | 2016 | Rat | Chronic Hypoxia | Preventive | - | 3 | Salubrinal | 1 mg | *i.p.* |
| Henriques-Coelho T | 2006 | Rat | Monocrotaline | Reversal | 60 | None | Ghrelin | 0.2 mg | *s.c.* |
| Herget J | 2003 | Rat | Chronic Hypoxia | Preventive | - | 3 | Batimastat | 30 mg | *i.p.* |
| Himori K | 2017 | Rat | Monocrotaline | Preventive | 60 | None | EUK-134 | 3 mg | *i.p.* |
| Hironaka E | 2006 | Rat | Monocrotaline | Preventive | 40 | 1 | Sarpogrelate | 50 mg | *i.p.* |
| Honda J | 2018 | Mouse | Chronic Hypoxia | Preventive | - | 3 | Liraglutide | 0.2 mg | *i.p.* |
| Hongo M | 2005 | Rat | Monocrotaline | Preventive | 50 | 1 | Nicorandil | 5, 7.5  or 10 mg | *per os* |
| Hosokawa S | 2013 | Rat | Monocrotaline | Reversal | 60 | 1 | IMD-0354 | 10 mg | *i.p.* |
| Houssaini A | 2013 | Rat | Monocrotaline | Preventive | 60 | 1 | Fluoxetine  Imatinib  Rapamycin | 20 mg  100 mg  5 mg | *i.g.*  *i.g*  *i.g* |
| Hsu WL | 2018 | Rat | Monocrotaline | Preventive | 60 | 1 | Baicalein | 50 mg | *per os* |
| Hu H | 2012 | Rat | Chronic Hypoxia | Preventive | - | 1 | P1 (11-amino acid synthetic peptide) | 60 mg | *i.p.* |
| Hu Y | 2018 | Rat | Monocrotaline | Preventive | 60 | 1 | Alginate oligosaccharides (AOS) | 5, 10 or 20 mg | *i.p.* |
| Hua C | 2018 | Rat | Chronic Hypoxia | Preventive | - | 1 | Apple polyphenol (APP) | 20 mg | *i.p.* |
| Huang X | 2014 | Rat | Chronic Hypoxia | Preventive | - | 1 | Aminoimidazole-4-carboxamide riboside (AICAR) | 1 mg | *i.p.* |
| Huang X | 2015 | Mouse | Chronic Hypoxia | Preventive | - | 1 | Salidroside | 16, 32,  or 64 mg | *i.p.* |
| Huang YF | 2009 | Rat | Chronic Hypoxia | Reversal | - | 3 | Sodium tanshinone IIA sulphonate (STS) | 10 mg | *i.p.* |
| Huang Z | 2016 | Rat | Monocrotaline | Reversal | 60 | 1 | GP130 | 5 mg | *i.p.* |
| Huh JW | 2011 | Mouse | Chronic Hypoxia | Preventive | - | 1 | YC-1 | 5 mg | *i.p.* |
| Hung MW | 2017 | Rat | Chronic Hypoxia | Preventive | - | 3 | Melatonin | 10 mg | *i.p.* |
| Ichimura K | 2018 | Rat | Monocrotaline | Reversal | 60 | 1 | Pitavastatin | 1, 3, 10  or 30 mg | *i.v.* |
| Inoue H | 2002 | Rat | Monocrotaline | Preventive | 70 | None | T-1032  Nitroglicerin | 1, 10 or 100 µg  0.1, 1 or 10 µg | *i.v.*  *i.v.* |
| Ishikawa K | 1995 | Rat | Monocrotaline | Preventive | 50 | None | Enalapril | 25 mg | *i.g.* |
| Jasińska-Stroschein M | 2014 | Rat | Monocrotaline | Reversal | 60 | None | Fasudil  Imatinib | 30 mg  50 mg | *i.g.*  *i.g* |
| Jasińska-Stroschein M | 2013 | Rat | Monocrotaline | Reversal | 60 | None | Fasudil | 30 mg | *i.g.* |
| Jasińska-Stroschein M | 2015 | Rat | Monocrotaline | Reversal | 60 | None | Imatinib | 50 mg | *i.g.* |
| Jasińska-Stroschein M | 2015 | Rat | Monocrotaline | Reversal | 60 | None | Imatinib  Rosuvastatin  Simvastatin | 20 or 50 mg  10 mg  20 mg | *i.g.*  *i.g*  *i.g* |
| Jasińska-Stroschein M | 2014 | Rat | Monocrotaline | Reversal | 60 | None | Rosuvastatin | 10 mg | *i.g.* |
| Jeffery TK | 2001 | Rat | Chronic Hypoxia | Preventive | - | 3 | Amlodipine  Perindopril | 10 mg  30 mg | *i.g.*  *i.g.* |
| Jeffery TK | 1999 | Rat | Chronic Hypoxia | Preventive | - | None | Perindopril | 3, 10  or 30 mg | *i.g.* |
| Jiang H | 2013 | Rat | Monocrotaline | Reversal | 50 | 1 | Fasudil | 15 mg | *i.p.* |
| Jiang L | 2012 | Rat | Monocrotaline | Preventive | 60 | 1 | Pinacidil  Simvastatin | 2 mg  20 mg | *i.g.*  *i.g.* |
| Jiang Y | 2018 | Rat | Chronic Hypoxia | Preventive | - | 1 | Topotecan | 1 or 10 mg | *i.g.* |
| Jin H | 2016 | Rat | Chronic Hypoxia | Preventive | - | 3 | Grape seed procyanidin extract (GSPE) | 10 ml | *i.g.* |
| Jin H | 2014 | Rat | Chronic Hypoxia | Preventive | - | 3 | Melatonin | 15 mg | *i.p.* |
| Kameshima S | 2015 | Rat | Monocrotaline | Preventive | 60 | 1 | A-484954 | 2.5 mg | *i.p.* |
| Kanai Y | 1993 | Rat | Monocrotaline | Preventive | 60 | None | DV-7028 | 2 or 20 mg | *i.g.* |
| Kanno S | 2001 | Rat | Monocrotaline | Preventive | 40 | 1 | Enalapril | 4.4 mg | *per os* |
| Kataoka M | 2005 | Rat | Monocrotaline | Preventive | 60 | None | ONO-1301 | 20 mg | *s.c.* |
| Kato T | 2008 | Rat | Monocrotaline | Preventive | 50 | None | Olmesartan medoxomil | 2 or 5 mg | *per os* |
| Kazama K | 2014 | Rat | Monocrotaline | Preventive | 60 | 1 | Omentin | 18 µg | *i.p.* |
| Kim EK | 2010 | Rat | Chronic Hypoxia | Preventive | - | 1 | Rosiglitazone | 8 mg | *i.p.* |
| Kim SY | 2012 | Rat/  Mouse | Monocrotaline/  Chronic Hypoxia* | Reversal | 60/  - | 1 | Bortezomib | 1 or 100 µg | *i.p.* |
| Klein M | 2008 | Rat | Monocrotaline | Reversal | 60 | None | Sorafenib  Imatinib | 10 mg  50 mg | *i.g.*  *i.g.* |
| Klinger JK | 1998 | Rat | Chronic Hypoxia | Preventive | - | 3 | Brain natriuretic peptide (BNP)  Atrial natriuretic peptide (ANP) | 0.2 or 1.4  µg/h | *s.c.*  *s.c.* |
| Klinke A | 2014 | Mouse | Chronic Hypoxia | Preventive | - | 3 | 10-nitro-oleic acid (OA-NO_2_) | 1.04 nmol/g/h | *s.c.* |
| Kojonazarov B | 2013 | Rat | Monocrotaline | Reversal | 60 | 1 | Sorafenib  Sunitinib | 10  1 or10 mg | *i.g.*  *i.g.* |
| Kosanovic D | 2011 | Rat | Monocrotaline | Reversal | 60 | None | TBC3711 | 30 mg | *i.g.* |
| Koyama M | 2014 | Mouse | Chronic Hypoxia | Reversal | - | 1 | 4-Phenylbutyric acid | 350-550 mg | *per os* |
| Kwon JL | 2013 | Rat | Monocrotaline | Preventive | 60 | 1 | Infliximab | 5 mg | *s.c.* |
| Lahm T | 2012 | Rat | Chronic Hypoxia | Preventive | - | 3 | 17β-estradiol | 0.075 mg | *s.c.* |
| Lai YL | 1998 | Rat | Chronic Hypoxia | Preventive | - | 3 | Fullerenol-1  U-83836E  DMTU-1  DMTU-2 | 10 mg  10 mg  10 mg  10 mg | *i.p.*  *i.p.*  *i.p.*  *i.p.* |
| Lai YL | 1995 | Rat | Chronic Hypoxia | Preventive | - | 3 | Capsaicin | 150 mg | *s.c.* |
| Lan B | 2015 | Rat | Monocrotaline  +Chronic Hypoxia* | Preventive | 60/  - | 3 | Valproic acid | 300 mg | *i.g.* |
| Lan TH | 2018 | Rat | Monocrotaline | Preventive | 60 | 1 | 3,7-Bis(2-hydroxyethyl)icaritin | 10, 20. or 40 mg | *i.g.* |
| Laudi S | 2007 | Rat | Monocrotaline | Preventive | 60 | None | Atorvastatin | 0.1, 1  or 10 mg | *i.g.* |
| Laursen BE | 2008 | Rat | Chronic Hypoxia | Preventive | - | 3 | L-Arginine | 2.5 g | *per os* |
| Lee DS | 2010 | Rat | Monocrotaline | Preventive | 60 | 1 | Simvastatin | 2 mg | *No data* |
| Lee DS | 2018 | Rat | Monocrotaline | Preventive | 60 | 1 | Cyclosporine | 10 mg | *per os* |
| Lee JH | 2016 | Rat | Monocrotaline | Preventive | 60 | 1 | KR36676 | 30 mg | *i.g.* |
| Lee MY | 2016 | Rat | Monocrotaline | Preventive | 60 | 1 | Liraglutide | 0.15 mg | *no data* |
| Lee Y | 2018 | Rat | Monocrotaline | Preventive | 60 | 1 | Cerivastatin | 0.2 mg | *i.t. twice a week* |
| Leong ZP | 2018 | Rat | Monocrotaline | Preventive | 60 | 1 | Imatinib  Sunitinib | 5, 15 or 50 mg, 0.3, 1, 3  or 10 mg | *i.g.*  *i.g.* |
| Li C | 2018 | Rat | Chronic Hypoxia | Preventive | - | 1 | Fasudil | 30 mg | *i.p.* |
| Li G | 2012 | Rat | Left Pneumonoctomy +Monocrotaline* | Preventive | 40 | 1 | Resorcinolnaphthalein | 120 µg/d | *s.c.* |
| Li G | 2013 | Rat | Left Pneumonoctomy +Monocrotaline* | Preventive | 40 | 1 | Resorcinolnaphthalein | 120 µg/d | *s.c.* |
| Li H | 2014 | Rat | Monocrotaline | Preventive | 60 | 1 | Telmisartan | 10 mg | *i.g.* |
| Li J | 2012 | Rat | Chronic Hypoxia | Preventive | - | 3 | U50,488H | 1.25 mg | *i.g.* |
| Li J | 2009 | Rat | Chronic Hypoxia | Preventive | - | 3 | U50,488H | 1.25 mg | *i.p.* |
| Li J | 2013 | Rat | Monocrotaline | Preventive | 50 | 1 | Iptakalim | 1, 3  or 9 mg | *i.p.* |
| Li L | 2016 | Rat | Chronic Hypoxia | Preventive | - | None | Carthamus tinctorius L. | 25 mg | *i.g.* |
| Li S | 2016 | Rat | Monocrotaline | Preventive | 60 | 1 | Metformin | 150 mg | *i.p.* |
| Li XL | 2011 | Rat | Monocrotaline | Preventive | 50 | 1 | Rosuvastatin | 2 or 10 mg | *i.g.* |
| Li XL | 2012 | Rat | Monocrotaline | Preventive | 50 | 1 | Rosuvastatin | 2 or 10 mg | *i.g.* |
| Li XQ | 2006 | Rat | Monocrotaline | Preventive | 60 | None | Sertraline | 10 mg | *i.g.* |
| Li XW | 2014 | Rat | Monocrotaline | Preventive | 60 | None | Rutecarpine | 20 or 40 mg | *i.g.* |
| Li XW | 2015 | Rat | Monocrotaline | Preventive | 60 | None | Sesamin | 50 or 100 mg | *No data* |
| Li XW | 2015 | Rat | Monocrotaline | Preventive | 60 | 1 | Chrysin | 50 or 100 mg | *s.c.* |
| Li XW | 2015 | Rat | Monocrotaline | Preventive | 60 | 1 | Chrysin | 50 mg | *s.c.* |
| Li Y | 2018 | Rat | Left Pneumonoctomy+ Monocrotaline | Preventive | 50 | 1 | Osthole | 10  or 20 mg | *i.g.* |
| Li ZC | 2002 | Rat | Monocrotaline | Preventive | 60 | None | DCDDP  Nitrendipine | 0.005, 0.05 or 0.5 mg  10 mg | *i.p.*  *i.p.* |
| Lima-Seolin BG | 2017 | Rat | Monocrotaline | Reversal | 60 | 1 | Bucindolol | 2 mg | *i.p.* |
| Lin Z | 2017 | Rat | Monocrotaline | Preventive | 40 | 1 | Atorvastatin | 5 or 10 mg | *i.g.* |
| Lingeshwar P | 2016 | Rat | Monocrotaline | Preventive | 60 | None | Vigabatrin | 50 or 75 mg | *per os* |
| Li-sheng Li | 2016 | Rat | Monocrotaline | Reversal | 50 | None | Icariin | 20, 40  or 80 mg | *i.g.* |
| Liu A | 2017 | Mouse | Su5416  +Chronic Hypoxia | Preventive | 20 | None | Estrogen | 0.075 mg | *s.c.* |
| Liu A | 2017 | Mouse | Su5416  +Chronic Hypoxia | Preventive | 20 | 1 | Estrogen | 0.1 mg | *s.c.* |
| Liu B | 2014 | Rat | Chronic Hypoxia | Preventive | - | 1 | Trimethoxystilbene  Resveratrol | 5, 10 mg  25 mg | *i.g.*  *i.g.* |
| Liu B | 2008 | Rat | Left Pneumonoctomy +Monocrotaline* | Preventive | 60 | 1 | Simvastatin | 2 mg | *i.g.* |
| Liu B | 2009 | Rat | Left Pneumonoctomy +Monocrotaline* | Preventive | 60 | 1 | Simvastatin | 2 mg | *i.g.* |
| Liu C | 2014 | Rat | Monocrotaline | Preventive | 60 | 1 | Ethyl pyruvate | 50 mg | *i.p.* |
| Liu J | 2018 | Rat | Chronic Hypoxia | Preventive | - | 3 | Fibroblast growth factor 21 | 0.1 mg | *s.c.* |
| Liu M | 2014 | Rat | Monocrotaline/ Chronic Hypoxia* | Reversal | 60/  - | None | Fasudil | 10, 30  or 75 mg | *i.g.* |
| Liu P | 2015 | Rat | Chronic Hypoxia | Preventive | - | 3 | Baicalin | 30 mg | *i.p.* |
| Liu Y | 2010 | Rat | Chronic Hypoxia | Preventive | - | 3 | Amiloride | 1, 3 or 10 mg | *per os* |
| Liu Y | 2012 | Rat | Monocrotaline | Preventive | 60 | 1 | Rosiglitazone | 10 mg | *i.g.* |
| Liu Y | 2013 | Rat | Monocrotaline | Preventive | 60 | 1 | Sarpogrelate | 150 mg | *i.g.* |
| Liu ZQ | 2011 | Rat | Left Pneumonoctomy +Monocrotaline* | Preventive | 60 | 1 | Simvastatin | 2 mg | *No data* |
| Lu Y | 2017 | Rat | Monocrotaline | Preventive | 40 | None | Valsartan | 20 or 40 mg | *i.g.* |
| Luan Y | 2015 | Rat | Monocrotaline | Reversal | 60 | 1 | Baicalin | 100 mg | *i.g.* |
| Ma W | 2011 | Rat | Monocrotaline | Reversal | 60 | None | MDL28170 | 20 mg | *i.p.* |
| MacRitchie N | 2016 | Rat | Chronic Hypoxia | Preventive | - | 1 | RB-005  PF-543 | 10 mg  1 mg | *i.p*  *i.p.* |
| Mao SZ | 2009 | Rat | Chronic Hypoxia | Preventive | - | None | Apelin | 10 nmol | *s.c.* |
| Marcos E | 2003 | Mouse | Chronic Hypoxia | Preventive | - | 3 | Citalopram  Fluoxetine  GR127935  Ketanserin | 10 mg  10 mg  2 or 10 mg  2 mg | *i.g.*  *i.g.*  *i.p.*  *i.p.* |
| Maron BA | 2012 | Rat | Monocrotaline | Reversal | 50 | 1 | Spironolactone | 25 mg | *per os* |
| Matori H | 2012 | Rat | Monocrotaline | Reversal | 60 | None | Genistein | 1 mg | *s.c.* |
| Mawatari E | 2007 | Rat | Monocrotaline | Preventive | 40 | 1 | Amlodipine | 6 mg | *per os* |
| Maxová H | 2010 | Rat | Chronic Hypoxia | Preventive | - | 3 | Disodium cromoglycate | 40 mg | *i.p.* |
| Maxová H | 2011 | Rat | Chronic Hypoxia | Reversal | - | 3 | Disodium cromoglycate | 40 mg | *i.p.* |
| McMurtry M | 2007 | Rat | Monocrotaline | Reversal | 60 | 1 | Rapamycin | 2.5 mg | *i.g.* |
| Medarametla V | 2014 | Rat | Monocrotaline/  Left Pneumomnectomy  +Monocrotaline* | Reversal | 60 | 1 | PK10453  Imatinib | 20 µg  40 µg | *inh*  *inh* |
| Megalou AJ | 2010 | Rat | Monocrotaline | Reversal | 60 | 1 | T9429 | 5 mg | *i.v.* |
| Megalou AJ | 2012 | Rat | Monocrotaline | Reversal | 60 | 1 | Antibody against TGF‑β‑ligand | 0.1 mg | *s.c.* |
| Meghwani H | 2018 | Rat | Monocrotaline | Preventive | 50 | None | Ocimum sanctum (Linn) | 200 mg | *per os* |
| Meghwani H | 2017 | Rat | Monocrotaline | Reversal | 50 | None | Terminalia arjuna (Roxb.) | 125  or 250 mg | *per os* |
| Mei Y | 2011 | Rat | Monocrotaline | Reversal | 50 | 1 | Urantide | 10 μg | *i.p.* |
| Mendes-Ferreira P | 2016 | Rat | Monocrotaline | Reversal | 60 | 1 | rhNRG-1 | 0.04 mg | *i.p.* |
| Mielakis ED | 2002 | Rat | Chronic Hypoxia | Reversal | - | 3 | Diloroacetate | 70 mg | *per os* |
| Mitani Y | 1997 | Rat | Chronic Hypoxia/  Monocrotaline | Preventive | - | 3  60 | L-Arginine  D-Arginine | 500 mg  500 mg | *i.p.*  *i.p.* |
| Miyata M | 2000 | Rat | Monocrotaline | Preventive | 40 | None | Sarpogrelate | 30 mg | *i.p.* |
| Morales-Cano D | 2014 | Rat | Monocrotaline | Reversal | 60 | 1 | Quercetin | 10 mg | *i.g.* |
| Morecroft I | 2010 | Mouse | Chronic Hypoxia | Preventive | - | 1 | LY393558  Citalopram | 30 mg  20 mg | *i.g.*  *i.g.* |
| Morel OE | 2003 | Rat | Chronic Hypoxia | Preventive | - | 3 | Nifedipine | 40 mg | *i.g.* |
| Morin C | 2014 | Rat | Monocrotaline | Preventive | 60 | None | Docosapentaenoic acid monoacylglyceride | 231 mg | *i.g.*  *i.g.* |
| Mouchers KT | 2010 | Rat | Monocrotaline | Reversal | 40 | 1 | Fasudil | 100 mg | *i.g.* |
| Mourelle M | 2011 | Rat | Monocrotaline | Preventive | 60 | 1 | LA-419 | 30 mg | *per os* |
| Murata T | 2005 | Rat | Chronic Hypoxia | Preventive | - | 3 | Fluvastatin | 1 mg | *per os* |
| Murugesan P | 2015 | Rat | Left Pneumonoctomy +Monocrotaline* | Preventive | 60 | 1 | BI113823 | 30 mg | *i.g.* |
| Nagata T | 1997 | Rat | Monocrotaline | Preventive | 60 | None | OKY-046 (ozagrel)  ONO-8809 | 50 mg  1 mg | *i.g.*  *i.g.* |
| Nagaya N | 2003 | Rat | Monocrotaline | Preventive | 60 | None | Adrenomedullin | 5 µg | *Inh* |
| Nakamura A | 2013 | Rat | Monocrotaline | Preventive | 60 | 1 | ONO-1301 | 10 mg | *i.g.* |
| Nakata TM | 2015 | Rat | Monocrotaline | Preventive | 60 | 1 | Pimobendan  Nicorandil | 0.15 mg  1 mg | *per os*  *per os* |
| Nan X | 2017 | Rat | Chronic Hypoxia | Preventive | - | 1 | Rhodiola algida | 62.5, 125. or 250 mg | *per os* |
| Nassar SZ | 2018 | Rat | Monocrotaline | Reversal | 60 | 1 | Cerium oxide | 0.1  or 0.15 mg | *i.p. twice a week* |
| Nickel NP | 2015 | Rat | Su5416  +Chronic Hypoxia * | Reversal | 20 | 1 | Elafin | 0.2 mg | *s.c.* |
| Nisbet RE | 2010 | Mouse | Chronic Hypoxia | Reversal | - | 3 | Rosiglitazone | 10 mg | *i.g.* |
| Nishida M | 2009 | Rat | Monocrotaline | Reversal | 60 | None | Raloxifene | 10 mg | *i.g.* |
| Nishimura T | 2001 | Rat | Left Pneumonoctomy +Monocrotaline * | Preventive | 60 | None | 40-O-(2-hydroxyethyl)-rapamycin (RAD) | 2.5 mg | *i.g.* |
| Nishimura T | 2002 | Rat | Left Pneumonoctomy +Monocrotaline * | Preventive | 60 | 1 | Simvastatin | 2 mg | *i.g.* |
| Nogueira-Ferreira R | 2017 | Rat | Monocrotaline | Preventive | 60 | 1 | Terameprocol | 166 mg | *i.p.* |
| Nong Z | 1996 | Rat | Chronic Hypoxia | Preventive | - | 3 | Quinapril | 1 mg | *i.g.* |
| Okada K | 1998 | Rat | Left Pneumonoctomy +Monocrotaline * | Preventive | 60 | None | Quinapril  Losartan | 30 mg  40 mg | *per os*  *per os* |
| Onat AM | 2013 | Rat | Monocrotaline | Preventive | 60 | 1 | Palosuran | 600 mg | *i.g.* |
| Ono S | 1992 | Rat | Chronic Hypoxia | Preventive | - | 3 | Bepafant (WEB 2170)  BN 50739 | 10 mg  10 mg | *i.g.*  *i.g.* |
| Ono S | 1992 | Rat | Monocrotaline | Preventive | 60 | None | Bepafant (WEB 2170) | 10 mg | *i.g.* |
| Ou ZJ | 2010 | Rat | Monocrotaline | Preventive | 50 | 1 | L-Arginine | 500 mg | *i.p.* |
| Özlem A | 2017 | Rat | Chronic Hypoxia | Preventive | - | 3 | Everolimus  Alagebrium | 3 mg  10 mg | *i.g.*  *i.g.* |
| Paffett ML | 2012 | Rat | Monocrotaline | Reversal | 50 | 1 | Dexamethasone | 17.5 or 50 µg | *i.p.* |
| Paffett ML | 2012 | Rat | Monocrotaline | Reversal | 50 | 1 | Resveratrol | 3 mg | *per os* |
| Pankey EA | 2013 | Rat | Monocrotaline | Reversal | 60 | None | Imatinib | 50 mg | *i.p.* |
| Pankey EA | 2012 | Rat | Monocrotaline | Reversal | 60 | None | NaNO2 | 3 mg | *i.p.* |
| Pankova NV | 2017 | Rat | Chronic Hypoxia | Preventive | - | 3 | 17β-estradiol | 15 pg | *s.c.* |
| Pehlivan Y | 2014 | Rat | Monocrotaline | Preventive | 60 | 1 | Palosuran | 30 or 100 mg | *i.g.* |
| Pei JM | 2006 | Rat | Chronic Hypoxia | Preventive | - | 3 | U50,488H | 1.25 mg | *i.g.* |
| Pei Y | 2011 | Rat | Monocrotaline | Preventive | 60 | None | Rosuvastatin | 5 mg | *per os* |
| Pena A | 2017 | Rat | Su5416  +Chronic Hypoxia | Reversal | 20 | 1 | PP242 | 20 mg | *i.p.* |
| Pereira SL | 2013 | Rat | Monocrotaline | Reversal | 60 | 1 | LASSBio-1289 | 50 or 75 mg | *i.g.* |
| Perros F | 2015 | Rat | Monocrotaline | Reversal | 60 | 1 | Nebivolol  Metoprolol | 10 mg  10 or 100 mg | *No data*  *No data* |
| Pichon A | 2012 | Rat | Chronic Hypoxia | Reversal | - | 3 | Acetazolamide | 40 mg | *per os* |
| Pidgeon GP | 2004 | Rat | Chronic Hypoxia | Preventive | - | 3 | SC236  Ifetroban | 3 mg  50 mg | *per os*  *per os* |
| Poble PB | 2018 | Rat | Su5416 +Chronic Hypoxia | Preventive | 20 | 1 | Pirfenidone | 30 mg | *per os* |
| Polonio IB | 2014 | Rat | Monocrotaline | Preventive | 60 | 1 | Lodenafil | 5 mg | *per os* |
| Porvasnik SL | 2010 | Rat | Monocrotaline | Reversal | 40 | 1 | PRX-08066 | 50 or 100 mg | *i.g.* |
| Preston IR | 2013 | Mouse/  Rat | Chronic Hypoxia /Monocrotaline* | Preventive | -/  60 | 1 | Spironolactone | 15 mg | *s.c.* |
| Price LC | 2011 | Rat | Monocrotaline | Reversal | 60 | 1 | Dexamethasone | 1.25 or 5 mg | *i.p.* |
| Prins KW | 2017 | Rat | Monocrotaline | Reversal | 60 | 1 | Colchicine | 0.5 mg | *i.p.* |
| Pullamsetti S | 2005 | Rat | Monocrotaline | Reversal | 60 | 1 | Tolafentrine | 0.12 or 2 mg | *Inh* |
| Pullamsetti SS | 2011 | Rat | Monocrotaline | Reversal | 60 | 1 | Tolafentrine | 0.12 mg | *Inh* |
| Puukila S | 2017 | Rat | Monocrotaline | Reversal | 60 | 1 | Secoisolariciresinol diglucoside | 25 mg | *i.g.* |
| Qi J | 2000 | Rat | Chronic Hypoxia | Preventive | - | 3 | L-Arginine | 500 mg | *i.p.* |
| Qi J | 2001 | Rat | Chronic Hypoxia | Preventive | - | 3 | L-Arginine | 500 mg | *i.p.* |
| Quinn DA | 1998 | Rat | Chronic Hypoxia | Preventive | - | 3 | Ethylisopropyl amiloride (EIPA) | 3 mg | *s.c.* |
| Rakotoniaina Z | 2008 | Rat | Monocrotaline | Preventive | 60 | None | Celecoxib  Atorvastatin | 25 mg  10 mg | *i.g.*  *i.g.* |
| Rakotoniaina Z | 2006 | Rat | Monocrotaline | Preventive | 60 | None | Pravastatin  Atorvastatin | 10 mg  10 mg | *per os*  *per os* |
| Rashid J | 2018 | Rat | Su5416 +Chronic Hypoxia | Preventive | 20 | 1 | Rosiglitazone | 5 mg | *i.t.* |
| Ribeiro EL | 2017 | Rat | Monocrotaline | Preventive | 60 | None | Diethylcarbamazine | 50 mg | *per os* |
| Rocchetti M | 2014 | Rat | Monocrotaline | Preventive | 60 | 1 | Ranolazine | 60 mg | *i.p.* |
| Sahara M | 2014 | Rat | Monocrotaline | Preventive | 60 | 1 | Nicorandil | 2.5, 5 or 7.5 mg | *s.c.* |
| Sakamoto Y | 2017 | Rat | Monocrotaline | Preventive | 60 | 1 | Vapsin | 1 μg | *i.p.* |
| Samillan V | 2013 | Mouse | Chronic Hypoxia | Reversal | - | 3 | Human recombinant erythropoietin (EPO) | 500 IU | *per os* |
| Satoh M | 2009 | Rat | Monocrotaline | Reversal | 70 | None | Atorvastatin  Simvastatin  Pravastatin | 2 mg  2 mg  4 mg | *per os*  *per os*  *per os* |
| Sawada H | 2007 | Rat | Monocrotaline | Preventive | - | None | Pyrrolidine dithiocarbamate (PDTC) | 100 mg | *s.c.* |
| Schermuly RT | 2004 | Rat | Monocrotaline | Reversal | 60 | None | Tolafentrine | 625 ng/kg per min | *i.v.* |
| Schreiber C | 2017 | Rat | Monocrotaline | Reversal | 60 | 1 | L-Arginine  Tetrahydrobiopterin | 300 mg  20 mg | *per os*  *per os* |
| Schwenke D | 2008 | Rat | Chronic Hypoxia | Preventive | - | 3 | Ghrelin | 0.15 mg | *s.c* |
| Segura-Ibarra V | 2017 | Rat | Monocrotaline | Preventive | 60 | 1 | Rapamycin | 15 mg | *i.g.* |
| Shen L | 2011 | Rat | Monocrotaline | Preventive | 60 | 1 | Acetylsalicylic acid | 0.5, 1, 2 or 4 mg | *i.g.* |
| Shi K | 2009 | Rat | Monocrotaline/  Chronic Hypoxia* | Preventive | 60/  - | None | Doxycycline | 20 mg | *i.g.* |
| Shi R | 2018 | Rat | Monocrotaline | Reversal | 60 | 1 | Baicalein | 50 or 100 mg | *per os* |
| Shi R | 2018 | Rat | Monocrotaline | Reversal | 60 | 1 | Baicalein | 50 or 100 mg | *per os* |
| Shi W | 2018 | Rat | Monocrotaline | Preventive | 60 | 1 | Resveratrol | 25 mg | *i.g.* |
| Sun LY | 2017 | Rat | Monocrotaline | Preventive | 50 | 1 | 5-Aminosalicylic Acid | 50, 100, 150  or 200 mg | *i.g.* |
| Sun X | 2008 | Rat | Monocrotaline | Preventive | 60 | 1 | Rosuvastatin | 2 mg | *i.g.* |
| Sun XZ | 2014 | Rat | Chronic Hypoxia | Preventive | - | 3 | Fasudil | 15 ml | *i.p.* |
| Sun XZ | 2015 | Rat | Chronic Hypoxia | Preventive | - | 3 | Fasudil | 30 mg | *i.p.* |
| Suzuki C | 2006 | Rat | Monocrotaline | Preventive | 60 | 1 | Mycophenolate mofetil (MMF) | 20 mg | *i.g.* |
| Suzuki R | 2012 | Rat | Monocrotaline | Preventive | 60 | None | γ-Aminobutyric acid (GABA) | 500 mg | *per os* |
| Takahashi T | 1996 | Rat | Monocrotaline | Preventive | 80 | 1 | E4021 | 10, 30  or 300 mg | *per os* |
| Takahashi T | 1995 | Rat | Monocrotaline | Preventive | 80 | 1 | Semotiadil  Diltiazem | 30 or 100 mg  100 or 300 mg | *per os*  *per os* |
| Tan JX | 2008 | Rat | Chronic Hypoxia | Reversal | - | 1 | Adenosine | 100 µg/kg min | *s.c.* |
| Tan JX | 2012 | Rat | Chronic Hypoxia | Reversal | - | 3 | Adenosine | 150 µg/kg min | *s.c.* |
| Tang B | 2014 | Rat | Monocrotaline | Preventive | 60 | 1 | Ellagic acid | 30 mg | *i.g.* |
| Tawa M | 2018 | Rat | Monocrotaline | Preventive | 60 | None | Isosorbine moonitrate  Sodium nitrate | 0.3 or 1 g/L  30 or 300 mg/L | *per os*  *per os* |
| Thompson JS | 1994 | Rat | Chronic Hypoxia | Preventive | - | 3 | SCH 42495 | 60 mg | *i.g.* |
| Thompson JS | 1994 | Rat | Chronic Hypoxia | Reversal | - | 3 | SCH 42495 | 60 mg | *i.g.* |
| Tian X | 2011 | Rat | Monocrotaline | Reversal | 60 | None | Papaverine | 1 µg/kg min | *s.c.* |
| Tofovic SP | 2010 | Rat | Monocrotaline | Reversal | 60 | 1 | 2-Ethoxyestradiol | 3, 10  or 30 µg/kg h | *s.c.* |
| Tofovic SP | 2008 | Rat | Monocrotaline | Reversal | 60 | None | 2-Hydroxyestradiol | 10 µg | *s.c.* |
| Tofovic SP | 2005 | Rat | Monocrotaline | Preventive | 60 | None | 2-Methoxyestradiol | 10 µg | *s.c.* |
| Tofovic SP | 2010 | Rat | Monocrotaline | Preventive | 60 | 1 | 2-Methoxyestradiol | 10 µg/kg h | *s.c.* |
| Tual L | 2006 | Rat | Chronic Hypoxia | Preventive | - | 3 | Propranolol  Prazosin  Carvedilol | 120 mg  12 mg  40 mg | *i.g.*  *i.g.*  *i.g.* |
| Türck P | 2018 | Rat | Monocrotaline | Reversal | 60 | 1 | Trapidil | 5 or 8 mg | *i.p.* |
| Umar S | 2011 | Rat | Monocrotaline | Reversal | 60 | None | 17β-estradiol  diarylpropionitrile (DPN) 4,4’,4”[4-Propyl-(1H)-pyrazole-1,3,5-triyl] trisphenol (PPT) | 42.5 µg  850 µg  850 µg | *s.c.* |
| Uzun O | 2006 | Rat | Chronic Hypoxia | Preventive | - | 3 | Erdosteine | 20 mg | *per os* |
| Vignozzi L | 2017 | Rat | Monocrotaline | Preventive | 60 | 1 | Obeticholic acid | 3, 10  or 30 mg | *i.g.* |
| Villegas LR | 2003 | Mouse | Chronic Hypoxia | Preventive | - | 3 | MnTE-2-PyP | 5 mg | *s.c.* |
| Wang AP | 2015 | Rat | Chronic Hypoxia | Preventive | - | 3 | Rapamycin | 1 mg | *i.g.* |
| Wang HL | 1997 | Rat | Monocrotaline | Preventive | 60 | None | Tetrandrine | 50, 100  or 150 mg | *i.g.* |
| Wang HM | 2012 | Rat | Monocrotaline | Reversal | 60 | 1 | Fluoxetine | 2 or 10 mg | *i.g.* |
| Wang HM | 2018 | Rat | Monocrotaline | Preventive | 60 | 1 | Cystamine | 20 or 40 mg | *per os* |
| Wang J | 2012 | Rat | Monocrotaline/ Chronic Hypoxia* | Preventive | 50/  - | 1 | Sodium tanshinone IIA sulphonate (STS) | 10 mg | *i.p.* |
| Wang L | 2017 | Rat | Chronic Hypoxia | Preventive | - | 3 | 17β-estradiol  2-methoxyestradiol | 0.02 mg  0.24 mg | *s.c.*  *s.c.* |
| Wang LX | 2009 | Rat | Chronic Hypoxia  +Hypercapnia* | Preventive | - | 3 | Oridonin | 10 mg | *i.p.* |
| Wang SH | 2013 | Rat | Monocrotaline | Preventive | 60 | 1 | Simvastatin | 2 mg | *i.g.* |
| Wang W | 2011 | Rat | Monocrotaline | Preventive | 50 | 1 | Dexamethasone  Pyrrolidine dithiocarbamate (PDTC) | 1 mg  100 mg | *i.p.*  *i.p.* |
| Wang X | 2018 | Rat | Chronic Hypoxia | Preventive | - | 3 | Asiaticoside | 50 mg | *i.g.* |
| Wang X | 2015 | Rat | Monocrotaline | Preventive | 50 | 1 | Tetrandrine  Vardenafil | 50 mg  2 mg | *i.p.*  *i.p.* |
| Wang XB | 2015 | Rat | Chronic Hypoxia | Preventive | - | 3 | Asiaticoside | 50 mg | *i.g.* |
| Wang XF | 2012 | Rat | Monocrotaline | Reversal | 60 | 1 | Rosiglitazone | 5 mg | *i.g.* |
| Wang Y | 2011 | Rat | Monocrotaline | Preventive | 60 | None | Fluoxetine | 2 or 10 mg | *i.g.* |
| Wang Y | 2011 | Rat | Monocrotaline | Preventive | 60 | 1 | Fluoxetine | 2 or 10 mg | *i.g.* |
| Wang Y | 2018 | Rat | Monocrotaline | Preventive | 50 | 1 | Urantide | 0.01 mg | *i.p.* |
| Wang YD | 2018 | Rat | Monocrotaline | Preventive | 60 | 1 | 17β-estradiol | 0.075 mg | *s.c.* |
| Wang YX | 2016 | Rat | Chronic Hypoxia | Reversal | - | 3 | Fasudil | 10, 30 or 75 mg | *i.g.* |
| Wei L | 2007 | Rat | Left Pneumonoctomy +Monocrotaline * | Preventive | 60 | None | Triptolide | 0.25 mg | *i.p.* |
| Weissmann N | 2009 | Mouse | Chronic Hypoxia | Reversal | - | 3 | HMR1766(Ataciguat) | 10 mg | *s.c.* |
| Wilson DN | 2016 | Rat | Monocrotaline | Preventive | 60 | 1 | Resveratrol | 25 mg | *s.c.* |
| Wisutthathum S | 2018 | Rat | Monocrotaline | Preventive | 60 | 1 | Eulophia macrobulbon | 15, 450  or 1000 mg | *per os* |
| Wu F | 2017 | Rat | Monocrotaline | Reversal | 60 | None | Aloperine | 25, 50  or 100 mg | *per os* |
| Wu F | 2017 | Rat | Monocrotaline | Reversal | 60 | None | Aloperine | 25, 50  or 100 mg | *per os* |
| Wu JR | 2015 | Rat | Chronic Hypoxia | Preventive | - | 3 | KMUP-1A | 5 mg | *i.g.* |
| Wu Q | 2013 | Rat | Chronic Hypoxia | Preventive | - | 3 | U50,488H | 1.25 mg | *i.p.* |
| Wu Y | 2016 | Rat | Monocrotaline | Preventive | 60 | 1 | 4-Phenylbutyric acid | 500 mg | *i.g.* |
| Xia XQ | 2004 | Rat | Chronic Hypoxia | Preventive | - | 3 | Urapidil | 10 mg | *i.g.* |
| Xie L | 2010 | Rat | Monocrotaline | Reversal | 40 | 1 | Atorvastatin  Losartan | 5 mg  50 mg | *i.g.*  *i.g.* |
| Xie W | 2004 | Rat | Chronic Hypoxia | Preventive | - | 3 | Iptakalim | 0.75 mg | *i.g.* |
| Xie X | 2015 | Rat | Monocrotaline | Preventive | 60 | 1 | Rosiglitazone | 5 mg | *per os* |
| Xin WX | 2018 | Rat | Monocrotaline | Preventive | 60 | 1 | Sceptridium ternatum | 2.5, 5, 10 g/kg of extract | *i.g.* |
| Xin Y | 2015 | Rat | Monocrotaline | Preventive | 60 | 1 | all-trans retinoic acid (ATRA) | 30 mg | *i.g.* |
| Xu DQ | 2010 | Rat | Chronic Hypoxia | Preventive | - | 3 | 17β-estradiol | 0.1 mg | *i.p.* |
| Xu Y | 2018 | Rat | Monocrotaline | Preventive | 60 | 1 | Capsaicin | 50, 100  or 150 mg | *s.c.* |
| Yamada Y | 2014 | Rat | Monocrotaline | Preventive | 60 | None | Human recombinant thrombomodulin | 1.5 mg | *s.c.* |
| Yan J | 2013 | Rat | Chronic Hypoxia | Preventive | - | 3 | Docosahexaenoic acid | 100 mg | *i.g.* |
| Yang DL | 2010 | Rat | Monocrotaline | Preventive | 50 | None | Resveratrol | 20 or 60 mg | *i.g.* |
| Yang H | 2000 | Rat | Chronic Hypoxia | Preventive | - | 3 | *Ginko biloba* glycosides | 200 mg | *i.g.* |
| Yang JM | 2017 | Rat | Monocrotaline | Reversal | 50 | 1 | Betaine | 100, 200  or 400 mg | *per os* |
| Yang L | 2008 | Rat | Chronic Hypoxia | Preventive | - | 3 | Breviscapine | 80 mg | *i.g.* |
| Yang PS | 2014 | Rat | Monocrotaline | Preventive | 60 | None | Glycyrrhizin | 50 mg | *i.p.* |
| Yavuz T | 2013 | Rat | Monocrotaline | Preventive | 60 | 1 | Pyrrolidine dithiocarbamate (PDTC) | 100 mg | *s.c.* |
| Ye JX | 2016 | Rat | Chronic Hypoxia | Preventive | - | 1 | Metformin | 180 mg | *No data* |
| Yin Y | 2013 | Rat | Left Pneumonoctomy +Monocrotaline * | Reversal | 60 | 1 | Paclitaxel | 2 mg | *i.v.* |
| Yu J | 2010 | Rat | Chronic Hypoxia | Preventive | - | 3 | Vasonatrin peptide (VNP) | 0.05 mg | *i.p.* |
| Yu L | 2017 | Rat | Chronic Hypoxia | Preventive | - | 1 | Resveratrol | 25 mg | *i.g.* |
| Yu L | 2015 | Rat | Left Pneumonoctomy +Monocrotaline * | Preventive | 60 | None | Rapamycin | 5 mg | *i.g.* |
| Yu W | 2017 | Rat | Monocrotaline | Preventive | 60 | 1 | N‑Acetylcysteine | 500 mg | *per os* |
| Yu W | 2017 | Rat | Monocrotaline | Preventive | 60 | 1 | N‑Acetylcysteine | 100  or 500 mg | *per os* |
| Yuan LB | 2017 | Rat | Monocrotaline | Reversal | 60 | 1 | Astragalus | 200 mg | *per os* |
| Yuyama H | 2005 | Rat | Chronic Hypoxia | Preventive | - | 3 | Nebentan (YM598) | 1 mg | *per os* |
| Zambelli V | 2011 | Rat | Monocrotaline | Preventive | 60 | None | Aminaftone | 30 or 150 mg | *per os* |
| Zapata-Sudo G | 2012 | Rat | Monocrotaline | Preventive | 60 | 1 | LASSBio-965 | 20 or 50 mg | *i.p.* |
| Zeng Z | 2002 | Rat | Monocrotaline | Preventive | 60 | None | Felodipine | 5 mg | *i.p.* |
| Zhai FG | 2009 | Rat | Monocrotaline | Preventive | 60 | 1 | Fluoxetine | 2 or 10 mg | *i.g.* |
| Zhang B | 2014 | Rat | Monocrotaline/  Chronic Hypoxia* | Preventive | 60/  - | 1 | Oxymatrine | 50 mg | *per os* |
| Zhang D | 2014 | Rat | Monocrotaline | Preventive | 60 | 1 | Rosiglitazone | 5 mg | *i.g.* |
| Zhang E | 2010 | Rat | Chronic Hypoxia | Preventive | - | 3 | all-trans retinoic acid (ATRA) | 50 mg | *i.g.* |
| Zhang E | 2015 | Rat | Chronic Hypoxia | Preventive | - | 3 | Sarpogrelate | 30 mg | *i.g.* |
| Zhang L | 2014 | Rat | Chronic Hypoxia | Reversal | - | 1 | Baicalin | 100 mg | *i.p.* |
| Zhang L | 2013 | Rat | Chronic Hypoxia | Preventive | - | 3 | U50,488H | 1.25 mg | *i.p.* |
| Zhang LL | 2014 | Rat | Monocrotaline | Preventive | 60 | 1 | Etanercept | 2.5 mg | *i.p. twice a week* |
| Zhang N | 2018 | Rat | Chronic Hypoxia | Preventive | - | 3 | Danshensu |  | *per os* |
| Zhang WH | 2009 | Rat | Monocrotaline | Preventive | 80 | 1 | Simvastatin | 2 mg | *i.g.* |
| Zhang WH | 2012 | Rat | Monocrotaline | Reversal | 60 | 1 | Simvastatin | 2 mg | *i.g.* |
| Zhang X | 2018 | Rat | Chronic Hypoxia | Reversal | - | 3 | Astragaloside IV | 10 or 50 mg | *i.p.* |
| Zhang Y | 2014 | Rat | Chronic Hypoxia | Preventive | - | 3 | Atorvastatin | 10 mg | *i.g.* |
| Zhang Y | 2015 | Rat | Monocrotaline | Preventive | 60 | 1 | DAPT | 10 mg | *i.p.* |
| Zhang Y | 2012 | Rat | Monocrotaline | Preventive | 60 | 1 | Genistein | 20 or 80 µg | *i.p.* |
| Zhang Y | 2017 | Rat | Monocrotaline | Preventive | 40 | None | Isoquercitrin | 0.1% | *per os* |
| Zhang YF | 2015 | Rat | Monocrotaline | Preventive | 60 | 1 | Mycophenolate mofetil (MMF) | 20 or 40 mg | *i.g.* |
| Zhang Z | 2017 | Rat | Monocrotaline | Preventive | 60 | None | Baicalin | 100 mg | *i.g.* |
| Zhao J | 2018 | Rat | Left Pneumonoctomy +Monocrotaline | Reversal | 60 | 1 | Paclitaxel | 2 mg | *i.v.* |
| Zhao L | 2009 | Rat | Chronic Hypoxia | Reversal | - | 1 | Simvastatin | 20 mg | *i.p.* |
| Zhao L | 1996 | Rat | Chronic Hypoxia | Preventive/Reversal | - | 3 | GR13 8950C  Sodium nitroprusside (SNP) | 1 mg  or 4.3 mg | *s.c.* |
| Zhao S | 2014 | Rat | Chronic Hypoxia | Preventive | - | 3 | *Panax notoginseng* saponins (PNS) | 50 mg | *i.p.* |
| Zheng L | 2015 | Rat | Chronic Hypoxia | Preventive | - | 3 | Sodium tanshinone IIA sulphonate (STS) | 10 mg | *i.p.* |
| Zhong X | 2000 | Rat | Chronic Hypoxia | Preventive | - | 3 | Pinacidil | 3 mg | *i.p.* |
| Zhou KR | 1993 | Rat | Monocrotaline | Preventive | 60 | None | Capsaicin | 50, 100  or 150 mg | *s.c.* |
| Zhou Q | 2017 | Rat | Chronic Hypoxia  +Hypercapnia | Reversal | - | None | Solnatide  Dexamethasone | 0.1-0.5 mg  0.3 mg | *i.t.*  *i.p.* |
| Zhou S | 2015 | Rat | Monocrotaline | Preventive | 60 | 1 | Resveratrol | 2.5 mg | *per os* |
| Zhu N | 2016 | Rat | Monocrotaline | Reversal | 60 | 1 | Thymoquinone | 8, 12  or 16 mg | *per os* |
| Zhu R | 2015 | Rat | Chronic Hypoxia | Preventive | - | 1 | Iptakalim | 1.5 mg | *i.g.* |
| Zhu R | 2015 | Rat | Monocrotaline | Preventive | 60 | 1 | Ruscogenin | 0.1, 0.4  or 0.7 mg | *per os* |
| Zhu S | 2009 | Rat | Monocrotaline | Preventive | 60 | 1 | Fluoxetine | 10 mg | *i.g.* |
| Zhu SP | 2017 | Rat | Monocrotaline | Reversal | 60 | 1 | Fluvastatin | 10 mg | *i.g.* |
| Zhu TT | 2017 | Rat | Chronic Hypoxia | Preventive | - | 3 | Epigallocatein-3-gallate | 50, 100  or 200 mg | *i.g.* |
| Zhu Y | 2017 | Rat | Monocrotaline | Preventive | 60 | 1 | MG-132  Bortezomib | 0.1 mg  0.1 mg | *i.p.*  *i.p.* |
| Zopf DA | 2011 | Rat | Monocrotaline | Preventive | 60 | 1 | C-122 | 1 or 10 mg | *i.g.* |
| Zuo X | 2011 | Rat | Chronic Hypoxia | Preventive | - | 3 | Iptakalim | 0.75 or 1.5 mg | *i.g.* |
| Zuo XR | 2012 | Rat | Monocrotaline | Preventive | 60 | 1 | Nicorandil | 7.5 mg | *i.g.* |

**Table 2.** Animal models used for testing of drug candidates in accordance with World Health Organisation's categorisation of human PH.

| **Animal model** | | **Percentage (%)** | **Categorization of human PH**  **class* (percentage**)** |
| --- | --- | --- | --- |
| SPI | MCT | 60.9 | 1 (67.6); no data (32.4) |
|  | CH | 34.4 | 1 (22.5); 3 (68.3); others (0.7); no data (8.5) |
| MPI | MCT+LP | 3.9 | 1 (68.8); no data (31.2) |
|  | MCT+CH | 0.2 | 3 (100) |
|  | SU-5416+CH | 3.7 | 1 (93.3); no data (6.7) |
|  | CH+hypercapnia | 0.5 | 3 (50.0); no data (50.0) |
|  | ApoE+CH | 0.2 | 3 (100) |
| Candidate treatment was tested on ≥2 animal models, and/or according to 2 protocols in the study | | 3.7 | - |

SPI – single pathological insult, MPI – multiple pathological insult, MCT – monocrotaline; CH – chronic hypoxia; LP – left pneumonectomy; ApoE – apolipoprotein E; * according to ESC comprehensive clinical classification of pulmonary hypertension (1 – pulmonary arterial hypertension; 3 – pulmonary hypertension due to lung diseases and/or hypoxia); ** according to particular model; SPI (R=0.48; 95% CI, 0.44-0.51) *vs.* MPI (R= 0.57; 0.52-0.62) ; P=0.0043. *N=409 studies*

**Table 3.** Characteristics of studies that reported impact of PH induction on the animal capacity.

| **Author** | **Year** | **PH animal model** | **Duration of PH induction (days)** | **Method to assess exercise capacity*** | **Effort to overcome** | **The end-point parameter** | **The end-point measurement** | **Initial adaptation** |
| --- | --- | --- | --- | --- | --- | --- | --- | --- |
| Alencar AK | 2014 | Monocrotaline | 1, 14 or 28 | Motor-driven tredmill | Progressively increasing treadmill speed: 8 m/min; 12 m/min; and 18 m/min. | Treadmill distance (mkg) | The running is maintained until the rat reach the point of fatigue, which is confirmed by loss of the animal righting reflex | no |
| Alencar AK | 2017 | Monocrotaline | 28 | Motor-driven tredmill | Progressively increasing treadmill speed: 8 m/min; 12 m/min; and 18 m/min. | Exercise duration (min) | The running is maintained until the rat reach the point of fatigue, which is confirmed by loss of the animal righting reflex | no |
| Bogaard HJ | 2010 | Sugen | 28 | Motor-driven tredmill | A treadmill with an inclination of 15° and speed of 15 m/min. | Exercise duration (min) | Exhaustion is established when the rats accept the electric stimulus 3 consecutive times as opposed to running | yes |
| Courboulin A | 2012 | Monocrotaline | 1, 7, 14, 21or 28 | Motor-driven tredmill | No detailed data | Treadmill distance (m) | No detailed data | no |
| Frump AL | 2015 | Sugen | 28 | Motor-driven tredmill | Progressively increasing treadmill speed and incline stages: 10 m/min at 0°, 10 m/min at 5°, followed by an increase by 5 m/min and 5° for each consecutive stage | Maximal oxygen uptake (ml/kgh) | The test is terminated when VO_2_ plateaued despite increasing workload, or if the rat is unable to maintain position on the treadmill belt despite three consecutive electrical shocks to the tail without recovery | yes |
| Koyama M | 2014 | Chronic hypoxia | 28 | Motor-driven tredmill | Progressively increasing treadmill speed from 1 min at 5 m/min to 8 min at 12 m/min, and finally 13 m/min until exhaustion. The slope of the treadmill was kept constant at 5° | Treadmill distance (m) | The running is maintained until exhaustion defined as spending 50% of the time in a stage or 3 consecutive seconds on the shock grid | no |
| Megalou AJ | 2010 | Monocrotaline | 14 | Forced swimming test | The animals are placed in a cylinder beaker (height: 50 cm; diameter: 30 cm) filled with water (25°C) to a height of 25 cm. | Exercise duration (min) | Swimming represents a time from immersion until near-drowning | no |
| Prins KW | 2017 | Monocrotaline | 28 | Motor-driven tredmill | Progressively increasing treadmill speed from 10 m/min and 5 m/min every 5 minutes | Treadmill distance (m) | The exercise is maintained for 30 minutes or until the rat fatigued | no |

**Table 4**. Dispersion of doses of example agents used in the experiments.

| **Substance** | **RSD (%)** |
| --- | --- |
| 17 beta-estradiol | 211.5 |
| Acetylsalicylic acid | 71.5 |
| Amiloride | 50.0 |
| Amlodipine | 25.0 |
| Atorvastatin | 62.8 |
| Bisoprolol | - |
| Carvedilol | 45.5 |
| Celecoxib | - |
| Cerivastatin | 33.3 |
| Ciclosporine | - |
| Cilostazol | 33.3 |
| Citalopram | 33.3 |
| Dexamethasone | 136.4 |
| Diltiazem | 50,0 |
| Doxycicline | - |
| Enalapril | 70.1 |
| Enoxaparin | 50.0 |
| Everolimus | - |
| Fasudil | 79.3 |
| Fluoxetine | 76.7 |
| Fluvastatin | 81.8 |
| Imatinib | 57.0 |
| Iptakalim | 109.9 |
| Losartan | 11.1 |
| Melatonin | 99.1 |
| Metformin | 23.1 |
| Metoprolol | 119.9 |
| m-nisoldipine | 53.4 |
| Molsidomine | - |
| Montelucast | - |
| Mycophenolate mofetil | 35.3 |
| Nebivolol | - |
| Nicorandil | 47.0 |
| Nifedipine | - |
| Nifedipine | - |
| Nitroglicerin | 120.8 |
| Perindopril | 65.8 |
| Pitavastatin | 104.24 |
| Pravastatin | 35.3 |
| Prazosin | - |
| Propranolol | - |
| Quercetin | 149.04 |
| Quinapril | 93.5 |
| Resveratrol | 67.1 |
| Rivaroxaban | 53.8 |
| Rosiglitazone | 32.1 |
| Rosuvastatin | 63.5 |
| Sertraline | - |
| Simvastatin | 105.4 |
| Sorafenib | - |
| Spironolactone | 25.0 |
| Sunitinib | 98.9 |
| Telmisartan | - |
| Valproic acid | - |

**Fig. 1.** Quorum diagram showing summary of study selection procedure.

**Titles identified through electronic databases (1991-2018):**

**Duplicates removed** n=373

**Medline** n=11,367

**Web of Science** n=13,877

**Inclusion based on titles and abstracts**

n=712

n=868

n=1,580

**Duplicates removed** n=647

**Inclusion based on abstracts**

n=933

**Abstracts excluded** n=131

**Full-text assessed for eligibility** n=802

**Articles excluded in meta-analysis n=393**

Criteria:

- outcomes not reported (n=154)
- pregnant, or newborn animals (n=51)
- acute treatment (n=26)
- not precisely defined dose of drugs (n=33)
- genetically manipulated animals (n=51)
- lack of the number of animals reported (n=14)
- comorbid animals (n=5)
- acute PH induction (n=7)
- other PH models: aortic constriction, bleomycin PH, schistosoma-induced PH (n=30)
- others (n=22)

**Articles included in meta-analysis n=409**

Criteria:

- unregistered drugs in PH
- chronic treatment
- chronic PH induction
- experiments *in vivo* on mammals
- positive and negative controls for intervention groups
- methods of PH induction: SPI and MPI

**Fig. 2.** Tree plot for the effect size: mean difference for Vehicle (MCT-treated rats) (a) and response ratio for Intervention (MCT+tested agent) (b) in accordance with different doses of monocrotaline (mg/kg bw) as PH inductor.

In general, the induction of PH reduced the animal pulmonary parameters as compared to the healthy rats (Sham) (a) (P<0.0005), while potential medication agents improved animal performance (P<0.0001) as compared to the Vehicle; the effect size of 0.5 means that the examined agents reduced the mean value of the parameter by only half (b). The different response to potential agents for PH was observed when a spectrum of doses of MCT was given (Q=48.24; df=3; P<0.0001) (b), without any clear trend. The relationship between monocrotaline dose and the strength of PH induction in Vehicle group was less pronounced (Q=7.53; df=3; P=0.0566) (a).


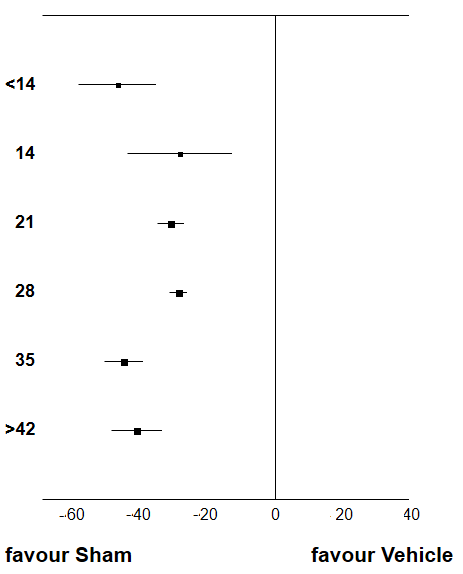

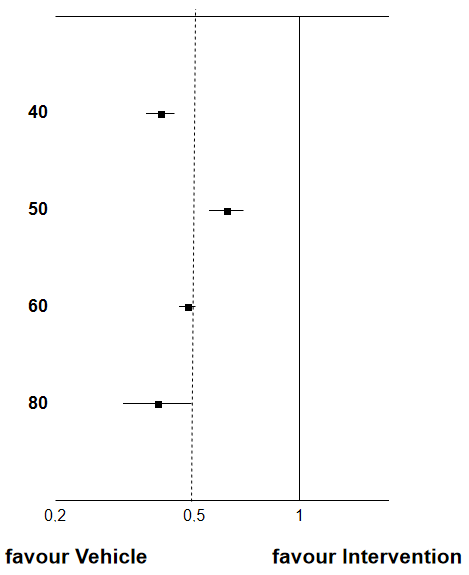


**b**

**a**

**Fig. 3.** Tree plot for the overall effect size (response ratio) in accordance with the agent administration period (a) (P=0.0271; Q=10.95; df=4) and the results of subgroup analyses for most most frequently tested agents. HMG-CoA reductase inhibitors (P=0.02) (b), RhoA/ROCK inhibitors (P=0.06) (c), Antiinflammatories (P=0.017) (d), Plant-derived substances (P>0.05) (e). An effect size of 0.5 indicates that the examined agents reduced the mean value of a particular parameter only by half. A response ratio at 1.0 would indicate an ability of an agent to completely reverse the altered PH parameters. The schedules shorter than seven days or longer than 28 days demonstrated poorer normalization of PH-related parameters by candidate drugs for pulmonary hypertension.


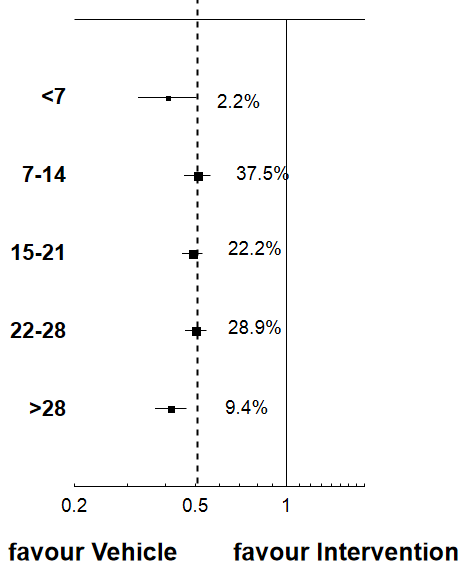

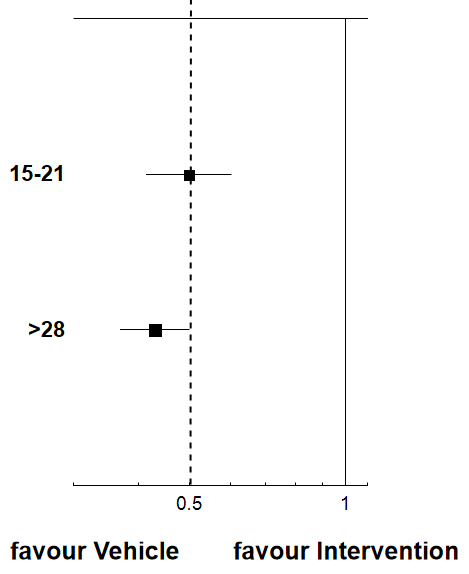

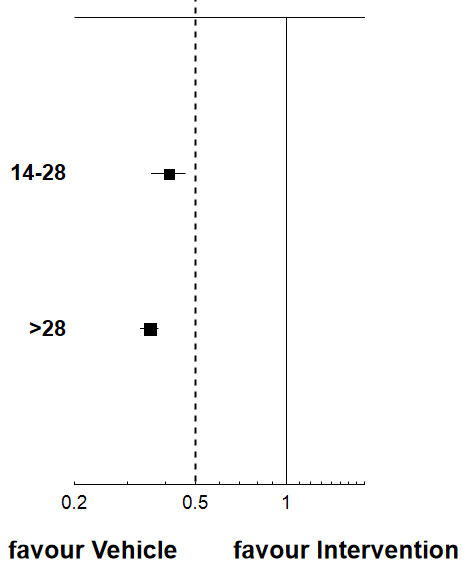


**a**

**b**

**c**


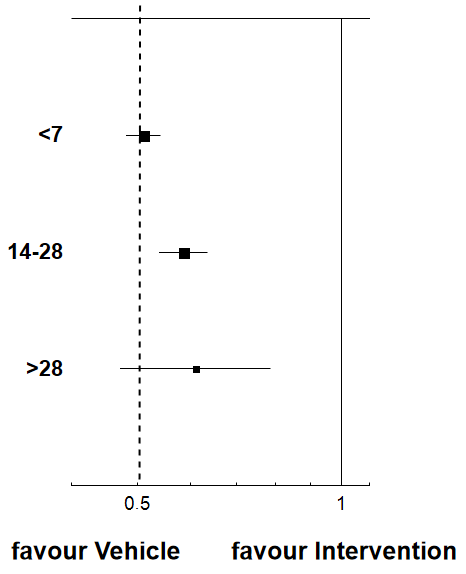

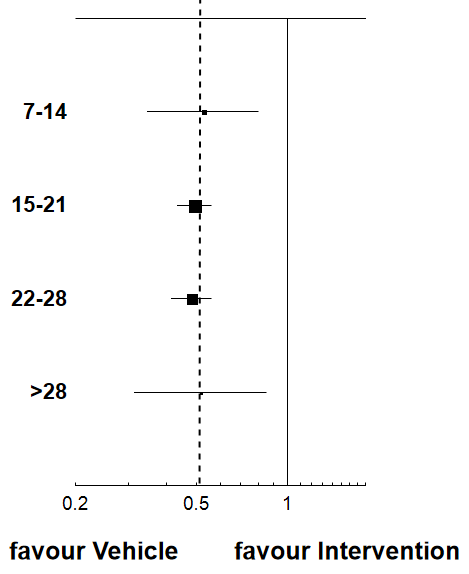


**d**

**e**

**Fig. 4.** The influence of type of anaesthesia on the animal hemodynamics (P<0.0001; Q=929,8; df=20). The tree plot demonstrates effect size (response ratio) in Intervention group in accordance with five most often used anaesthetics during hemodynamic measurements. The anesthetics except for chloral hydrate did not differentiate the response to tested drugs for PH. The animals that were examined under anesthesia by chloral hydrate were revealed to response the most. Then a response ratio tended toward values exceeding 0.5.


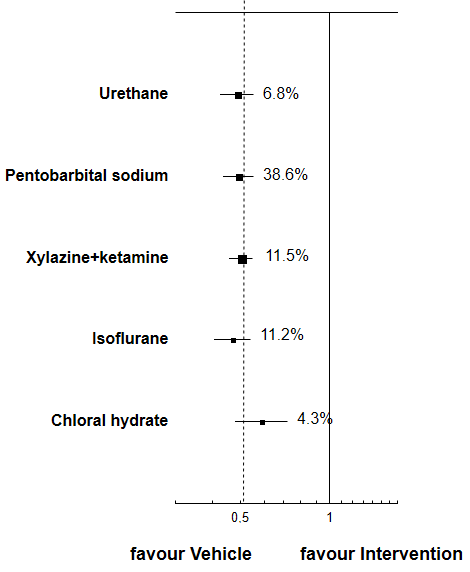


**Fig. 5.** Tree plot for the effect size (response ratio) with regard to route of agent administration – the results of subgroup analyses for most frequently tested agents. HMG-CoA reductase inhibitors (P<0.0001) (a), RhoA/ROCK inhibitors (P<0.0001) (b), Antiinflammatories (P<0.0001) (c), Plant-derived substances (P>0.05) (d), Ser/Thre inhibitors (P>0.05) (e). Selected agent that were administered orally or by gavage reduced the mean value of a particular parameter by less than half (response ratio, R<0.5); this means the relatively weakest effect regarding the reduction or prevention of PH-related changes.


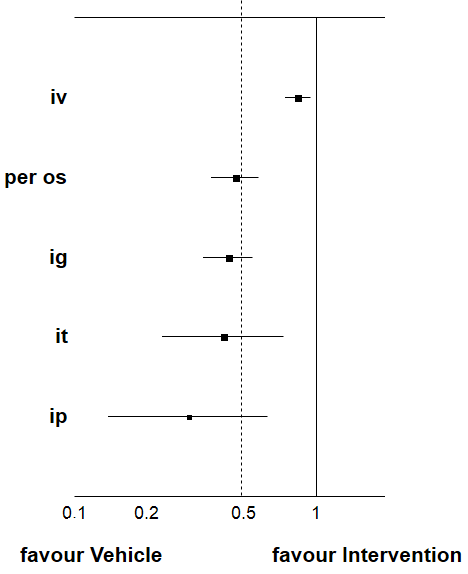

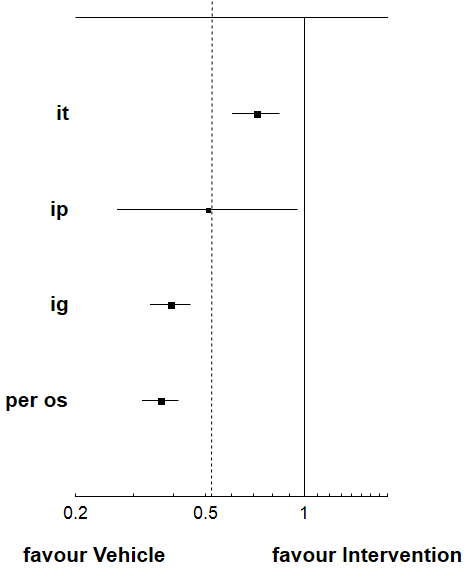


**b**

**a**


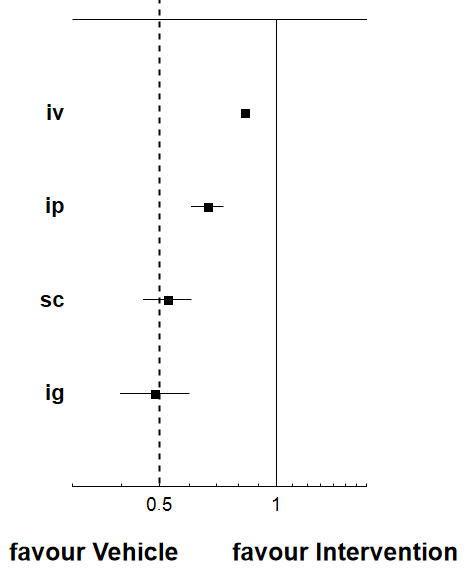

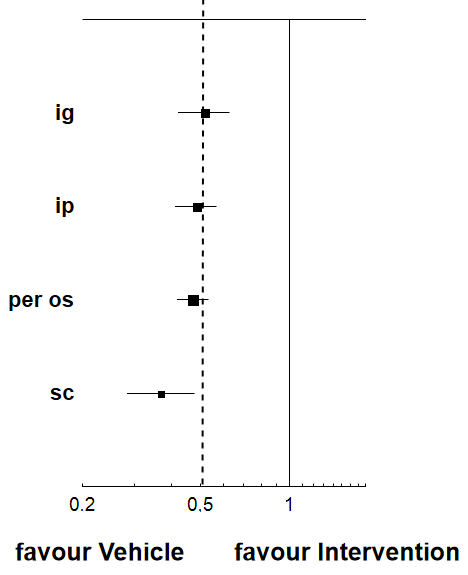

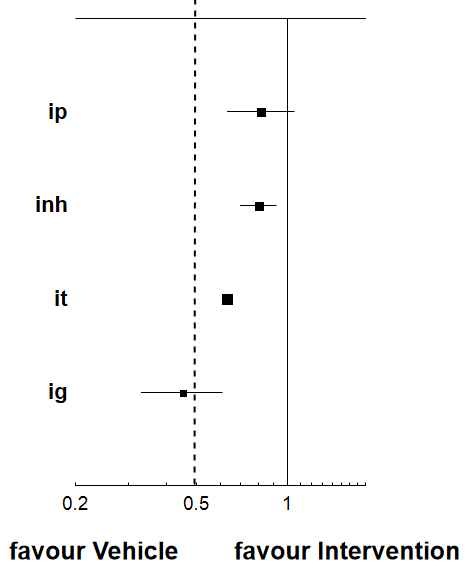


**c**

**d**

**e**

**Fig. 6.** Funnel plots showing the distribution of published study outcomes (filled squares) vs. unpublished outcomes (open circles) estimated by Trim and Fill analysis. Dashed line represents the mean and 95% CI with the added, potentially unpublished, studies and solid line represents published studies included into meta-analysis. Vertical dashed line represents the global estimate of efficacy. Overall effect size (D) for Vehicle 29.40 (28.23–30.58) *vs.* 25.35 (24.17–26.53) – 54 potentially missing studies were added (a); overall effect size (D) for Intervention 14.65 (13.73–15.57) *vs*. 10 (9.20–11.16) – 110 studies added (b); overall effect size (R) for Intervention group: 0.50 (95% CI 0.47–0.52) *vs*. 0.51 (95% CI 0.48–0.53) – 4 studies added (c); studies with exercise tests: effect size (D) for Vehicle: 612.3 (95% CI 484.1–740.6) *vs.* 461.5 (95% CI 333.8–589.1) – 13 studies added (d); effect size (D) for Intervention group: 152.28 (95% CI 85.72–218.84) – 0 studies added (e); studies with survival analysis: effect size (D) for Vehicle: 37.64 (33.97­41.32) *vs*. 37.04 (33.40­40.68) – one study added (f); effect size (R) for Intervention: 0.57 (0.50–0.65) *vs*. 0.77 (0.68–0.88) – 20 studies added (g); MCT subgroup: MCT subgroup: effect size (D) for Vehicle 31.84 (30.50–33.18) *vs*. 28.26 (26.89­29.64) – 34 studies added (h); effect size (R) for Intervention 0.50 (0.48–0.52) *vs*. 0.52 (0.50–0.55) – 8 studies added (i). According to visual inspection and results of Egger’s test there is a suggestion of missing studies and publication bias excluding e.


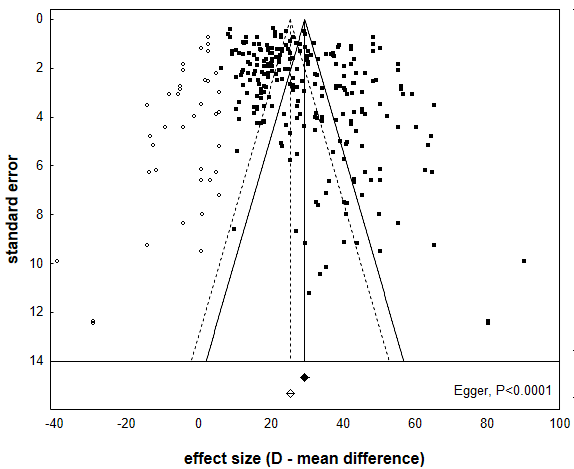

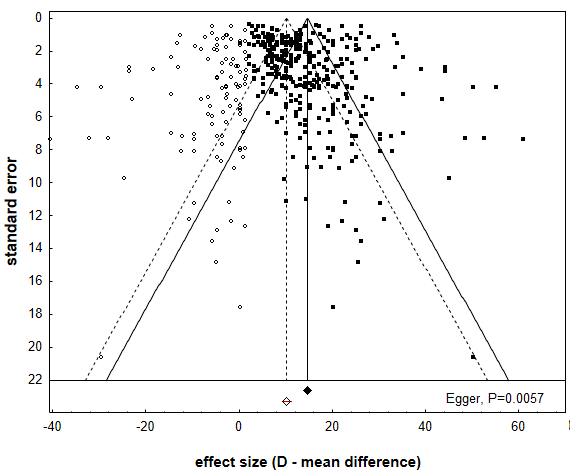

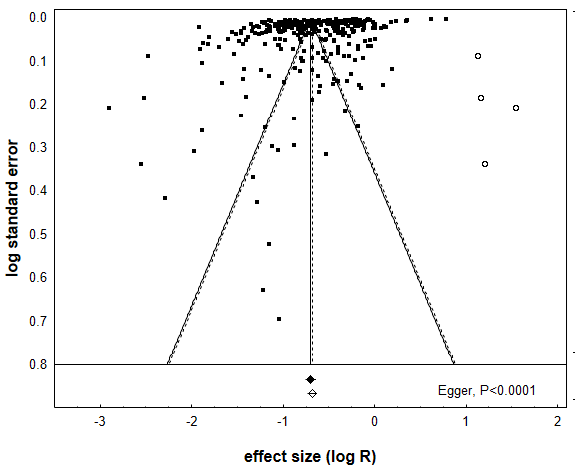


**a**

**b**

**c**


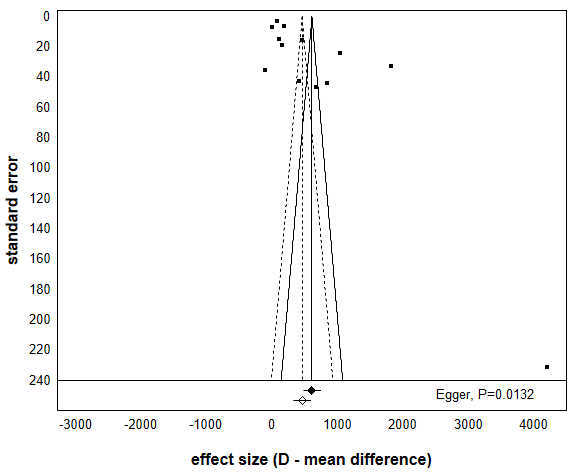

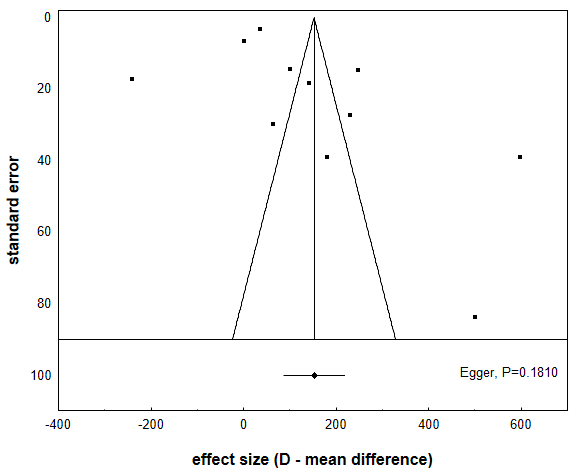

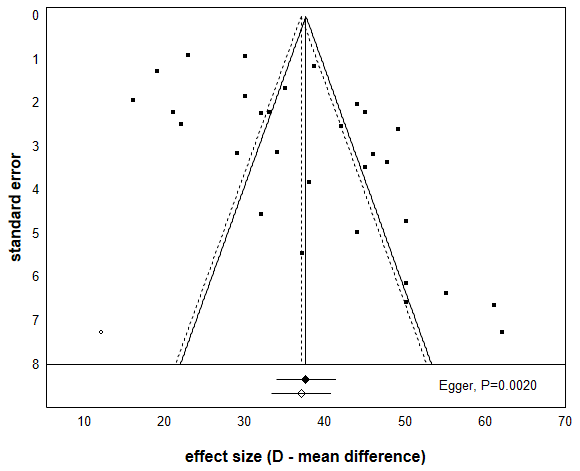


**d**

**e**

**f**


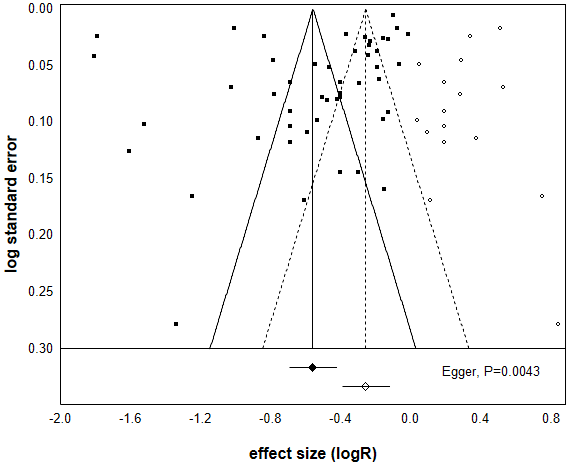

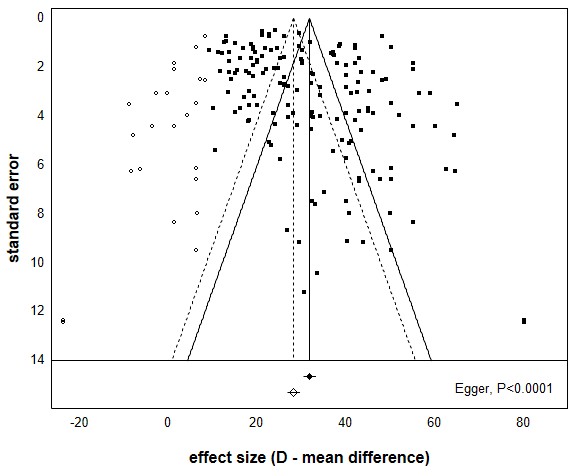

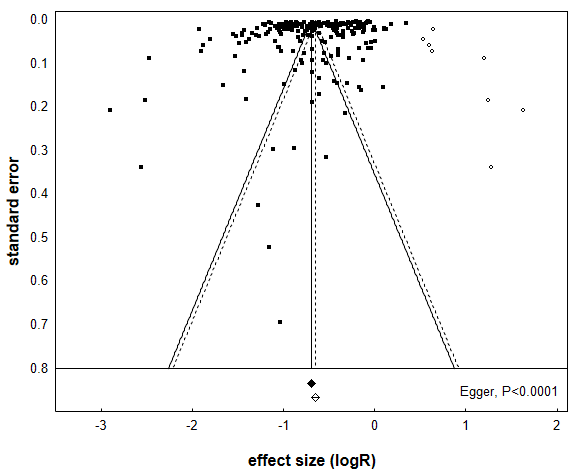


**g**

**h**

**i**

**Supplementary data. Study references.**

1. Abid S, Houssaini A, Chevarin C, Marcos E, Tissot CM, Gary-Bobo G, Wan F, Mouraret N, Amsellem V, Dubois-Randé JL, Hamon M, Adnot S. Inhibition of gut- and lung-derived serotonin attenuates pulmonary hypertension in mice. Am J Physiol Lung Cell Mol Physiol. 2012; 303: L500-8.
2. Agard C, Rolli-Derkinderen M, Dumas-de-La-Roque E, Rio M, Sagan C, Savineau JP, Loirand G, Pacaud P. Protective role of the antidiabetic drug metformin against chronic experimental pulmonary hypertension. Br J Pharmacol. 2009; 158: 1285-94.
3. Ahmed LA, Obaid AA, Zaki HF, Agha AM. Naringenin adds to the protective effect of L-arginine in monocrotaline-induced pulmonary hypertension in rats: favorable modulation of oxidative stress, inflammation and nitric oxide. Eur J Pharm Sci. 2014; 62: 161-70.
4. Ahmed LA, Obaid AA, Zaki HF, Agha AM. Role of oxidative stress, inflammation, nitric oxide and transforming growth factor-beta in the protective effect of diosgenin in monocrotaline-induced pulmonary hypertension in rats. Eur J Pharmacol. 2014; 740: 379-87.
5. Aiello RJ, Bourassa PA, Zhang Q, Dubins J, Goldberg DR, De Lombaert S, Humbert M, Guignabert C, Cavasin MA, McKinsey TA, Paralkar V. Tryptophan hydroxylase 1 Inhibition Impacts Pulmonary Vascular Remodeling in Two Rat Models of Pulmonary Hypertension. J Pharmacol Exp Ther. 2017; 360: 267-279.
6. Akagi S, Nakamura K, Miura D, Saito Y, Matsubara H, Ogawa A, Matoba T, Egashira K, Ito H. Delivery of imatinib-incorporated nanoparticles into lungs suppresses the development of monocrotaline-induced pulmonary arterial hypertension. Int Heart J. 2015; 56: 354-9.
7. Alencar AK, Carvalho FI, Silva AM, Martinez ST, Calasans-Maia JA, Fraga CM, Barreiro EJ, Zapata-Sudo G Sudo RT. Synergistic interaction between a PDE5 inhibitor (sildenafil) and a new adenosine A2A receptor agonist (LASSBio-1359) improves pulmonary hypertension in rats. PLoS One. 2018; 13: e0195047.
8. Alencar AK, Montes GC, Montagnoli T, Silva AM, Martinez ST, Fraga AG, Wang H, Groban L, Sudo RT, Zapata-Sudo G. Activation of GPER ameliorates experimental pulmonary hypertension in male rats.Eur J Pharm Sci. 2017; 97: 208-217.
9. Alencar AK, Pereira SL, da Silva FE, Mendes LV, Cunha Vdo M, Lima LM, Montagnoli TL, Caruso-Neves C, Ferraz EB, Tesch R, Nascimento JH, Sant'anna CM, Fraga CA, Barreiro EJ, Sudo RT, Zapata-Sudo G. N-acylhydrazone derivative ameliorates monocrotaline-induced pulmonary hypertension through the modulation of adenosine AA2R activity. Int J Cardiol. 2014; 173: 154-62.
10. Alencar AK, Pereira SL, Montagnoli TL, Maia RC, Kümmerle AE, Landgraf SS, Caruso-Neves C, Ferraz EB, Tesch R, Nascimento JH, de Sant'Anna CM, Fraga CA, Barreiro EJ, Sudo RT, Zapata-Sudo G. Beneficial effects of a novel agonist of the adenosine A2A receptor on monocrotaline-induced pulmonary hypertension in rats. Br J Pharmacol. 2013; 169: 953-62.
11. Alencar AKN, Montes GC, Costa DG, Mendes LVP, Silva AMS, Martinez ST, Trachez MM, Cunha VDMN, Montagnoli TL, Fraga AGM, Wang H, Groban L, Fraga CAM, Sudo RT, Zapata-Sudo G. Cardioprotection Induced by Activation of GPER in Ovariectomized Rats With Pulmonary Hypertension. J Gerontol A Biol Sci Med Sci. 2018; 73: 1158-1166.
12. Al-Hiti H, Chovanec M, Melenovský V, Vajnerová O, Baňasová A, Kautzner J, Herget J. L-arginine in combination with sildenafil potentiates the attenuation of hypoxic pulmonary hypertension in rats. Physiol Res. 2013; 62: 589-95.
13. Ambade AS, Jung B, Lee D, Doods H, Wu D. Triple-tyrosine kinase inhibition attenuates pulmonary arterial hypertension and neointimal formation. Transl Res. 2019; 203: 15-30.
14. Amirjanians M, Egemnazarov B, Sydykov A, Kojonazarov B, Brandes R, Luitel H, Pradhan K, Stasch JP, Redlich G, Weissmann N, Grimminger F, Seeger W, Ghofrani H, Schermuly R. Chronic intratracheal application of the soluble guanylyl cyclase stimulator BAY 41-8543 ameliorates experimental pulmonary hypertension. Oncotarget. 2017; 8: 29613-29624.
15. Andersen CU, Mulvany MJ, Simonsen U. Lack of synergistic effect of molsidomine and sildenafil on development of pulmonary hypertension in chronic hypoxic rats. Eur J Pharmacol. 2005; 510: 87-96.
16. Atlı Ö, Ilgın S, Ergun B, Burukoğlu D, Musmul A, Sırmagül B. Matrix metalloproteinases are possible targets in monocrotaline-induced pulmonary hypertension: investigation of anti-remodeling effects of alagebrium and everolimus. Anatol J Cardiol. 2017; 17: 8-17.
17. Bai Y, Li ZX Zhao YT, Liu M, Wang Y, Lian GC, Zhao Q, Wang HL. PCPA protects against monocrotaline-induced pulmonary arterial remodeling in rats: potential roles of connective tissue growth factor. Oncotarget. 2017; 8: 111642-111655.
18. Bai Y, Li ZX, Wang HL, Lian GC, Wang Y. The protective effects of PCPA against monocrotaline-induced pulmonary arterial hypertension are mediated through the downregulation of NFAT-1 and NF-κB. Int J Mol Med. 2017; 40: 155-163.
19. Bai Y, Wang HM, Liu M, Wang Y, Lian GC, Zhang XH, Kang J, Wang HL. 4-Chloro-DL-phenylalanine protects against monocrotaline-induced pulmonary vascular remodeling and lung inflammation. Int J Mol Med. 2014; 33: 373-82.
20. Baliga RS, Zhao L, Madhani M, Lopez-Torondel B, Visintin C, Selwood D, Wilkins MR, MacAllister RJ, Hobbs AJ. Synergy between natriuretic peptides and phosphodiesterase 5 inhibitors ameliorates pulmonary arterial hypertension. Am J Respir Crit Care Med. 2008; 178: 861-9.
21. Banasová A1, Maxová H, Hampl V, Vízek M, Povýsilová V, Novotná J, Vajnerová O, Hnilicková O, Herget J. Prevention of mast cell degranulation by disodium cromoglycate attenuates the development of hypoxic pulmonary hypertension in rats exposed to chronic hypoxia. Respiration. 2008; 76: 102-7.
22. Bauer EM, Zheng H, Lotze MT, Bauer PM. Recombinant human interferon alpha 2b prevents and reverses experimental pulmonary hypertension. PLoS One. 2014; 9:e96720.
23. Behringer A, Trappiel M, Berghausen EM, Ten Freyhaus H, Wellnhofer E, Odenthal M, Blaschke F, Er F, Gassanov N, Rosenkranz S, Baldus S, Kappert K, Caglayan E. Pioglitazone alleviates cardiac and vascular remodelling and improves survival in monocrotaline induced pulmonary arterial hypertension. Naunyn Schmiedebergs Arch Pharmacol. 2016; 389: 369-79.
24. Bhat L, Hawkinson J, Cantillon M, Reddy DG, Bhat SR, Laurent CE, Bouchard A, Biernat M, Salvail D. RP5063, a novel, multimodal, serotonin receptor modulator, prevents monocrotaline-induced pulmonary arterial hypertension in rats. Eur J Pharmacol. 2017; 810:92-99.
25. Bhat L, Hawkinson J, Cantillon M, Reddy DG, Bhat SR, Laurent CE, Bouchard A, Biernat M, Salvail D. Evaluation of the effects of RP5063, a novel, multimodal, serotonin receptor modulator, as single-agent therapy and co-administrated with sildenafil, bosentan, and treprostinil in a monocrotaline-induced pulmonary arterial hypertension rat model. Eur J Pharmacol. 2018; 827:159-166.
26. Bi LQ, Zhu R, Kong H, Wu SL, Li N, Zuo XR, Zhou SM, Kou JP, Yu BY, Wang H, Xie WP. Ruscogenin attenuates monocrotaline-induced pulmonary hypertension in rats. Int Immunopharmacol. 2013; 16: 7-16.
27. Blumberg FC, Wolf K, Sandner P, Lorenz C, Riegger GA, Pfeifer M. The NO donor molsidomine reduces endothelin-1 gene expression in chronic hypoxic rat lungs. Am J Physiol Lung Cell Mol Physiol. 2001; 280: L258-63.
28. Boehm M, Arnold N, Braithwaite A, Pickworth J, Lu C, Novoyatleva T, Kiely DG, Grimminger F, Ghofrani HA, Weissmann N, Seeger W, Lawrie A, Schermuly RT, Kojonazarov B. Eplerenone attenuates pathological pulmonary vascular rather than right ventricular remodeling in pulmonary arterial hypertension. BMC Pulm Med. 2018; 18: 41.
29. Bogaard HJ, Natarajan R, Mizuno S, Abbate A, Chang PJ, Chau VQ, Hoke NN, Kraskauskas D, Kasper M, Salloum FN, Voelkel NF. Adrenergic receptor blockade reverses right heart remodeling and dysfunction in pulmonary hypertensive rats. Am J Respir Crit Care Med. 2010; 182: 652-60.
30. Bombicz M, Priksz D, Varga B, Kurucz A, Kertész A, Takacs A, Posa A, Kiss R, Szilvassy Z, Juhasz B. A Novel Therapeutic Approach in the Treatment of Pulmonary Arterial Hypertension: Allium ursinum Liophylisate Alleviates Symptoms Comparably to Sildenafil. Int J Mol Sci. 2017; 18. pii: E1436.
31. Bonnet S, Dumas-de-La-Roque E, Bégueret H, Marthan R, Fayon M, Dos Santos P, Savineau JP, Baulieu EE. Dehydroepiandrosterone (DHEA) prevents and reverses chronic hypoxic pulmonary hypertension. Proc Natl Acad Sci U S A. 2003; 100: 9488-93.
32. Breitling S, Krauszman A, Parihar R, Walther T, Friedberg MK, Kuebler WM. Dose-dependent, therapeutic potential of angiotensin-(1-7) for the treatment of pulmonary arterial hypertension. Pulm Circ. 2015; 5: 649-57.
33. Bruce E, Shenoy V, Rathinasabapathy A, Espejo A, Horowitz A, Oswalt A, Francis J, Nair A, Unger T, Raizada MK, Steckelings UM, Sumners C, Katovich MJ. Selective activation of angiotensin AT2 receptors attenuates progression of pulmonary hypertension and inhibits cardiopulmonary fibrosis. Br J Pharmacol. 2015; 172: 2219-31.
34. Bubb KJ, Trinder SL, Baliga RS, Patel J, Clapp LH, MacAllister RJ, Hobbs AJ. Inhibition of phosphodiesterase 2 augments cGMP and cAMP signaling to ameliorate pulmonary hypertension. Circulation. 2014; 130: 496-507.
35. Campos C, de Castro AL, Tavares AM, Fernandes RO, Ortiz VD, Barboza TE, Pereira C, Apel M, da Silva OS, Llesuy S, Araujo AS, Belló-Klein A. Effect of Free and Nanoencapsulated Copaiba Oil on Monocrotaline-induced Pulmonary Arterial Hypertension. J Cardiovasc Pharmacol. 2017; 69: 79-85.
36. Campos-Carraro C, Turck P, de Lima-Seolin BG, Tavares AMV, Dos Santos Lacerda D, Corssac GB, Teixeira RB, Hickmann A, Llesuy S, da Rosa Araujo AS, Belló-Klein A. Copaiba Oil Attenuates Right Ventricular Remodeling by Decreasing Myocardial Apoptotic Signaling in Monocrotaline-Induced Rats. J Cardiovasc Pharmacol. 2018; 72: 214-221.
37. Cha SA, Park BM, Kim SH. Angiotensin-(1-9) ameliorates pulmonary arterial hypertension via angiotensin type II receptor. Korean J Physiol Pharmacol. 2018; 22: 447-456.
38. Chabert C, Khochbin S, Rousseaux S, Veyrenc S, Furze R, Smithers N, Prinjha RK, Schlattner U, Pison C, Dubouchaud H. Inhibition of BET Proteins Reduces Right Ventricle Hypertrophy and Pulmonary Hypertension Resulting from Combined Hypoxia and Pulmonary Inflammation. Int J Mol Sci. 2018; 19. pii: E2224.
39. Chan MC, Weisman AS, Kang H, Nguyen PH, Hickman T, Mecker SV, Hill NS, Lagna G, Hata A. The amiloride derivative phenamil attenuates pulmonary vascular remodeling by activating NFAT and the bone morphogenetic protein signaling pathway. Mol Cell Biol. 2011; 31: 517-30.
40. Chang H, Chang CY, Lee HJ, Chou CY, Chou TC. Magnolol ameliorates pneumonectomy and monocrotaline-induced pulmonary arterial hypertension in rats through inhibition of angiotensin II and endothelin-1 expression. Phytomedicine. 2018; 51: 205-213.
41. Chang LT, Sun CK, Sheu JJ, Chiang CH, Youssef AA, Lee FY, Wu CJ, Yip HK. Cilostazol therapy attenuates monocrotaline-induced pulmonary arterial hypertension in rat model. Circ J. 2008; 72: 825-31.
42. Chaumais MC, Ranchoux B, Montani D, Dorfmüller P, Tu L, Lecerf F, Raymond N, Guignabert C, Price L, Simonneau G, Cohen-Kaminsky S, Humbert M, Perros F. N-acetylcysteine improves established monocrotaline-induced pulmonary hypertension in rats. Respir Res. 2014; 15: 65.
43. Chen F, Wang H, Lai J, Cai S, Yuan L. 3-Bromopyruvate reverses hypoxia-induced pulmonary arterial hypertension through inhibiting glycolysis: In vitro and in vivo studies. Int J Cardiol. 2018; 266: 236-241.
44. Chen F, Wang H, Yan J, Lai J, Cai S, Yuan L, Zheng. Grape seed proanthocyanidin reverses pulmonary vascular remodeling in monocrotaline-induced pulmonary arterial hypertension by down-regulating HSP70. Biomed Pharmacother. 2018; 101: 123-128.
45. Chen L, Xiao J, Li Y, Ma H. Ang-(1-7) might prevent the development of monocrotaline induced pulmonary arterial hypertension in rats. Eur Rev Med Pharmacol Sci. 2011; 15: 1-7.
46. Chen R, Jiang P, Liu LY, Gao Y, Yang XX, Cai ZC, Fan XF, Gong YS, Mao SZ. [Apelin attenuates hypoxia induced pulmonary hypertension of mice through regulation of lipid metabolism]. Zhongguo Ying Yong Sheng Li Xue Za Zhi. 2017; 33: 493-496.
47. Chen R, Zhong W, Shao C, Liu P, Wang C, Wang Z, Jiang M, Lu Y, Yan J. Docosahexaenoic acid inhibits monocrotaline-induced pulmonary hypertension via attenuating endoplasmic reticulum stress and inflammation. Am J Physiol Lung Cell Mol Physiol. 2018; 314: L243-L255.
48. Chen WS, Li XQ, Cao W, Xiao X, Dong L, Zhang JZ. Vardenafil ameliorates calcium mobilization in pulmonary artery smooth muscle cells from hypoxic pulmonary hypertensive mice. Arch Med Res. 2012; 43: 265-73.
49. Chen X, Cheng D, Guan J, Xia X, Fan L, Su Q. Preventive effects of montelukast on hypoxic pulmonary hypertension in rats. Sichuan Da Xue Xue Bao Yi Xue Ban. 2003; 34: 555-8.
50. Chen XY, Zhang W, Miao QF, Zhang YJ. m-Nisoldipine attenuates monocrotaline-induced pulmonary hypertension by suppressing 5-HT/ERK MAPK pathway. Yao Xue Xue Bao. 2008; 43: 1011-8.
51. Chen YC, Yuan TY, Zhang HF, Wang DS1, Yan Y, Niu ZR, Lin YH, Fang LH, Du GH. Salvianolic acid A attenuates vascular remodeling in a pulmonary arterial hypertension rat model. Acta Pharmacol Sin. 2016; 37: 772–782.
52. Cheng D, Chen W. Effects of ginkgolide B on isobaric hypoxic pulmonary hypertension in rats. Chin Med J (Engl). 1996; 109: 881-4.
53. Cheng Y, Gong Y, Qian S, Mou Y, Li H, Chen X, Kong H, Xie W, Wang H, Zhang Y, Huang Z. Identification of a Novel Hybridization from Isosorbide 5-Mononitrate and Bardoxolone Methyl with Dual Activities of Pulmonary Vasodilation and Vascular Remodeling Inhibition on Pulmonary Arterial Hypertension Rats. J Med Chem. 2018; 61: 1474-1482.
54. Chung HH, Dai ZK, Wu BN, Yeh JL, Chai CY, Chu KS, Liu CP, Chen IJ. The xanthine derivative KMUP-1 inhibits models of pulmonary artery hypertension via increased NO and cGMP-dependent inhibition of RhoA/Rho kinase. Br J Pharmacol. 2010; 160: 971-86.
55. Church AC, Martin DH, Wadsworth R, Bryson G, Fisher AJ, Welsh DJ, Peacock AJ. The reversal of pulmonary vascular remodeling through inhibition of p38 MAPK-alpha: a potential novel anti-inflammatory strategy in pulmonary hypertension. Am J Physiol Lung Cell Mol Physiol. 2015; 309: L333-47.
56. Courboulin A, Barrier M, Perreault T, Bonnet P, Tremblay VL, Paulin R, Tremblay E, Lambert C, Jacob MH, Bonnet SN, Provencher S, Bonnet S. Plumbagin reverses proliferation and resistance to apoptosis in experimental PAH. Eur Respir J. 2012; 40: 618-29.
57. Cowan KN, Heilbut A, Humpl T, Lam C, Ito S, Rabinovitch M. Complete reversal of fatal pulmonary hypertension in rats by a serine elastase inhibitor. Nat Med. 2000; 6: 698-702.
58. Crossno JT Jr, Garat CV, Reusch JE, Morris KG, Dempsey EC, McMurtry IF, Stenmark KR, Klemm DJ. Rosiglitazone attenuates hypoxia-induced pulmonary arterial remodeling. Am J Physiol Lung Cell Mol Physiol. 2007; 292: L885-97.
59. Csiszar A, Labinskyy N, Olson S, Pinto JT, Gupte S, Wu JM, Hu F, Ballabh P, Podlutsky A, Losonczy G, de Cabo R, Mathew R, Wolin MS, Ungvari Z. Resveratrol prevents monocrotaline-induced pulmonary hypertension in rats. Hypertension. 2009; 54: 668-75.
60. Dahal BK, Kosanovic D, Pamarthi PK, Sydykov A, Lai YJ, Kast R, Schirok H, Stasch JP, Ghofrani HA, Weissmann N, Grimminger F, Seeger W, Schermuly RT. Therapeutic efficacy of azaindole-1 in experimental pulmonary hypertension. Eur Respir J. 2010; 36: 808-18.
61. Dai G, Li B, Xu Y, Zeng Z, Yang H. Oxymatrine prevents the development of monocrotaline-induced pulmonary hypertension via regulation of the NG, NG-dimethyl-L-arginine metabolism pathways in rats. Eur J Pharmacol. 2019; 842: 338-344.
62. Dai L, Wu S. Atorvastatin attenuates hypoxic pulmonary hypertension in rats by inhibiting RhoA/Rho kinase pathway. Zhong Nan Da Xue Xue Bao Yi Xue Ban. 2011; 36: 58-63.
63. Dai ZK, Cheng YJ, Chung HH, Wu JR, Chen IJ, Wu BN. KMUP-1 ameliorates monocrotaline-induced pulmonary arterial hypertension through the modulation of Ca2+ sensitization and K+-channel. Life Sci. 2010; 86: 747-55.
64. de Lima-Seolin BG, Hennemann MM, Fernandes RO, Colombo R, Bonetto JHP, Teixeira RB, Khaper N, Godoy AEG, Litvin IE, Sander da Rosa Araujo A, Schenkel PC, Belló-Klein A. Bucindolol attenuates the vascular remodeling of pulmonary arteries by modulating the expression of the endothelin-1 A receptor in rats with pulmonary arterial hypertension. Biomed Pharmacother. 2018; 99: 704-714.
65. de Man FS, Handoko ML, van Ballegoij JJ, Schalij I, Bogaards SJ, Postmus PE, van der Velden J, Westerhof N, Paulus WJ, Vonk-Noordegraaf A. Bisoprolol Delays Progression Towards Right Heart Failure in Experimental Pulmonary Hypertension. Circ Heart Fail. 2012; 5: 97-105
66. De Raaf MA, Hussaini AA, Gomez-Arroyo J, Kraskaukas D, Farkas D, Happé C, Voelkel NF, Bogaard HJ. Histone deacetylase inhibition with trichostatin A does not reverse severe angioproliferative pulmonary hypertension in rats. Pulm Circ. 2014; 4: 237-43.
67. Dean A, Nilsen M, Loughlin L, Salt IP, MacLean MR. Metformin Reverses Development of Pulmonary Hypertension via Aromatase Inhibition. Hypertension. 2016; 68: 446-54.
68. Delbeck M, Nickel KF, Perzborn E, Ellinghaus P, Strassburger J, Kast R, Laux V, Schäfer S, Schermuly RT, von Degenfeld G. A role for coagulation factor Xa in experimental pulmonary arterial hypertension. Cardiovasc Res. 2011; 92: 159-68.
69. Dumitrascu R, Kulcke C, Königshoff M, Kouri F, Yang X, Morrell N, Ghofrani HA, Weissmann N, Reiter R, Seeger W, Grimminger F, Eickelberg O, Schermuly RT, Pullamsetti SS. Terguride ameliorates monocrotaline-induced pulmonary hypertension in rats. Eur Respir J. 2011; 37: 1104-18.
70. Eba S, Hoshikawa Y, Moriguchi T, Mitsuishi Y, Satoh H, Ishida K, Watanabe T, Shimizu T, Shimokawa H, Okada Y, Yamamoto M, Kondo T. The nuclear factor erythroid 2-related factor 2 activator oltipraz attenuates chronic hypoxia-induced cardiopulmonary alterations in mice. Am J Respir Cell Mol Biol. 2013; 49: 324-33.
71. Elias-Al-Mamun M, Satoh K, Tanaka S, Shimizu T, Nergui S, Miyata S, Fukumoto Y, Shimokawa H. Combination therapy with fasudil and sildenafil ameliorates monocrotaline-induced pulmonary hypertension and survival in rats. Circ J. 2014; 78: 967-76.
72. Elmedal B, de Dam MY, Mulvany MJ, Simonsen U. The superoxide dismutase mimetic, tempol, blunts right ventricular hypertrophy in chronic hypoxic rats. Br J Pharmacol. 2004; 141: 105-13.
73. Fan Z, Liu B, Zhang S, Liu H, Li Y, Wang D, Liu Y, Li J, Wang N, Liu Y, Zhang B. YM155, a selective survivin inhibitor, reverses chronic hypoxic pulmonary hypertension in rats via upregulating voltage-gated potassium channels. Clin Exp Hypertens. 2015; 37: 381-7.
74. Farhat MY, Chen MF, Bhatti T, Iqbal A, Cathapermal S, Ramwell PW. Protection by oestradiol against the development of cardiovascular changes associated with monocrotaline pulmonary hypertension in rats. Br J Pharmacol. 1993; 110: 719-23.
75. Faul JL, Nishimura T, Berry GJ, Benson GV, Pearl RG, Kao PN. Triptolide attenuates pulmonary arterial hypertension and neointimal formation in rats. Am J Respir Crit Care Med. 2000; 162: 2252-8.
76. Feng Z, Hu Y, An NN, Feng WJ, Hu T, Mao YJ. Effect of acidic oligosaccharides on P-selectin of pulmonary hypertensive rats induced by monocrotaline]. Zhonghua Jie He He Hu Xi Za Zhi. 2018; 41: 90-94.
77. Ferreira AJ, Shenoy V, Yamazato Y, Sriramula S, Francis J, Yuan L, Castellano RK, Ostrov DA, Oh SP, Katovich MJ, Raizada MK. Evidence for angiotensin-converting enzyme 2 as a therapeutic target for the prevention of pulmonary hypertension. Am J Respir Crit Care Med. 2009; 179: 1048-54.
78. Fowler ED, Drinkhill MJ, Norman R, Pervolaraki E, Stones R, Steer E, Benoist D, Steele DS, Calaghan SC, White E. Beta1-adrenoceptor antagonist, metoprolol attenuates cardiac myocyte Ca2+ handling dysfunction in rats with pulmonary artery hypertension. J Mol Cell Cardiol. 2018; 120: 74-83.
79. Fowler ED, Drinkhill MJ, Stones R, White E. Diastolic dysfunction in pulmonary artery hypertension: Creatine kinase and the potential therapeutic benefit of beta-blockers. Clin Exp Pharmacol Physiol. 2018; 45: 384-389.
80. Francis BN, Salameh M, Khamisy-Farah R, Farah R. Tetrahydrobiopterin (BH4 ): Targeting endothelial nitric oxide synthase as a potential therapy for pulmonary hypertension. Cardiovasc Ther. 2018; 36. Epub 2017 Dec 3.
81. Frump AL, Albrecht ME, McClintick JN, Lahm T. Estrogen receptor-dependent attenuation of hypoxia-induced changes in the lung genome of pulmonary hypertension rats. Pulm Circ. 2017; 7: 232-243.
82. Frump AL, Goss KN, Vayl A, Albrecht M, Fisher A, Tursunova R, Fierst J, Whitson J, Cucci AR, Brown MB, Lahm T. Estradiol improves right ventricular function in rats with severe angioproliferative pulmonary hypertension: effects of endogenous and exogenous sex hormones. Am J Physiol Lung Cell Mol Physiol. 2015; 308: L873-90.
83. Gao H, Chen C, Huang S, Li B. Quercetin attenuates the progression of monocrotaline-induced pulmonary hypertension in rats. J Biomed Res. 2012; 26: 98-102.
84. Gao H, Cheng Y, Zong L, Huang L, Qiao C, Li W, Gong B, Hu J, Liu H, Wang X, Zhao C. Aspirin attenuates monocrotaline-induced pulmonary arterial hypertension in rats by suppressing the ERK/MAPK pathway. Clin Exp Hypertens. 2017; 39: 34-41.
85. Garat CV, Crossno JT Jr, Sullivan TM, Reusch JE, Klemm DJ. Inhibition of phosphatidylinositol 3-kinase/Akt signaling attenuates hypoxia-induced pulmonary artery remodeling and suppresses CREB depletion in arterial smooth muscle cells. J Cardiovasc Pharmacol. 2013; 62: 539-48.
86. Gary-Bobo G, Houssaini A, Amsellem V, Rideau D, Pacaud P, Perrin A, Brégeon J, Marcos E, Dubois-Randé JL, Sitbon O, Savale L, Adnot S. Effects of HIV protease inhibitors on progression of monocrotaline- and hypoxia-induced pulmonary hypertension in rats. Circulation. 2010; 122: 1937-47.
87. Geng J, Fan FL, He S, Liu Y, Meng Y, Tian H, Zhang D, Ma Q, Zhang JB, Tian HY. The effects of the 5-HT2A receptor antagonist sarpogrelate hydrochloride on chronic hypoxic pulmonary hypertension in rats. Exp Lung Res. 2016;42: 190-8.
88. Girgis RE, Li D, Zhan X, Garcia JG, Tuder RM, Hassoun PM, Johns RA. Attenuation of chronic hypoxic pulmonary hypertension by simvastatin. Am J Physiol Heart Circ Physiol. 2003; 285: H938-45.
89. Girgis RE, Mozammel S, Champion HC, Li D, Peng X, Shimoda L, Tuder RM, Johns RA, Hassoun PM. Regression of chronic hypoxic pulmonary hypertension by simvastatin. Am J Physiol Lung Cell Mol Physiol. 2007; 292: L1105-10.
90. Green DE, Murphy TC, Kang BY, Kleinhenz JM, Szyndralewiez C, Page P, Sutliff RL, Hart CM. The Nox4 inhibitor GKT137831 attenuates hypoxia-induced pulmonary vascular cell proliferation. Am J Respir Cell Mol Biol. 2012; 47: 718-26.
91. Grzegorzewska AP, Seta F, Han R, Czajka CA, Makino K, Stawski L, Isenberg J, Browning JL, Trojanowska M. Dimethyl Fumarate ameliorates pulmonary arterial hypertension and lung fibrosis by targeting multiple pathways. Sci Rep. 2017; 7: 41605.
92. Guerard P, Rakotoniaina Z, Goirand F, Rochette L, Dumas M, Lirussi F, Bardou M. The HMG-CoA reductase inhibitor, pravastatin, prevents the development of monocrotaline-induced pulmonary hypertension in the rat through reduction of endothelial cell apoptosis and overexpression of eNOS. Naunyn Schmiedebergs Arch Pharmacol. 2006; 373: 401-14.
93. Guignabert C, Raffestin B, Benferhat R, Raoul W, Zadigue P, Rideau D, Hamon M, Adnot S, Eddahibi S. Serotonin transporter inhibition prevents and reverses monocrotaline-induced pulmonary hypertension in rats. Circulation. 2005; 111: 2812-9.
94. Guo S, Shen Y, He G, Wang T, Xu D, Wen F. Involvement of Ca2+-activated K+ channel 3.1 in hypoxia-induced pulmonary arterial hypertension and therapeutic effects of TRAM-34 in rats. Biosci Rep. 2017; 37. pii: BSR20170763.
95. Gupta N, Rashid J, Nozik-Grayck E, McMurtry IF, Stenmark KR, Ahsan F. Cocktail of Superoxide Dismutase and Fasudil Encapsulated in Targeted Liposomes Slows PAH Progression at a Reduced Dosing Frequency. Mol Pharm. 2017; 14: 830-841.
96. Hamidi SA, Lin RZ, Szema AM, Lyubsky S, Jiang YP, Said SI. VIP and endothelin receptor antagonist: an effective combination against experimental pulmonary arterial hypertension. Respir Res. 2011; 12: 141.
97. Hampl V, Bíbová J, Povýsilová V, Herget J. Dehydroepiandrosterone sulphate reduces chronic hypoxic pulmonary hypertension in rats. Eur Respir J. 2003; 21: 862-5.
98. Hansen MS, Andersen A, Holmboe S, Schultz JG, Ringgaard S, Simonsen U, Happé C, Bogaard HJ, Nielsen-Kudsk JE. Levosimendan Prevents and Reverts Right Ventricular Failure in Experimental Pulmonary Arterial Hypertension. J Cardiovasc Pharmacol. 2017; 70: 232-238.
99. Hara Y, Sassi Y, Guibert C, Gambaryan N, Dorfmüller P, Eddahibi S, Lompré AM, Humbert M, Hulot JS. Inhibition of MRP4 prevents and reverses pulmonary hypertension in mice. J Clin Invest. 2011; 121: 2888-97.
100. Harrington LS, Moreno L, Reed A, Wort SJ, Desvergne B, Garland C, Zhao L, Mitchell JA. The PPARbeta/delta agonist GW0742 relaxes pulmonary vessels and limits right heart hypertrophy in rats with hypoxia-induced pulmonary hypertension. PLoS One. 2010; 5.
101. He Q, Nan X, Li S, Su S, Ma K, Li Z, Lu D, Ge R. Tsantan Sumtang Alleviates Chronic Hypoxia-Induced Pulmonary Hypertension by Inhibiting Proliferation of Pulmonary Vascular Cells. Biomed Res Int. 2018; 2018: 9504158.
102. He Y, Cao X, Liu X, Li X, Xu Y, Liu J, Shi J. Quercetin reverses experimental pulmonary arterial hypertension by modulating the TrkA pathway. Exp Cell Res. 2015; 339: 122-34.
103. He YY, Liu CL, Li X, Li RJ, Wang LL, He KL. Salubrinal attenuates right ventricular hypertrophy and dysfunction in hypoxic pulmonary hypertension of rats. Vascul Pharmacol. 2016; 87: 190-198.
104. Henriques-Coelho T, Roncon-Albuquerque Júnior R, Lourenço AP, Baptista MJ, Oliveira SM, Brandao-Nogueira A, Correia-Pinto J, Leite-Moreira AF. Ghrelin reverses molecular, structural and hemodynamic alterations of the right ventricle in pulmonary hypertension. Rev Port Cardiol. 2006; 25: 55-63.
105. Herget J, Novotna J, Bibova J, Povysilova V, Vankova M, Hampl V. Metalloproteinase inhibition by Batimastat attenuates pulmonary hypertension in chronically hypoxic rats. Am J Physiol Lung Cell Mol Physiol. 2003; 285: L199-208.
106. Himori K, Abe M, Tatebayashi D, Lee J, Westerblad H, Lanner JT, Yamada T. Superoxide dismutase/catalase mimetic EUK-134 prevents diaphragm muscle weakness in monocrotalin-induced pulmonary hypertension. PLoS One. 2017; 12: e0169146.
107. Hironaka E, Hongo M, Sakai A, Mawatari E, Terasawa F, Okumura N, Yamazaki A, Ushiyama Y, Yazaki Y, Kinoshita O. Serotonin receptor antagonist inhibits monocrotaline-induced pulmonary hypertension and prolongs survival in rats. Respiration. 2006; 73: 105-12.
108. Honda J, Kimura T, Sakai S, Maruyama H, Tajiri K, Murakoshi N, Homma S, Miyauchi T, Aonuma K. The glucagon-like peptide-1 receptor agonist liraglutide improves hypoxia-induced pulmonary hypertension in mice partly via normalization of reduced ET(B) receptor expression. Physiol Res. 2018; 67(Supplementum 1): S175-S184.
109. Hongo M, Mawatari E, Sakai A, Ruan Z, Koizumi T, Terasawa F, Yazaki Y, Kinoshita O, Ikeda U, Shibamoto T. Effects of nicorandil on monocrotaline-induced pulmonary arterial hypertension in rats. J Cardiovasc Pharmacol. 2005; 46: 452-8.
110. Hosokawa S1, Haraguchi G, Sasaki A, Arai H, Muto S, Itai A, Doi S, Mizutani S, Isobe M. Pathophysiological roles of nuclear factor kappaB (NF-kB) in pulmonary arterial hypertension: effects of synthetic selective NF-kB inhibitor IMD-0354. Cardiovasc Res. 2013; 99: 35-43.
111. Houssaini A, Abid S, Mouraret N, Wan F, Rideau D, Saker M, Marcos E, Tissot CM, Dubois-Randé JL, Amsellem V, Adnot S. Rapamycin reverses pulmonary artery smooth muscle cell proliferation in pulmonary hypertension. Am J Respir Cell Mol Biol. 2013; 48: 568–577.
112. Hsu WL, Lin YC, Jeng JR, Chang HY, Chou TC. Baicalein Ameliorates Pulmonary Arterial Hypertension Caused by Monocrotaline through Downregulation of ET-1 and ETAR in Pneumonectomized Rats. Am J Chin Med. 2018; 46: 769-783.
113. Hu H, Zharikov S, Patel JM. Novel peptide for attenuation of hypoxia-induced pulmonary hypertension via modulation of nitric oxide release and phosphodiesterase -5 activity. Peptides. 2012; 35: 78-85.
114. Hu Y, Feng Z, Feng W, Hu T, Guan H, Mao Y. AOS ameliorates monocrotaline-induced pulmonary hypertension by restraining the activation of P-selectin/p38MAPK/NF-κB pathway in rats. Biomed Pharmacother. 2019; 109: 1319-1326.
115. Hua C, Zhao J, Wang H, Chen F, Meng H, Chen L, Zhang Q, Yan J, Yuan L. Apple polyphenol relieves hypoxia-induced pulmonary arterial hypertension via pulmonary endothelium protection and smooth muscle relaxation: In vivo and in vitro studies. Biomed Pharmacother. 2018; 107: 937-944.
116. Huang X, Fan R, Lu Y, Yu C, Xu X, Zhang X, Liu P, Yan S, Chen C, Wang L. Regulatory effect of AMP-activated protein kinase on pulmonary hypertension induced by chronic hypoxia in rats: in vivo and in vitro studies. Mol Biol Rep. 2014; 41: 4031-41.
117. Huang X, Zou L, Yu X, Chen M, Guo R, Cai H, Yao D, Xu X, Chen Y, Ding C, Cai X, Wang L. Salidroside attenuates chronic hypoxia-induced pulmonary hypertension via adenosine A2a receptor related mitochondria-dependent apoptosis pathway. J Mol Cell Cardiol. 2015; 82: 153-66.
118. Huang YF, Liu ML, Dong MQ, Yang WC, Zhang B, Luan LL, Dong HY, Xu M, Wang YX, Liu LL, Gao YQ, Li ZC. Effects of sodium tanshinone II A sulphonate on hypoxic pulmonary hypertension in rats in vivo and on Kv2.1 expression in pulmonary artery smooth muscle cells in vitro. J Ethnopharmacol. 2009; 125: 436-43.
119. Huang Z, Liu Z, Luo Q, Zhao Z, Zhao Q, Zheng Y, Xi Q, Tang Y. Glycoprotein 130 Inhibitor Ameliorates Monocrotaline-Induced Pulmonary Hypertension in Rats. Can J Cardiol. 2016; 32: 1356.e1-1356.
120. Huh JW, Kim SY, Lee JH, Lee YS. YC-1 attenuates hypoxia-induced pulmonary arterial hypertension in mice. Pulm Pharmacol Ther. 2011; 24: 638-46.
121. Hung MW, Yeung HM, Lau CF, Poon AMS, Tipoe GL, Fung ML. Melatonin Attenuates Pulmonary Hypertension in Chronically Hypoxic Rats. Int J Mol Sci. 2017; 18. pii: E1125.
122. Ichimura K, Matoba T, Koga JI, Nakano K, Funamoto D, Tsutsui H, Egashira K. Nanoparticle-Mediated Targeting of Pitavastatin to Small Pulmonary Arteries and Leukocytes by Intravenous Administration Attenuates the Progression of Monocrotaline-Induced Established Pulmonary Arterial Hypertension in Rats. Int Heart J. 2018; 59: 1432-1444.
123. Inoue H, Yano K, Noto T, Takagi M, Ikeo T, Kikkawa K. Acute and chronic effects of T-1032, a novel selective phosphodiesterase type 5 inhibitor, on monocrotaline-induced pulmonary hypertension in rats. Biol Pharm Bull. 2002; 25: 1422-6.
124. Ishikawa K, Hashimoto H, Mitani S, Toki Y, Okumura K, Ito T. Enalapril improves heart failure induced by monocrotaline without reducing pulmonary hypertension in rats: roles of preserved myocardial creatine kinase and lactate dehydrogenase isoenzymes. Int J Cardiol. 1995; 47: 225-33.
125. Jasińska-Stroschein M Owczarek J Plichta P. Orszulak-Michalak D. Concurrent Rho-Kinase and Tyrosine Kinase Platelet-Derived Growth Factor Inhibition in Experimental Pulmonary Hypertension. Pharmacology 2014; 93: 145–150.
126. Jasińska-Stroschein M, Owczarek J, Cicha K, Orszulak-Michalak D. Influence of imatinib at a low dose and sildenafil on pulmonary hypertension in rats. Pharmazie. 2015; 70: 477-9.
127. Jasińska-Stroschein M, Owczarek J, Łuczak A, Orszulak-Michalak D. The beneficial impact of fasudil and sildenafil on monocrotaline-induced pulmonary hypertension in rats: a hemodynamic and biochemical study. Pharmacology. 2013; 91: 178-84.
128. Jasińska-Stroschein M, Owczarek J, Surowiecka A, Kącikowska J, Orszulak-Michalak D. HMG-COA reductase inhibitors: An opportunity for the improvement of imatinib safety. An experimental study in rat pulmonary hypertension. Pharmacol Rep. 2015; 67: 32-7.
129. Jasińska-Stroschein M, Owczarek J, Wesołowska A, Orszulak-Michalak D. Rosuvastatin, sildenafil and their combination in monocrotaline-induced pulmonary hypertension in rat. Acta Pharm. 2014; 64: 345-53.
130. Jeffery TK, Wanstall JC. Perindopril, an angiotensin converting enzyme inhibitor, in pulmonary hypertensive rats: comparative effects on pulmonary vascular structure and function. Br J Pharmacol. 1999; 128: 1407-18.
131. Jeffery TK, Wanstall JC. Pulmonary vascular remodelling in hypoxic rats: effects of amlodipine, alone and with perindopril. Eur J Pharmacol. 2001; 416: 123-31.
132. Jiang H, Guan RJ, Wang HY. Fasudil reverses monocrotaline-induced pulmonary hypertension in rats. Zhonghua Xin Xue Guan Bing Za Zhi. 2013; 41: 239-44.
133. Jiang L, Zhou T, Liu H. Combined effects of the ATP-sensitive potassium channel opener pinacidil and simvastatin on pulmonary vascular remodeling in rats with monocrotaline-induced pulmonary arterial hypertension. Pharmazie. 2012; 67: 547-52.
134. Jiang Y, Zhou Y, Peng G, Liu N, Tian H, Pan D, Liu L, Yang X, Li C, Li W, Chen L, Ran P, Dai A. Topotecan prevents hypoxia-induced pulmonary arterial hypertension and inhibits hypoxia-inducible factor-1α and TRPC channels. Int J Biochem Cell Biol. 2018; 104: 161-170.
135. Jin H, Liu M, Zhang X, Pan J, Han J, Wang Y, Lei H, Ding Y, Yuan Y. Grape seed procyanidin extract attenuates hypoxic pulmonary hypertension by inhibiting oxidative stress and pulmonary arterial smooth muscle cells proliferation. J Nutr Biochem. 2016; 36:81-88.
136. Jin H, Wang Y, Zhou L, Liu L, Zhang P, Deng W, Yuan Y. Melatonin attenuates hypoxic pulmonary hypertension by inhibiting the inflammation and the proliferation of pulmonary arterial smooth muscle cells. J Pineal Res. 2014; 57: 442-50.
137. Kameshima S, Kazama K, Okada M, Yamawaki H. Eukaryotic elongation factor 2 kinase mediates monocrotaline-induced pulmonary arterial hypertension via reactive oxygen species-dependent vascular remodeling. Am J Physiol Heart Circ Physiol. 2015; 308: H1298-305.
138. Kanai Y, Hori S, Tanaka T, Yasuoka M, Watanabe K, Aikawa N, Hosoda Y. Role of 5-hydroxytryptamine in the progression of monocrotaline induced pulmonary hypertension in rats. Cardiovasc Res. 1993; 27: 1619-23.
139. Kanno S, Wu YJ, Lee PC, Billiar TR, Ho C. Angiotensin-converting enzyme inhibitor preserves p21 and endothelial nitric oxide synthase expression in monocrotaline-induced pulmonary arterial hypertension in rats. Circulation. 2001; 104: 945-50.
140. Kataoka M, Nagaya N, Satoh T, Itoh T, Murakami S, Iwase T, Miyahara Y, Kyotani S, Sakai Y, Kangawa K, Ogawa S. A long-acting prostacyclin agonist with thromboxane inhibitory activity for pulmonary hypertension. Am J Respir Crit Care Med. 2005; 172: 1575-80.
141. Kato T, Nasu T, Sonoda H, Ito KM, Ikeda M, Ito K. Evaluation of olmesartan medoxomil in the rat monocrotaline model of pulmonary hypertension. J Cardiovasc Pharmacol. 2008; 51: 18-23.
142. Kazama K, Okada M, Yamawaki H. A novel adipocytokine, omentin, inhibits monocrotaline-induced pulmonary arterial hypertension in rats. Biochem Biophys Res Commun. 2014; 452: 142-6.
143. Kim EK, Lee JH, Oh YM, Lee YS, Lee SD. Rosiglitazone attenuates hypoxia-induced pulmonary arterial hypertension in rats. Respirology. 2010; 15: 659-68.
144. Kim SY, Lee JH, Huh JW, Kim HJ, Park MK, Ro JY, Oh YM, Lee SD, Lee YS. Bortezomib alleviates experimental pulmonary arterial hypertension. Am J Respir Cell Mol Biol. 2012; 47: 698-708.
145. Klein M, Schermuly RT, Ellinghaus P, Milting H, Riedl B, Nikolova S, Pullamsetti SS, Weissmann N, Dony E, Savai R, Ghofrani HA, Grimminger F, Busch AE, Schäfer S. Combined tyrosine and serine/threonine kinase inhibition by sorafenib prevents progression of experimental pulmonary hypertension and myocardial remodeling. Circulation. 2008; 118: 2081-90.
146. Klinger JR, Warburton RR, Pietras L, Hill NS. Brain natriuretic peptide inhibits hypoxic pulmonary hypertension in rats. J Appl Physiol (1985). 1998; 84: 1646-52.
147. Klinke A, Möller A, Pekarova M, Ravekes T, Friedrichs K, Berlin M, Scheu KM, Kubala L, Kolarova H, Ambrozova G, Schermuly RT, Woodcock SR, Freeman BA, Rosenkranz S, Baldus S, Rudolph V, Rudolph TK. Protective effects of 10-nitro-oleic acid in a hypoxia-induced murine model of pulmonary hypertension. Am J Respir Cell Mol Biol. 2014; 51: 155-62.
148. Kojonazarov B1, Sydykov A, Pullamsetti SS, Luitel H, Dahal BK, Kosanovic D, Tian X, Majewski M, Baumann C, Evans S, Phillips P, Fairman D, Davie N, Wayman C, Kilty I, Weissmann N, Grimminger F, Seeger W, Ghofrani HA, Schermuly RT. Effects of multikinase inhibitors on pressure overload-induced right ventricular remodeling. Int J Cardiol. 2013; 167: 2630-7.
149. Kosanovic D, Kojonazarov B, Luitel H, Dahal BK, Sydykov A, Cornitescu T, Janssen W, Brandes RP, Davie N, Ghofrani HA, Weissmann N, Grimminger F, Seeger W, Schermuly RT. Therapeutic efficacy of TBC3711 in monocrotaline-induced pulmonary hypertension. Respir Res. 2011; 12: 87.
150. Koyama M, Furuhashi M, Ishimura S, Mita T, Fuseya T, Okazaki Y, Yoshida H, Tsuchihashi K, Miura T. Reduction of endoplasmic reticulum stress by 4-phenylbutyric acid prevents the development of hypoxia-induced pulmonary arterial hypertension. Am J Physiol Heart Circ Physiol. 2014; 306: H1314-23.
151. Kwon JH, Kim KC, Cho MS, Kim HS, Sohn S, Hong YM. An inhibitory effect of tumor necrosis factor-alpha antagonist to gene expression in monocrotaline-induced pulmonary hypertensive rats model. Korean J Pediatr. 2013; 56: 116-24.
152. Lahm T, Albrecht M, Fisher AJ, Selej M, Patel NG, Brown JA, Justice MJ, Brown MB, Van Demark M, Trulock KM, Dieudonne D, Reddy JG, Presson RG, Petrache I. 17ß-Estradiol attenuates hypoxic pulmonary hypertension via estrogen receptor-mediated effects. Am J Respir Crit Care Med. 2012; 185: 965-80.
153. Lai YL, Chen CF, Chien CT, Shiao HL, Thacker AA, Zhang HQ. Capsaicin pretreatment attenuates chronic hypoxic pulmonary hypertension. Respir Physiol. 1995; 99: 283-9.
154. Lai YL, Wu HD, Chen CF. Antioxidants attenuate chronic hypoxic pulmonary hypertension. J Cardiovasc Pharmacol. 1998; 32: 714-20.
155. Lan B, Hayama E, Kawaguchi N, Furutani Y, Nakanishi T. Therapeutic efficacy of valproic acid in a combined monocrotaline and chronic hypoxia rat model of severe pulmonary hypertension. PLoS One. 2015; 10: e0117211.
156. Lan TH, Chen XL, Wu YS, Qiu HL, Li JZ, Ruan XM, Xu DP, Lin DQ. 3,7-Bis(2-hydroxyethyl)icaritin, a potent inhibitor of phosphodiesterase-5, prevents monocrotaline-induced pulmonary arterial hypertension via NO/cGMP activation in rats. Eur J Pharmacol. 2018; 829: 102-111.
157. Laudi S, Trump S, Schmitz V, West J, McMurtry IF, Mutlak H, Christians U, Weimann J, Kaisers U, Steudel W. Serotonin transporter protein in pulmonary hypertensive rats treated with atorvastatin. Am J Physiol Lung Cell Mol Physiol. 2007; 293: L630-8.
158. Laursen BE, Dam MY, Mulvany MJ, Simonsen U. Hypoxia-induced pulmonary vascular remodeling and right ventricular hypertrophy is unaltered by long-term oral L-arginine administration. Vascul Pharmacol. 2008; 49: 71-6.
159. Lee DS, Jung YW. Protective Effect of Right Ventricular Mitochondrial Damage by Cyclosporine A in Monocrotaline-induced Pulmonary Hypertension. Korean Circ J. 2018; 48: 1135-1144.
160. Lee DS, Kim YK, Jung YW. Simvastatin, sildenafil and their combination in monocrotaline induced pulmonary arterial hypertension. Korean Circ J. 2010; 40: 659-64.
161. Lee JH, Park BK, Oh KS, Yi KY, Lim CJ, Seo HW, Lee BH. A urotensin II receptor antagonist, KR36676, decreases vascular remodeling and inflammation in experimental pulmonary hypertension. Int Immunopharmacol. 2016; 40:196-202.
162. Lee MY, Tsai KB, Hsu JH, Shin SJ, Wu JR, Yeh JL. Liraglutide prevents and reverses monocrotaline-induced pulmonary arterial hypertension by suppressing ET-1 and enhancing eNOS/sGC/PKG pathways. Sci Rep. 2016; 6:31788.
163. Lee Y, Pai SB, Bellamkonda RV, Thompson DH, Singh J. Cerivastatin Nanoliposome as a Potential Disease Modifying Approach for the Treatment of Pulmonary Arterial Hypertension. J Pharmacol Exp Ther. 2018; 366: 66-74.
164. Leong ZP, Okida A, Higuchi M, Yamano Y, Hikasa Y. Reversal effects of low-dose imatinib compared with sunitinib on monocrotaline-induced pulmonary and right ventricular remodeling in rats. Vascul Pharmacol. 2018; 100: 41-50.
165. Li C, Liu PP, Tang DD, Song R, Zhang YQ, Lei S, Wu SJ. Targeting the RhoA-ROCK pathway to regulate T-cell homeostasis in hypoxia-induced pulmonary arterial hypertension. Pulm Pharmacol Ther. 2018; 50: 111-122.
166. Li G, Liu Y, Zhu Y, Liu A, Xu Y, Li X, Li Z, Su J, Sun L. ACE2 activation confers endothelial protection and attenuates neointimal lesions in prevention of severe pulmonary arterial hypertension in rats. Lung. 2013; 191: 327-36.
167. Li G, Xu YL, Ling F, Liu AJ, Wang D, Wang Q, Liu YL. Angiotensin-converting enzyme 2 activation protects against pulmonary arterial hypertension through improving early endothelial function and mediating cytokines levels. Chin Med J (Engl). 2012; 125: 1381-8.
168. Li H, Lu W, Cai WW, Wang PJ, Zhang N, Yu CP, Wang DL, Liu BC, Sun W. Telmisartan attenuates monocrotaline-induced pulmonary artery endothelial dysfunction through a PPAR gamma-dependent PI3K/Akt/eNOS pathway. Pulm Pharmacol Ther. 2014; 28: 17-24.
169. Li J, Long C, Cui W, Wang H. Iptakalim ameliorates monocrotaline-induced pulmonary arterial hypertension in rats. J Cardiovasc Pharmacol Ther. 2013; 18: 60-9.
170. Li J, Shi QX, Fan R, Zhang LJ, Zhang SM, Guo HT, Wang YM, Kaye AJ, Kaye AD, Bueno FR, Xu XZ, Yu SQ, Yi DH, Pei JM. Vasculoprotective effect of U50,488H in rats exposed to chronic hypoxia: role of Akt-stimulated NO production. J Appl Physiol (1985). 2013; 114: 238-44.
171. Li J, Zhang P, Zhang QY, Zhang SM, Guo HT, Bi H, Wang YM, Sun X, Liu JC, Cheng L, Cui Q, Yu SQ, Kaye AD, Yi DH, Pei JM. Effects of U50,488H on hypoxia pulmonary hypertension and its underlying mechanism. Vascul Pharmacol. 2009; 51: 72-7.
172. Li L, Dong P, Hou C, Cao F, Sun S, He F, Song Y, Li S, Bai Y, Zhu D. Hydroxysafflor yellow A (HSYA) attenuates hypoxic pulmonary arterial remodelling and reverses right ventricular hypertrophy in rats. J Ethnopharmacol. 2016; 186: 224-233.
173. Li S, Han D, Zhang Y, Xie X, Ke R, Zhu Y, Liu L, Song Y, Yang L, Li M. Activation of AMPK Prevents Monocrotaline-Induced Extracellular Matrix Remodeling of Pulmonary Artery. Med Sci Monit Basic Res. 2016; 22: 27-33.
174. Li XL, Guan RJ, Li JJ. Attenuation of monocrotaline-induced pulmonary arterial hypertension in rats by rosuvastatin. J Cardiovasc Pharmacol. 2012; 60: 219-26.
175. Li XL, Guan RJ, Xu QH, Wu ZY. Effects of rosuvastatin on monocrotaline-induced pulmonary artery hypertension in rats. Zhonghua Xin Xue Guan Bing Za Zhi. 2011; 39: 247-53.
176. Li XQ, Hong Y, Wang Y, Zhang XH, Wang HL. Sertraline protects against monocrotaline-induced pulmonary hypertension in rats. Clin Exp Pharmacol Physiol. 2006; 33: 1047-51.
177. Li XW, Gao YX, Li S, Yang JR. Effect of sesamin on pulmonary vascular remodeling in rats with monocrotaline-induced pulmonary hypertension. Zhongguo Zhong Yao Za Zhi. 2015; 40: 1355-61.
178. Li XW, Guo B, Shen YY, Yang JR. Effect of chrysin on expression of NOX4 and NF-?B in right ventricle of monocrotaline-induced pulmonary arterial hypertension of rats. Yao Xue Xue Bao. 2015; 50: 1128-34.
179. Li XW, Wang XM, Li S, Yang JR. Effects of chrysin (5,7-dihydroxyflavone) on vascular remodeling in hypoxia-induced pulmonary hypertension in rats. Chin Med. 2015; 10: 4.
180. Li XW, Wang XM, Li S, Yang JR. Effects of rutaecarpine on right ventriclar remodeling in rats with monocrotaline-induced pulmonary hypertension. Zhongguo Ying Yong Sheng Li Xue Za Zhi. 2014; 30: 405-10.
181. Li Y, Wang Y, Li Y, Qian Z, Zhu L, Yang D. Osthole attenuates pulmonary arterial hypertension in monocrotaline‑treated rats. Mol Med Rep. 2017; 16: 2823-2829.
182. Li ZC, Zhang FQ, Song JC, Mei QB, Zhao DH. Therapeutic effects of DCDDP, a calcium channel blocker, on chronic pulmonary hypertension in rat. J Appl Physiol (1985). 2002; 92: 997-1003.
183. Lima-Seolin BG, Colombo R, Bonetto JHP, Teixeira RB, Donatti LM, Casali KR, Godoy AEG, Litvin IE, Schenkel PC, Rosa Araujo ASD, Belló-Klein A. Bucindolol improves right ventricle function in rats with pulmonary arterial hypertension through the reversal of autonomic imbalance. Eur J Pharmacol. 2017; 798: 57-65.
184. Lin Z, Jiang Z, Huang X, Cai X, Wang H, Xie L. Preventive treatment with atorvastatin ameliorates endothelial dysfunction of small pulmonary arteries in monocrotaline-induced pulmonary hypertensive rats. Clin Exp Hypertens. 2017; 39: 495-501.
185. Lingeshwar P, Kaur G, Singh N, Singh S, Mishra A, Shukla S, Ramakrishna R, Laxman TS, Bhatta RS, Siddiqui HH, Hanif K. A study on the involvement of GABA-transaminase in MCT induced pulmonary hypertension. Pulm Pharmacol Ther. 2016; 36: 10-21.
186. Li-sheng Li, Yun-mei Luo, Juan Liu, Yu Zhang, Xiao-xia Fu, and Dan-li Yang. Icariin Inhibits Pulmonary Hypertension Induced by Monocrotaline through Enhancement of NO/cGMP Signaling Pathway in Rats. Evid Based Complement Alternat Med. 2016; 7915415.
187. Liu A, Hacker T, Eickhoff JC, Chesler NC. Estrogen Preserves Pulsatile Pulmonary Arterial Hemodynamics in Pulmonary Arterial Hypertension. Ann Biomed Eng. 2017; 45: 632-643.
188. Liu A, Philip J, Vinnakota KC, Van den Bergh F, Tabima DM, Hacker T, Beard DA, Chesler NC. Estrogen maintains mitochondrial content and function in the right ventricle of rats with pulmonary hypertension. Physiol Rep. 2017; 5. pii: e13157.
189. Liu B, Luo XJ, Yang ZB, Zhang JJ, Li TB, Zhang XJ, Ma QL, Zhang GG, Hu CP, Peng J.Inhibition of NOX/VPO1 pathway and inflammatory reaction by trimethoxystilbene in prevention of cardiovascular remodeling in hypoxia-induced pulmonary hypertensive rats. J Cardiovasc Pharmacol. 2014; 63: 567-76.
190. Liu B, Wang XM, Zhou TF, Hua YM, Liu HM, Wei L, Qiao LN, Wang XQ, Zhao SS, Shi K. Expression of connective tissue growth factor and its down-regulation by simvastatin administration in pulmonary hypertensive rats. Zhonghua Er Ke Za Zhi. 2008; 46: 359-65.
191. Liu B, Wang XQ, Yu L, Zhou TF, Wang XM, Liu HM. Simvastatin restores down-regulated GATA-6 expression in pulmonary hypertensive rats. Exp Lung Res. 2009; 35: 411-26.
192. Liu C, Fang C, Cao G, Liu K, Wang B, Wan Z, Li S, Wu S. Ethyl pyruvate ameliorates monocrotaline-induced pulmonary arterial hypertension in rats. J Cardiovasc Pharmacol. 2014; 64: 7-15.
193. Liu J, Cai G, Li M, Fan S, Yao B, Ping W, Huang Z, Cai H, Dai Y, Wang L, Huang X. Fibroblast growth factor 21 attenuates hypoxia-induced pulmonary hypertension by upregulating PPARγ expression and suppressing inflammatory cytokine levels. Biochem Biophys Res Commun. 2018; 504: 478-484.
194. Liu M, Wang Y, Zheng L, Zheng W, Dong K, Chen S, Zhang B, Li Z. Fasudil reversed MCT-induced and chronic hypoxia-induced pulmonary hypertension by attenuating oxidative stress and inhibiting the expression of Trx1 and HIF-1?. Respir Physiol Neurobiol. 2014; 201: 38-46.
195. Liu P, Yan S, Chen M, Chen A, Yao D, Xu X, Cai X, Wang L, Huang X. Effects of baicalin on collagen I and collagen III expression in pulmonary arteries of rats with hypoxic pulmonary hypertension. Int J Mol Med. 2015; 35: 901-8.
196. Liu Y, Tian H, Yan X, Fan F, Wang W, Han J. Serotonin inhibits apoptosis of pulmonary artery smooth muscle cells through 5-HT2A receptors involved in the pulmonary artery remodeling of pulmonary artery hypertension. Exp Lung Res. 2013; 39: 70-9.
197. Liu Y, Tian XY, Mao G, Fang X, Fung ML, Shyy JY, Huang Y, Wang N. Peroxisome proliferator-activated receptor-γ ameliorates pulmonary arterial hypertension by inhibiting 5-hydroxytryptamine 2B receptor. Hypertension. 2012; 60: 1471-8.
198. Liu Y, Zhang B, Dong MQ, Niu W, Luo Y, Gao YQ, Li ZC. Attenuation of pulmonary arterial smooth muscle cell proliferation following hypoxic pulmonary hypertension by the Na+/H+ exchange inhibitor amiloride. Chin J Physiol. 2010; 53: 36-44.
199. Liu ZQ, Liu B, Yu L, Wang XQ, Wang J, Liu HM. Simvastatin has beneficial effect on pulmonary artery hypertension by inhibiting NF-?B expression. Mol Cell Biochem. 2011; 354: 77-82.
200. Lu Y, Guo H, Sun Y, Pan X, Dong J, Gao D, Chen W, Xu Y, Xu D. Valsartan attenuates pulmonary hypertension via suppression of mitogen activated protein kinase signaling and matrix metalloproteinase expression in rodents. Mol Med Rep. 2017; 16: 1360-1368.
201. Luan Y, Chao S, Ju ZY, Wang J, Xue X, Qi TG, Cheng GH, Kong F. Therapeutic effects of baicalin on monocrotaline-induced pulmonary arterial hypertension by inhibiting inflammatory response. Int Immunopharmacol. 2015; 26: 188-93.
202. Ma W, Han W, Greer PA, Tuder RM, Toque HA, Wang KK, Caldwell RW, Su Y. Calpain mediates pulmonary vascular remodeling in rodent models of pulmonary hypertension, and its inhibition attenuates pathologic features of disease. J Clin Invest. 2011; 121: 4548-66.
203. MacRitchie N, Volpert G, Al Washih M, Watson DG, Futerman AH, Kennedy S, Pyne S, Pyne NJ. Effect of the sphingosine kinase 1 selective inhibitor, PF-543 on arterial and cardiac remodelling in a hypoxic model of pulmonary arterial hypertension. Cell Signal. 2016; 28: 946-55.
204. Mao SZ, Hong L, Hu LG, Fan XF, Zhang L, Guo YM, Gong YS. Effect of apelin on hypoxic pulmonary hypertension in rats: role of the NO pathway. Sheng Li Xue Bao. 2009; 61: 480-4.
205. Marcos E, Adnot S, Pham MH, Nosjean A, Raffestin B, Hamon M, Eddahibi S. Serotonin transporter inhibitors protect against hypoxic pulmonary hypertension. Am J Respir Crit Care Med. 2003; 168: 487-93.
206. Maron BA, Zhang YY, White K, Chan SY, Handy DE, Mahoney CE, Loscalzo J, Leopold JA. Aldosterone inactivates the endothelin-B receptor via a cysteinyl thiol redox switch to decrease pulmonary endothelial nitric oxide levels and modulate pulmonary arterial hypertension. Circulation. 2012; 126: 963-74.
207. Matori H, Umar S, Nadadur RD, Sharma S, Partow-Navid R, Afkhami M, Amjedi M, Eghbali M. Genistein, a soy phytoestrogen, reverses severe pulmonary hypertension and prevents right heart failure in rats. Hypertension. 2012; 60: 425-30.
208. Mawatari E, Hongo M, Sakai A, Terasawa F, Takahashi M, Yazaki Y, Kinoshita O, Ikeda U. Amlodipine prevents monocrotaline-induced pulmonary arterial hypertension and prolongs survival in rats independent of blood pressure lowering. Clin Exp Pharmacol Physiol. 2007; 34: 594-600.
209. Maxová H, Hezinová A, Vízek M. Disodium cromoglycate attenuates hypoxia induced enlargement of end-expiratory lung volume in rats. Physiol Res. 2011; 60: 831-4.
210. Maxová H, Vasilková M, Novotná J, Vajnerová O, Bansová A, Vízek M, Herget J. Prevention of mast cell degranulation by disodium cromoglycate delayed the regression of hypoxic pulmonary hypertension in rats. Respiration. 2010; 80: 335-9.
211. McMurtry MS, Bonnet S, Michelakis ED, Bonnet S, Haromy A, Archer SL. Statin therapy, alone or with rapamycin, does not reverse monocrotaline pulmonary arterial hypertension: the rapamcyin-atorvastatin-simvastatin study. Am J Physiol Lung Cell Mol Physiol. 2007; 293: L933-40.
212. Medarametla V, Festin S, Sugarragchaa C, Eng A, Naqwi A, Wiedmann T, Zisman LS. PK10453, a nonselective platelet-derived growth factor receptor inhibitor, prevents the progression of pulmonary arterial hypertension. Pulm Circ. 2014; 4: 82-102.
213. Megalou AJ, Glava C, Oikonomidis DL, Vilaeti A, Agelaki MG, Baltogiannis GG, Papalois A, Vlahos AP, Kolettis TM. Transforming growth factor-ß inhibition attenuates pulmonary arterial hypertension in rats. Int J Clin Exp Med. 2010; 3: 332-40.
214. Megalou AJ, Glava C, Vilaeti AD, Oikonomidis DL, Baltogiannis GG, Papalois A, Vlahos AP, Kolettis TM. Transforming growth factor-ß inhibition and endothelin receptor blockade in rats with monocrotaline-induced pulmonary hypertension. Pulm Circ. 2012; 2: 461-9.
215. Meghwani H, Prabhakar P, Mohammed SA, Dua P, Seth S, Hote MP, Banerjee SK, Arava S, Ray R, Maulik SK. Beneficial Effect of Ocimum sanctum (Linn) against Monocrotaline-Induced Pulmonary Hypertension in Rats. Medicines (Basel). 2018; 5. pii: E34.
216. Meghwani H, Prabhakar P, Mohammed SA, Seth S, Hote MP, Banerjee SK, Arava S, Ray R, Maulik SK. Beneficial effects of aqueous extract of stem bark of Terminalia arjuna (Roxb.), An ayurvedic drug in experimental pulmonary hypertension. J Ethnopharmacol. 2017; 197: 184-194.
217. Mei Y, Jin H, Tian W, Wang H, Wang H, Zhao Y, Zhang Z, Meng F. Urantide alleviates monocrotaline induced pulmonary arterial hypertension in Wistar rats. Pulm Pharmacol Ther. 2011; 24: 386-93.
218. Mendes-Ferreira P1, Maia-Rocha C1, Adão R1, Mendes MJ1, Santos-Ribeiro D1, Alves BS1, Cerqueira RJ1, Castro-Chaves P1, Lourenço AP1, De Keulenaer GW2, Leite-Moreira AF1, Brás-Silva C. Neuregulin-1 improves right ventricular function and attenuates experimental pulmonary arterial hypertension. Cardiovasc Res. 2016; 109: 44-54.
219. Michelakis ED, McMurtry MS, Wu XC, Dyck JR, Moudgil R, Hopkins TA, Lopaschuk GD, Puttagunta L, Waite R, Archer SL. Dichloroacetate, a metabolic modulator, prevents and reverses chronic hypoxic pulmonary hypertension in rats: role of increased expression and activity of voltage-gated potassium channels. Circulation. 2002; 105: 244-50.
220. Mitani Y, Maruyama K, Sakurai M. Prolonged administration of L-arginine ameliorates chronic pulmonary hypertension and pulmonary vascular remodeling in rats. Circulation. 1997; 96: 689-97.
221. Miyata M, Ito M, Sasajima T, Ohira H, Sato Y, Kasukawa R. Development of monocrotaline-induced pulmonary hypertension is attenuated by a serotonin receptor antagonist. Lung. 2000; 178: 63-73.
222. Morales-Cano D, Menendez C, Moreno E, Moral-Sanz J, Barreira B, Galindo P, Pandolfi R, Jimenez R, Moreno L, Cogolludo A, Duarte J, Perez-Vizcaino F. The flavonoid quercetin reverses pulmonary hypertension in rats. PLoS One. 2014; 9: e114492.
223. Morecroft I, Pang L, Baranowska M, Nilsen M, Loughlin L, Dempsie Y, Millet C, MacLean MR. In vivo effects of a combined 5-HT1B receptor/SERT antagonist in experimental pulmonary hypertension. Cardiovasc Res. 2010; 85: 593-603.
224. Morel OE, Buvry A, Le Corvoisier P, Tual L, Favret F, León-Velarde F, Crozatier B, Richalet JP. Effects of nifedipine-induced pulmonary vasodilatation on cardiac receptors and protein kinase C isoforms in the chronically hypoxic rat. Pflugers Arch. 2003; 446: 356-64.
225. Morin C, Hiram R, Rousseau E, Blier PU, Fortin S. Docosapentaenoic acid monoacylglyceride reduces inflammation and vascular remodeling in experimental pulmonary hypertension. Am J Physiol Heart Circ Physiol. 2014; 307: H574-86.
226. Mouchaers KT, Schalij I, de Boer MA, Postmus PE, van Hinsbergh VW, van Nieuw Amerongen GP, Vonk Noordegraaf A, van der Laarse WJ. Fasudil reduces monocrotaline-induced pulmonary arterial hypertension: comparison with bosentan and sildenafil. Eur Respir J. 2010; 36: 800-7.
227. Mourelle M, Martin MT, Giménez F. Treatment with LA-419 prevents monocrotaline-induced pulmonary hypertension and lung injury in the rat. Proc West Pharmacol Soc. 2011; 54: 89-93.
228. Murata T, Kinoshita K, Hori M, Kuwahara M, Tsubone H, Karaki H, Ozaki H. Statin protects endothelial nitric oxide synthase activity in hypoxia-induced pulmonary hypertension. Arterioscler Thromb Vasc Biol. 2005; 25: 2335-42.
229. Murugesan P, Hildebrandt T, Bernlöhr C, Lee D, Khang G, Doods H, Wu D. Inhibition of kinin B1 receptors attenuates pulmonary hypertension and vascular remodeling. Hypertension. 2015; 66: 906-12.
230. Nagata T, Uehara Y, Hara K, Igarashi K, Hazama H, Hisada T, Kimura K, Goto A, Omata M. Thromboxane inhibition and monocrotaline-induced pulmonary hypertension in rats. Respirology. 1997; 2: 283-9.
231. Nagaya N, Okumura H, Uematsu M, Shimizu W, Ono F, Shirai M, Mori H, Miyatake K, Kangawa K. Repeated inhalation of adrenomedullin ameliorates pulmonary hypertension and survival in monocrotaline rats. Am J Physiol Heart Circ Physiol. 2003; 285: H2125-31.
232. Nakamura A, Nagaya N, Obata H, Sakai K, Sakai Y, Yoshikawa M, Hamada K, Matsumoto K, Kimura H. Oral administration of a novel long-acting prostacyclin agonist with thromboxane synthase inhibitory activity for pulmonary arterial hypertension. Circ J. 2013; 77: 2127-33.
233. Nakata TM, Tanaka R, Yoshiyuki R, Fukayama T, Goya S, Fukushima R. Effects of Single Drug and Combined Short-term Administration of Sildenafil, Pimobendan, and Nicorandil on Right Ventricular Function in Rats With Monocrotaline-induced Pulmonary Hypertension. J Cardiovasc Pharmacol. 2015; 65: 640-8.
234. Nan X, Su S, Ma K, Ma X, Wang X, Zhaxi D, Ge R, Li Z, Lu D. Bioactive fraction of Rhodiola algida against chronic hypoxia-induced pulmonary arterial hypertension and its anti-proliferation mechanism in rats. J Ethnopharmacol. 2018; 216: 175-183.
235. Nassar SZ, Hassaan PS, Abdelmonsif DA, ElAchy SN. Cardioprotective effect of cerium oxide nanoparticles in monocrotaline rat model of pulmonary hypertension: A possible implication of endothelin-1. Life Sci. 2018; 201: 89-101.
236. Nickel NP, Spiekerkoetter E, Gu M, Li CG, Li H, Kaschwich M, Diebold I, Hennigs JK, Kim KY, Miyagawa K, Wang L, Cao A, Sa S, Jiang X, Stockstill RW, Nicolls MR, Zamanian RT, Bland RD, Rabinovitch M. Elafin Reverses Pulmonary Hypertension via Caveolin-1-Dependent Bone Morphogenetic Protein Signaling. Am J Respir Crit Care Med. 2015; 191: 1273-86.
237. Nisbet RE, Bland JM, Kleinhenz DJ, Mitchell PO, Walp ER, Sutliff RL, Hart CM. Rosiglitazone attenuates chronic hypoxia-induced pulmonary hypertension in a mouse model. Am J Respir Cell Mol Biol. 2010; 42: 482-90.
238. Nishida M, Hasegawa Y, Tanida I, Nakagawa E, Inaji H, Ohkita M, Matsumura Y. Preventive effects of raloxifene, a selective estrogen receptor modulator, on monocrotaline-induced pulmonary hypertension in intact and ovariectomized female rats. Eur J Pharmacol. 2009; 614: 70-6.
239. Nishimura T, Faul JL, Berry GJ, Vaszar LT, Qiu D, Pearl RG, Kao PN. Simvastatin attenuates smooth muscle neointimal proliferation and pulmonary hypertension in rats. Am J Respir Crit Care Med. 2002; 166: 1403-8.
240. Nishimura T, Faul JL, Berry GJ, Veve I, Pearl RG, Kao PN. 40-O-(2-hydroxyethyl)-rapamycin attenuates pulmonary arterial hypertension and neointimal formation in rats. Am J Respir Crit Care Med. 2001; 163: 498-502.
241. Nogueira-Ferreira R, Ferreira-Pinto MJ, Silva AF, Vitorino R, Justino J, Costa R, Moreira-Gonçalves D, Quignard JF, Ducret T, Savineau JP, Leite-Moreira AF, Ferreira R, Henriques-Coelho T. HMGB1 down-regulation mediates terameprocol vascular anti-proliferative effect in experimental pulmonary hypertension. J Cell Physiol. 2017; 232: 3128-3138.
242. Nong Z, Stassen JM, Moons L, Collen D, Janssens S. Inhibition of tissue angiotensin-converting enzyme with quinapril reduces hypoxic pulmonary hypertension and pulmonary vascular remodeling. Circulation. 1996; 94: 1941-7.
243. Okada K, Bernstein ML, Zhang W, Schuster DP, Botney MD. Angiotensin-converting enzyme inhibition delays pulmonary vascular neointimal formation. Am J Respir Crit Care Med. 1998; 158: 939-50.
244. Onat AM, Pehlivan Y, Turkbeyler IH, Demir T, Kaplan DS, Ceribasi AO, Orkmez M, Tutar E, Taysi S, Sayarlioglu M, Kisacik B. Urotensin inhibition with palosuran could be a promising alternative in pulmonary arterial hypertension. Inflammation. 2013; 36: 405-12.
245. Ono S, Voelkel NF. PAF receptor blockade inhibits lung vascular changes in the rat monocrotaline model. Lung. 1992; 170: 31-40.
246. Ono S, Westcott JY, Voelkel NF. PAF antagonists inhibit pulmonary vascular remodeling induced by hypobaric hypoxia in rats. J Appl Physiol (1985). 1992; 73: 1084-92.
247. Ou ZJ, Wei W, Huang DD, Luo W, Luo D, Wang ZP, Zhang X, Ou JS. L-arginine restores endothelial nitric oxide synthase-coupled activity and attenuates monocrotaline-induced pulmonary artery hypertension in rats. Am J Physiol Endocrinol Metab. 2010; 298: E1131-9.
248. Özlem Atlı, Sinem Ilgın, Bülent Ergun, Dilek Burukoğlu, Ahmet Musmul, and Başar Sırmagül. Matrix metalloproteinases are possible targets in monocrotaline-induced pulmonary hypertension: investigation of anti-remodeling effects of alagebrium and everolimus. Anatol J Cardiol. 2017; 17: 8–17.
249. Paffett ML, Lucas SN, Campen MJ. Resveratrol reverses monocrotaline-induced pulmonary vascular and cardiac dysfunction: a potential role for atrogin-1 in smooth muscle. Vascul Pharmacol. 2012; 56: 64-73.
250. Paffett ML, Meghan M. Channell, Jay S. Naik, Selita N. Lucas, and Matthew J. Campen. Cardiac and Vascular Atrogin-1 mRNA Expression is Not Associated with Dexamethasone Efficacy in the Monocrotaline Model of Pulmonary Hypertension. Cardiovasc Toxicol . 2012; 12: 226–234.
251. Pankey EA, Badejo AM, Casey DB, Lasker GF, Riehl RA, Murthy SN, Nossaman BD, Kadowitz PJ. Effect of chronic sodium nitrite therapy on monocrotaline-induced pulmonary hypertension. Nitric Oxide. 2012; 27: 1-8.
252. Pankey EA, Thammasiboon S, Lasker GF, Baber S, Lasky JA, Kadowitz PJ. Imatinib attenuates monocrotaline pulmonary hypertension and has potent vasodilator activity in pulmonary and systemic vascular beds in the rat. Am J Physiol Heart Circ Physiol. 2013; 305: H1288-96.
253. **Pankova NV, Artem'eva MM, Medvedeva NA. [INFLUENCE OF THE FEMALE SEX HORMONE 17-BETA-ESTRADIOL ON THE DEGREE OF HYPOXIC PULMONARY HYPERTENSION IN MALE AND FEMALE WISTAR RATS.] Eksp Klin Farmakol. 2017;80(1):9-13.**
254. Pehlivan Y, Dokuyucu R, Demir T, Kaplan DS, Koc I, Orkmez M, Turkbeyler IH, Ceribasi AO, Tutar E, Taysi S, Kisacik B, Onat AM. Palosuran treatment effective as bosentan in the treatment model of pulmonary arterial hypertension. Inflammation. 2014; 37: 1280-8.
255. Pei JM, Sun X, Guo HT, Ma S, Zang YM, Lu SY, Bi H, Wang YM, Ma H, Ma XL. U50,488H depresses pulmonary pressure in rats subjected to chronic hypoxia. J Cardiovasc Pharmacol. 2006; 47: 594-8.
256. Pei Y, Ma P, Wang X, Zhang W, Zhang X, Zheng P, Yan L, Xu Q, Dai G. Rosuvastatin attenuates monocrotaline-induced pulmonary hypertension via regulation of Akt/eNOS signaling and asymmetric dimethylarginine metabolism. Eur J Pharmacol. 2011; 666: 165-72.
257. Pena A, Kobir A, Goncharov D, Goda A, Kudryashova TV, Ray A, Vanderpool R, Baust J, Chang B, Mora AL, Gorcsan J, Goncharova EA. Pharmacological Inhibition of mTOR Kinase Reverses Right Ventricle Remodeling and Improves Right Ventricle Structure and Function in Rats. Am J Respir Cell Mol Biol. 2017; 57: 615-625.
258. Pereira SL, Kummerle AE, Fraga CA, Barreiro EJ, Rocha Nde N, Ferraz EB, do Nascimento JH, Sudo RT, Zapata-Sudo G. A novel Ca2+ channel antagonist reverses cardiac hypertrophy and pulmonary arteriolar remodeling in experimental pulmonary hypertension. Eur J Pharmacol. 2013; 702: 316-22.
259. Perros F, Ranchoux B, Izikki M, Bentebbal S, Happé C, Antigny F, Jourdon P, Dorfmüller P, Lecerf F, Fadel E, Simonneau G, Humbert M, Bogaard HJ, Eddahibi S. Nebivolol for Improving Endothelial Dysfunction, Pulmonary Vascular Remodeling, and Right Heart Function in Pulmonary Hypertension. J Am Coll Cardiol. 2015; 65: 668-681.
260. Pichon A, Connes P, Quidu P, Marchant D, Brunet J, Levy BI, Vilar J, Safeukui I, Cymbalista F, Maignan M, Richalet JP, Favret F. Acetazolamide and chronic hypoxia: effects on haemorheology and pulmonary haemodynamics. Eur Respir J. 2012; 40: 1401-9.
261. Pidgeon GP, Tamosiuniene R, Chen G, Leonard I, Belton O, Bradford A, Fitzgerald DJ. Intravascular thrombosis after hypoxia-induced pulmonary hypertension: regulation by cyclooxygenase-2. Circulation. 2004; 110: 2701-7.
262. Poble PB Phan C, Quatremare T, Bordenave J, Thuillet R, Cumont A, Huertas A, Tu L, Dorfmüller P, Humbert M, Ghigna MR, Savale L, Guignabert C. Therapeutic effect of pirfenidone in the sugen/hypoxia rat model of severe pulmonary hypertension. FASEB J. 2019; 33: 3670-3679.
263. Polonio IB, Acencio MM, Pazetti R, Almeida FM, Silva BS, Pereira KA, Souza R. Lodenafil treatment in the monocrotaline model of pulmonary hypertension in rats. J Bras Pneumol. 2014; 40: 421-4.
264. Porvasnik SL, Germain S, Embury J, Gannon KS, Jacques V, Murray J, Byrne BJ, Shacham S, Al-Mousily F. PRX-08066, a novel 5-hydroxytryptamine receptor 2B antagonist, reduces monocrotaline-induced pulmonary arterial hypertension and right ventricular hypertrophy in rats. J Pharmacol Exp Ther. 2010; 334: 364-72.
265. Preston IR, Sagliani KD, Warburton RR, Hill NS, Fanburg BL, Jaffe IZ. Mineralocorticoid receptor antagonism attenuates experimental pulmonary hypertension. Am J Physiol Lung Cell Mol Physiol. 2013; 304: L678-88.
266. Price LC, Montani D, Tcherakian C, Dorfmüller P, Souza R, Gambaryan N, Chaumais MC, Shao DM, Simonneau G, Howard LS, Adcock IM, Wort SJ, Humbert M, Perros F. Dexamethasone reverses monocrotaline-induced pulmonary arterial hypertension in rats. Eur Respir J. 2011; 37: 813-22.
267. Prins KW, Tian L, Wu D, Thenappan T, Metzger JM, Archer SL. Colchicine Depolymerizes Microtubules, Increases Junctophilin-2, and Improves Right Ventricular Function in Experimental Pulmonary Arterial Hypertension. J Am Heart Assoc. 2017; 6. pii: e006195.
268. Pullamsetti S, Krick S, Yilmaz H, Ghofrani HA, Schudt C, Weissmann N, Fuchs B, Seeger W, Grimminger F, Schermuly RT. Inhaled tolafentrine reverses pulmonary vascular remodeling via inhibition of smooth muscle cell migration. Respir Res. 2005; 6: 128.
269. Pullamsetti SS, Savai R, Schaefer MB, Wilhelm J, Ghofrani HA, Weissmann N, Schudt C, Fleming I, Mayer K, Leiper J, Seeger W, Grimminger F, Schermuly RT. cAMP phosphodiesterase inhibitors increases nitric oxide production by modulating dimethylarginine dimethylaminohydrolases. Circulation. 2011; 123: 1194-204.
270. Puukila S, Fernandes RO, Türck P, Carraro CC, Bonetto JHP, de Lima-Seolin BG, da Rosa Araujo AS, Belló-Klein A, Boreham D, Khaper N. Secoisolariciresinol diglucoside attenuates cardiac hypertrophy and oxidative stress in monocrotaline-induced right heart dysfunction. Mol Cell Biochem. 2017; 432: 33-39.
271. Qi J, Du J, Wang L, Zhao B, Tang C. Alleviation of hypoxic pulmonary vascular structural remodeling by L-arginine. Chin Med J (Engl). 2001; 114: 933-6.
272. Qi J, Du J, Zhao B. The mechanism responsible for alleviation of hypoxic pulmonary vascular structural remodeling by L-arginine. Zhonghua Yi Xue Za Zhi. 2000; 80: 214-8.
273. Quinn DA, Du HK, Thompson BT, Hales CA. Amiloride analogs inhibit chronic hypoxic pulmonary hypertension. Am J Respir Crit Care Med. 1998; 157: 1263-8.
274. Rakotoniaina Z, Guerard P, Lirussi F, Goirand F, Rochette L, Dumas M, Bardou M. The protective effect of HMG-CoA reductase inhibitors against monocrotaline-induced pulmonary hypertension in the rat might not be a class effect: comparison of pravastatin and atorvastatin. Naunyn Schmiedebergs Arch Pharmacol. 2006; 374: 195-206.
275. Rakotoniaina Z, Guerard P, Lirussi F, Rochette L, Dumas M, Goirand F, Bardou M. Celecoxib but not the combination of celecoxib+atorvastatin prevents the development of monocrotaline-induced pulmonary hypertension in the rat. Naunyn Schmiedebergs Arch Pharmacol. 2008; 378: 241-51.
276. Rashid J, Nozik-Grayck E, McMurtry IF, Stenmark KR, Ahsan F. Inhaled combination of sildenafil and rosiglitazone improves pulmonary hemodynamics, cardiac function, and arterial remodeling. Am J Physiol Lung Cell Mol Physiol. 2019; 316: L119-L130.
277. Ribeiro EL, Fragoso IT, Gomes FODS, Oliveira AC, Silva AKSE, Silva PME, Ciambarella BT, Ramos IPR, Peixoto CA. Diethylcarbamazine: A potential treatment drug for pulmonary hypertension? Toxicol Appl Pharmacol. 2017; 333: 92-99.
278. Rocchetti M, Sala L, Rizzetto R, Staszewsky LI, Alemanni M, Zambelli V, Russo I, Barile L, Cornaghi L, Altomare C, Ronchi C, Mostacciuolo G, Lucchetti J, Gobbi M, Latini R, Zaza A. Ranolazine prevents INaL enhancement and blunts myocardial remodelling in a model of pulmonary hypertension. Cardiovasc Res. 2014; 104: 37-48.
279. Sahara M, Sata M, Morita T, Hirata Y, Nagai R. Nicorandil attenuates monocrotaline-induced vascular endothelial damage and pulmonary arterial hypertension. PLoS One. 2012; 7: e33367.
280. Sakamoto Y, Kameshima S, Kakuda C, Okamura Y, Kodama T, Okada M, Yamawaki H. Visceral adipose tissue-derived serine protease inhibitor prevents the development of monocrotaline-induced pulmonary arterial hypertension in rats. Pflugers Arch. 2017; 469: 1425-1432.
281. Samillan V1, Haider T2, Vogel J2, Leuenberger C3, Brock M4, Schwarzwald C5, Gassmann M6, Ostergaard L2. Combination of erythropoietin and sildenafil can effectively attenuate hypoxia-induced pulmonary hypertension in mice. Pulm Circ. 2013; 3: 898-907.
282. Satoh M, Satoh A. 3-Hydroxy-3-methylglutaryl (HMG)-COA reductase inhibitors and phosphodiesterase type V inhibitors attenuate right ventricular pressure and remodeling in a rat model of pulmonary hypertension. J Pharm Pharm Sci. 2009; 11: 118s-130s.
283. Sawada H, Mitani Y, Maruyama J, Jiang BH, Ikeyama Y, Dida FA, Yamamoto H, Imanaka-Yoshida K, Shimpo H, Mizoguchi A, Maruyama K, Komada Y. A nuclear factor-kappaB inhibitor pyrrolidine dithiocarbamate ameliorates pulmonary hypertension in rats. Chest. 2007; 132: 1265-74.
284. Schermuly RT, Kreisselmeier KP, Ghofrani HA, Samidurai A, Pullamsetti S, Weissmann N, Schudt C, Ermert L, Seeger W, Grimminger F. Antiremodeling effects of iloprost and the dual-selective phosphodiesterase 3/4 inhibitor tolafentrine in chronic experimental pulmonary hypertension. Circ Res. 2004; 94: 1101-8.
285. Schreiber C, Eilenberg MS, Panzenboeck A, Winter MP, Bergmeister H, Herzog R, Mascherbauer J, Lang IM, Bonderman D. Combined oral administration of L-arginine and tetrahydrobiopterin in a rat model of pulmonary arterial hypertension. Pulm Circ. 2017; 7: 89-97.
286. Schwenke DO, Tokudome T, Shirai M, Hosoda H, Horio T, Kishimoto I, Kangawa K. Exogenous ghrelin attenuates the progression of chronic hypoxia-induced pulmonary hypertension in conscious rats. Endocrinology. 2008; 149: 237-44.
287. Segura-Ibarra V, Amione-Guerra J, Cruz-Solbes AS, Cara FE, Iruegas-Nunez DA, Wu S, Youker KA, Bhimaraj A, Torre-Amione G, Ferrari M, Karmouty-Quintana H, Guha A, Blanco E. Rapamycin nanoparticles localize in diseased lung vasculature and prevent pulmonary arterial hypertension. Int J Pharm. 2017; 524: 257-267.
288. Shen L, Shen J, Pu J, He B. Aspirin attenuates pulmonary arterial hypertension in rats by reducing plasma 5-hydroxytryptamine levels. Cell Biochem Biophys. 2011; 61: 23-31.
289. Shi K, Qiao LN, Liu B, Zhao SS, Zhou TF, Wang XM, Wei L, Liu HM, Hua YM. Effect of doxycycline on the development of pulmonary hypertension induced by four methods in rats. Zhonghua Er Ke Za Zhi. 2009; 47: 260-4.
290. Shi R, Wei Z, Zhu D, Fu N, Wang C, Yin S, Liang Y, Xing J, Wang X, Wang Y. Baicalein attenuates monocrotaline-induced pulmonary arterial hypertension by inhibiting vascular remodeling in rats. Pulm Pharmacol Ther. 2018; 48: 124-135.
291. Shi R, Zhu D, Wei Z, Fu N, Wang C, Liu L, Zhang H, Liang Y, Xing J, Wang X, Wang Y. Baicalein attenuates monocrotaline-induced pulmonary arterial hypertension by inhibiting endothelial-to-mesenchymal transition. Life Sci. 2018; 207: 442-450.
292. Shi W, Zhai C, Feng W, Wang J, Zhu Y, Li S, Wang Q, Zhang Q, Yan X, Chai L, Liu P, Chen Y, Li M. Resveratrol inhibits monocrotaline-induced pulmonary arterial remodeling by suppression of SphK1-mediated NF-κB activation. Life Sci. 2018; 210: 140-149.
293. Sun LY, Cai ZY, Pu J, Li J, Shen JY, Yang CD, He B. 5-Aminosalicylic Acid Attenuates Monocrotaline-Induced Pulmonary Arterial Hypertension in Rats by Increasing the Expression of Nur77. Inflammation. 2017; 40: 806-817.
294. Sun X, Ku DD. Rosuvastatin provides pleiotropic protection against pulmonary hypertension, right ventricular hypertrophy, and coronary endothelial dysfunction in rats. Am J Physiol Heart Circ Physiol. 2008; 294: H801-9.
295. Sun XZ, Shu-Yan Li, Xiang-Yang Tian, Qing-Quan Wu. Effect of fasudil on hypoxic pulmonary hypertension and right ventricular hypertrophy in rats. Int J Clin Exp Pathol 2015; 8: 9517-9521.
296. Sun XZ, Tian XY, Wang DW, Li J. Effects of fasudil on hypoxic pulmonary hypertension and pulmonary vascular remodeling in rats. Eur Rev Med Pharmacol Sci. 2014; 18: 959-64.
297. Suzuki C, Takahashi M, Morimoto H, Izawa A, Ise H, Hongo M, Hoshikawa Y, Ito T, Miyashita H, Kobayashi E, Shimada K, Ikeda U. Mycophenolate mofetil attenuates pulmonary arterial hypertension in rats. Biochem Biophys Res Commun. 2006; 349: 781-8.
298. Suzuki R, Maehara R, Kobuchi S, Tanaka R, Ohkita M, Matsumura Y. Beneficial effects of γ-aminobutyric acid on right ventricular pressure and pulmonary vascular remodeling in experimental pulmonary hypertension.nLife Sci. 2012; 91: 693-8.
299. Takahashi T, Kanda T, Imai S, Suzuki T, Kobayashi I, Murata K. Semotiadil inhibits the development of right ventricular hypertrophy and medial thickening of pulmonary arteries in a rat model of pulmonary hypertension. Cardiovasc Drugs Ther. 1995; 9: 809-14.
300. Takahashi T, Kanda T, Inoue M, Suzuki T, Kobayashi I, Kodama K, Nagai R. A selective type V phosphodiesterase inhibitor, E4021, protects the development of right ventricular overload and medial thickening of pulmonary arteries in a rat model of pulmonary hypertension. Life Sci. 1996; 59: 371-7.
301. Tan JX, Chen XM, Fang X, Tao MJ, Huang XL. Effects of continuous adenosine infusion on pulmonary hypertension in chronically hypoxic rats. Nan Fang Yi Ke Da Xue Xue Bao. 2008; 28: 1052-5.
302. Tan JX, Huang XL, Wang B, Fang X, Huang DN. Adenosine receptors agonists mitigated PAH of rats induced by chronic hypoxia through reduction of renin activity/angiotensin II levels and increase of inducible nitric oxide synthase-nitric oxide levels. Zhonghua Er Ke Za Zhi. 2012; 50: 782-7.
303. Tang B, Chen GX, Liang MY, Yao JP, Wu ZK. Ellagic acid prevents monocrotaline-induced pulmonary artery hypertension via inhibiting NLRP3 inflammasome activation in rats. Int J Cardiol. 2015; 180: 134-41.
304. Tawa M, Furukawa T, Tongu H, Sugihara M, Taguwa S, Yamanaka M, Yano Y, Matsumori H, Kitada R, Sawano T, Tanaka R, Ohkita M, Matsumura Y. Stimulation of nitric oxide-sensitive soluble guanylate cyclase in monocrotaline-induced pulmonary hypertensive rats. Life Sci. 2018; 203: 203-209.
305. Thompson JS, Sheedy W, Morice AH. Effects of the neutral endopeptidase inhibitor, SCH 42495, on the cardiovascular remodelling secondary to chronic hypoxia in rats. Clin Sci (Lond). 1994; 87: 109-14.
306. Thompson JS, Sheedy W, Morice AH. Neutral endopeptidase (NEP) inhibition in rats with established pulmonary hypertension secondary to chronic hypoxia. Br J Pharmacol. 1994; 113: 1121-6.
307. Tian X, Vroom C, Ghofrani HA, Weissmann N, Bieniek E, Grimminger F, Seeger W, Schermuly RT, Pullamsetti SS. Phosphodiesterase 10A upregulation contributes to pulmonary vascular remodeling. PLoS One. 2011; 6: e18136.
308. Tofovic SP, Jones T, Petrusevska G. Dose-dependent therapeutic effects of 2-Methoxyestradiol on Monocrotaline-Induced pulmonary hypertension and vascular remodelling. Prilozi. 2010; 31: 279-95.
309. Tofovic SP, Jones TJ, Bilan VP, Jackson EK, Petrusevska G. Synergistic therapeutic effects of 2-methoxyestradiol with either sildenafil or bosentan on amelioration of monocrotaline-induced pulmonary hypertension and vascular remodeling. J Cardiovasc Pharmacol. 2010; 56: 475-83.
310. Tofovic SP, Salah EM, Mady HH, Jackson EK, Melhem MF. Estradiol metabolites attenuate monocrotaline-induced pulmonary hypertension in rats. J Cardiovasc Pharmacol. 2005; 46: 430-7.
311. Tofovic SP, Zhang X, Zhu H, Jackson EK, Rafikova O, Petrusevska G. 2-Ethoxyestradiol is antimitogenic and attenuates monocrotaline-induced pulmonary hypertension and vascular remodeling. Vascul Pharmacol. 2008; 48: 174-83.
312. Tual L, Morel OE, Favret F, Fouillit M, Guernier C, Buvry A, Germain L, Dhonneur G, Bernaudin JF, Richalet JP. Carvedilol inhibits right ventricular hypertrophy induced by chronic hypobaric hypoxia. Pflugers Arch. 2006; 452: 371-9.
313. Türck P, Lacerda DS, Carraro CC, de Lima-Seolin BG, Teixeira RB, Poletto Bonetto JH, Colombo R, Schenkel PC, Belló-Klein A, da Rosa Araujo AS. Trapidil improves hemodynamic, echocardiographic and redox state parameters of right ventricle in monocrotaline-induced pulmonary arterial hypertension model. Biomed Pharmacother. 2018; 103: 182-190.
314. Umar S, Iorga A, Matori H, Nadadur RD, Li J, Maltese F, van der Laarse A, Eghbali M. Estrogen rescues preexisting severe pulmonary hypertension in rats. Am J Respir Crit Care Med. 2011; 184: 715-23.
315. Uzun O, Balbay O, Comunoglu NU, Yavuz O, Nihat Annakkaya A, Güler S, Silan C, Erbaş M, Arbak P. Hypobaric-hypoxia-induced pulmonary damage in rats ameliorated by antioxidant erdosteine.Acta Histochem. 2006; 108: 59-68.
316. Vignozzi L, Morelli A, Cellai I, Filippi S, Comeglio P, Sarchielli E, Maneschi E, Vannelli GB, Adorini L, Maggi M. Cardiopulmonary protective effects of the selective FXR agonist obeticholic acid in the rat model of monocrotaline-induced pulmonary hypertension. J Steroid Biochem Mol Biol. 2017; 165(Pt B): 277-292.
317. Villegas LR, Kluck D, Field C, Oberley-Deegan RE, Woods C, Yeager ME, El Kasmi KC, Savani RC, Bowler RP, Nozik-Grayck E. Superoxide dismutase mimetic, MnTE-2-PyP, attenuates chronic hypoxia-induced pulmonary hypertension, pulmonary vascular remodeling, and activation of the NALP3 inflammasome. Antioxid Redox Signal. 2013; 18: 1753-64.
318. Wang AP, Li XH, Yang YM, Li WQ, Zhang W, Hu CP, Zhang Z, Li YJ. A Critical Role of the mTOR/eIF2α. Pathway in Hypoxia-Induced Pulmonary Hypertension.PLoS One. 2015; 10: e0130806.
319. Wang HL, Zhang XH, Jin X, Xing J, Zhang DR. Tetrandrine inhibited chronic "inflammatory" pulmonary hypertension in rats. Zhongguo Yao Li Xue Bao. 1997; 18: 401-4.
320. Wang HM, Liu WZ, Tang FT, Sui HJ, Zhan XJ, Wang HX. Cystamine slows but not inverses the progression of monocrotaline-induced pulmonary arterial hypertension in rats. Can J Physiol Pharmacol. 2018; 96: 783-789.
321. Wang HM, Wang Y, Liu M, Bai Y, Zhang XH, Sun YX, Wang HL. Fluoxetine inhibits monocrotaline-induced pulmonary arterial remodeling involved in inhibition of RhoA-Rho kinase and Akt signalling pathways in rats. Can J Physiol Pharmacol. 2012; 90: 1506-15.
322. Wang J, Jiang Q, Wan L, Yang K, Zhang Y, Chen Y, Wang E, Lai N, Zhao L, Jiang H, Sun Y, Zhong N, Ran P, Lu W. Sodium tanshinone IIA sulfonate inhibits canonical transient receptor potential expression in pulmonary arterial smooth muscle from pulmonary hypertensive rats. Am J Respir Cell Mol Biol. 2013; 48: 125-34.
323. Wang L, Zheng Q, Yuan Y, Li Y, Gong X. Effects of 17β-estradiol and 2-methoxyestradiol on the oxidative stress-hypoxia inducible factor-1 pathway in hypoxic pulmonary hypertensive rats. Exp Ther Med. 2017; 13: 2537-2543.
324. Wang LX, Sun Y, Chen C, Huang XY, Lin Q, Qian GQ, Dong W, Chen YF. Effects and mechanism of oridonin on pulmonary hypertension induced by chronic hypoxia-hypercapnia in rats. Chin Med J (Engl). 2009; 122: 1380-7.
325. Wang SH, Liu S, Yang JH, Wang ZZ, Li J, Hou WW. Protective effects of beraprost plus simvastatin on monocrotaline-induced pulmonary arterial hypertension in rats. Zhonghua Yi Xue Za Zhi. 2013; 93: 1691-5.
326. Wang W, Wang YL, Chen XY, Li YT, Hao W, Jin YP, Han B. Dexamethasone attenuates development of monocrotaline-induced pulmonary arterial hypertension. Mol Biol Rep. 2011; 38: 3277-84.
327. Wang X, Cai X, Wang W, Jin Y, Chen M, Huang X, Zhu X, Wang L. Effect of asiaticoside on endothelial cells in hypoxia‑induced pulmonary hypertension. Mol Med Rep. 2018; 17: 2893-2900.
328. Wang X, Yang Y, Yang D, Tong G, Lv S, Lin X, Chen C, Dong W. Tetrandrine prevents monocrotaline-induced pulmonary arterial hypertension in rats through regulation of the protein expression of inducible nitric oxide synthase and cyclic guanosine monophosphate-dependent protein kinase type 1. J Vasc Surg. 2015; S0741-5214(15)01966-7.
329. Wang XB, Wang W, Zhu XC, Ye WJ, Cai H, Wu PL, Huang XY, Wang LX. The potential of asiaticoside for TGF-ß1/Smad signaling inhibition in prevention and progression of hypoxia-induced pulmonary hypertension. Life Sci. 2015; 137: 56-64.
330. Wang XF, Lu WX, Guo J, Li G, Zhang YJ. Protective effects of rosiglitazone intervention on monocrotaline-induced pulmonary arterial hypertension in rats and related inflammatory mechanisms. Zhonghua Yi Xue Za Zhi. 2012; 92: 2144-7.
331. Wang Y, Han DD, Wang HM, Liu M, Zhang XH, Wang HL. Downregulation of osteopontin is associated with fluoxetine amelioration of monocrotaline-induced pulmonary inflammation and vascular remodelling. Clin Exp Pharmacol Physiol. 2011; 38: 365-72.
332. Wang Y, Tian W, Xiu C, Yan M, Wang S, Mei Y. Urantide improves the structure and function of right ventricle as determined by echocardiography in monocrotaline-induced pulmonary hypertension rat model. Clin Rheumatol. 2019; 38: 29-35.
333. Wang Y, Zhang XH, Wang HL. Involvement of BMPR2 in the protective effect of fluoxetine against monocrotaline-induced endothelial apoptosis in rats. Can J Physiol Pharmacol. 2011; 89: 345-54.
334. Wang YD, Li YD, Ding XY, Wu XP, Li C, Guo DC, Shi YP, Lu XZ. 17β-estradiol preserves right ventricular function in rats with pulmonary arterial hypertension: an echocardiographic and histochemical study. Int J Cardiovasc Imaging. 2019; 35: 441-450.
335. Wang YX, Liu ML, Zhang B, Fu EQ, Li ZC. Fasudil alleviated hypoxia-induced pulmonary hypertension by stabilizing the expression of angiotensin-(1-7) in rats. Eur Rev Med Pharmacol Sci. 2016; 20: 3304-12.
336. Wei L, Liu T, Liu B, Wang XM, Zhao L, Zhou TF. Effect of triptolide on the expression of matrix metalloproteinases 2 and 9 in lungs of experimental pulmonary hypertension. Zhongguo Dang Dai Er Ke Za Zhi. 2007; 9: 479-83.
337. Weissmann N, Hackemack S, Dahal BK, Pullamsetti SS, Savai R, Mittal M, Fuchs B, Medebach T, Dumitrascu R, Eickels Mv, Ghofrani HA, Seeger W, Grimminger F, Schermuly RT. The soluble guanylate cyclase activator HMR1766 reverses hypoxia-induced experimental pulmonary hypertension in mice. Am J Physiol Lung Cell Mol Physiol. 2009; 297: L658-65.
338. Wilson DN, Schacht SE, Al-Nakkash L, Babu JR, Broderick TL. Resveratrol prevents pulmonary trunk remodeling but not right ventricular hypertrophy in monocrotaline-induced pulmonary hypertension. Pathophysiology. 2016; 23: 243-250.
339. Wisutthathum S, Demougeot C, Totoson P, Adthapanyawanich K, Ingkaninan K, Temkitthawon P, Chootip K. Eulophia macrobulbon extract relaxes rat isolated pulmonary artery and protects against monocrotaline-induced pulmonary arterial hypertension. Phytomedicine. 2018; 50: 157-165.
340. Wu F, Hao Y, Yang J, Yao W, Xu Y, Yan L, Niu Y, Sun T, Yu J, Zhou R. Protective effects of aloperine on monocrotaline-induced pulmonary hypertension in rats.Biomed Pharmacother. 2017; 89: 632-641.
341. Wu F, Yao W, Yang J, Zhang M, Xu Y, Hao Y, Yan L, Niu Y, Sun T, Yu J, Zhou R. Protective effects of aloperin on monocroline-induced pulmonary hypertension via regulation of Rho A/Rho kinsase pathway in rats. Biomed Pharmacother. 2017; 95: 1161-1168.
342. Wu JR, Kao LP, Wu BN, Dai ZK, Wang YY, Chai CY, Chen IJ. Buffered l-ascorbic acid, alone or bound to KMUP-1 or sildenafil, reduces vascular endothelium growth factor and restores endothelium nitric oxide synthase in hypoxic pulmonary artery. Kaohsiung J Med Sci. 2015; 31: 241-54.
343. Wu Q, Wang HY, Li J, Zhou P, Wang QL, Zhao L, Fan R, Wang YM, Xu XZ, Yi DH, Yu SQ, Pei JM. κ-opioid receptor stimulation improves endothelial function in hypoxic pulmonary hypertension. PLoS One. 2013; 8: e60850.
344. Wu Y, Adi D, Long M, Wang J, Liu F, Gai MT, Aierken A, Li MY, Li Q, Wu LQ, Ma YT, Hujiaaihemaiti M. 4-Phenylbutyric Acid Induces Protection against Pulmonary Arterial Hypertension in Rats. PLoS One. 2016; 11: e0157538.
345. Xia XQ, Cheng DY, Guan J, Chen XJ. Preventive effects of urapidil on hypoxic pulmonary hypertension in rats. Sichuan Da Xue Xue Bao Yi Xue Ban. 2004; 35: 71-3.
346. Xie L, Lin P, Xie H, Xu C. Effects of atorvastatin and losartan on monocrotaline-induced pulmonary artery remodeling in rats. Clin Exp Hypertens. 2010; 32: 547-54.
347. Xie W, Wang H, Wang H, Hu G. Effects of iptakalim hydrochloride, a novel KATP channel opener, on pulmonary vascular remodeling in hypoxic rats. Life Sci. 2004; 75: 2065-76.
348. Xie X, Wang G, Zhang D, Zhang Y, Zhu Y, Li F, Li S, Li M. Activation of peroxisome proliferator-activated receptor ? ameliorates monocrotaline-induced pulmonary arterial hypertension in rats. Biomed Rep. 2015; 3: 537-542.
349. Xin WX, Li QL, Fang L, Zhong LK, Zheng XW, Huang P. Preventive Effect and Mechanism of Ethyl Acetate Extract of Sceptridium ternatum in Monocrotaline-Induced Pulmonary Arterial Hypertension. Chin J Integr Med. 2018. [Epub ahead of print]
350. Xin Y, Lv JQ, Wang YZ, Zhang J, Zhang X. Effect of all-trans retinoic acids (ATRA) on the expression of α-smooth muscle actin (α-SMA) in the lung tissues of rats with pulmonary arterial hypertension (PAH). Genet Mol Res. 2015; 14: 14308-13.
351. Xu DQ, Luo Y, Liu Y, Wang J, Zhang B, Xu M, Wang YX, Dong HY, Dong MQ, Zhao PT, Niu W, Liu ML, Gao YQ, Li ZC. Beta-estradiol attenuates hypoxic pulmonary hypertension by stabilizing the expression of p27kip1 in rats. Respir Res. 2010; 11: 182.
352. Xu Y, Gu Q, Qu C. Capsaicin pretreatment reversed pulmonary arterial hypertension by alleviating inflammation via p38MAPK pathway. Exp Lung Res. 2017; 43: 8-18.
353. Yamada Y, Maruyama J, Zhang E, Okada A, Yokochi A, Sawada H, Mitani Y, Hayashi T, Suzuki K, Maruyama K. Effect of thrombomodulin on the development of monocrotaline-induced pulmonary hypertension. J Anesth. 2014; 28: 26-33.
354. Yan J, Chen R, Liu P, Gu Y. Docosahexaenoic acid inhibits development of hypoxic pulmonary hypertension: in vitro and in vivo studies. Int J Cardiol. 2013; 168: 4111-6.
355. Yang DL, Zhang HG, Xu YL, Gao YH, Yang XJ, Hao XQ, Li XH. Resveratrol inhibits right ventricular hypertrophy induced by monocrotaline in rats. Clin Exp Pharmacol Physiol. 2010; 37: 150-5.
356. Yang H, Xu Y, Zhang Z. The effect of ginkgo biloba on hypoxic pulmonary hypertension and the role of protein kinase C. Zhonghua Jie He He Hu Xi Za Zhi. 2000; 23: 602-5.
357. Yang JM, Zhou R, Zhang M, Tan HR, Yu JQ. Betaine Attenuates Monocrotaline-Induced Pulmonary Arterial Hypertension in Rats via Inhibiting Inflammatory Response. Molecules. 2018; 23. pii: E1274.
358. Yang L, Zheng BX, Cheng DY, Su QL, Fan LL, Yang YJ, Mu M, Chen WB. The effect of breviscapine on the pulmonary arterial pressure and the expression of Rho-kinase in pulmonary arterioles of hypoxic rats. Zhonghua Jie He He Hu Xi Za Zhi. 2008; 31: 826-30.
359. Yang PS, Kim DH, Lee YJ, Lee SE, Kang WJ, Chang HJ, Shin JS. Glycyrrhizin, inhibitor of high mobility group box-1, attenuates monocrotaline-induced pulmonary hypertension and vascular remodeling in rats. Respir Res. 2014; 15: 148.
360. Yavuz T, Uzun O, Macit A, Comunoglu C, Yavuz O, Silan C, Yuksel H, Yildirim HA. Pyrrolidine dithiocarbamate attenuates the development of monocrotaline-induced pulmonary arterial hypertension. Pathol Res Pract. 2013; 209: 302-8.
361. Ye JX, Wang SS, Ge M, Wang DJ. Suppression of endothelial PGC-1α is associated with hypoxia-induced endothelial dysfunction and provides a new therapeutic target in pulmonary arterial hypertension. Am J Physiol Lung Cell Mol Physiol. 2016; 310: L1233-42.
362. Yin Y, Wu X, Yang Z, Zhao J, Wang X, Zhang Q, Yuan M, Xie L, Liu H, He Q. The potential efficacy of R8-modified paclitaxel-loaded liposomes on pulmonary arterial hypertension. Pharm Res. 2013; 30: 2050-62.
363. Yu J, Feng HS, Chen BY, Qu P, Liu LB, Chen JK, Tie R, Huang XJ, Zhao YF, Zhu XX, Zhu MZ. Protective effects of vasonatrin peptide against hypobaric hypoxia-induced pulmonary hypertension in rats. Clin Exp Pharmacol Physiol. 2010; 37: 69-74.
364. Yu L, Fan ZY, Xie L, Li CY, Qin CY, Liu LJ, Liu HM. Mechanisms for reversal of pulmonary hypertension by rapamycin in rats. Zhongguo Dang Dai Er Ke Za Zhi. 2015; 17: 731-5.
365. Yu L, Tu Y, Jia X, Fang K, Liu L, Wan L, Xiang C, Wang Y, Sun X, Liu T, Yu D, Cao W, Song Y, Fan Y. Resveratrol Protects Against Pulmonary Arterial Hypertension in Rats via Activation of Silent Information Regulator 1. Cell Physiol Biochem. 2017; 42: 55-67.
366. Yu W, Ji W, Mi L, Lin C. Mechanisms of N‑acetylcysteine in reducing monocrotaline‑induced pulmonary hypertension in rats: Inhibiting the expression of Nox1 in pulmonary vascular smooth muscle cells. Mol Med Rep. 2017; 16: 6148-6155.
367. Yu W, Song X, Lin C, Ji W. Interventions and mechanisms of N-acetylcysteine on monocrotaline-induced pulmonary arterial hypertension. Exp Ther Med. 2018; 15: 5503-5509.
368. Yuan LB, Hua CY, Gao S, Yin YL, Dai M, Meng HY, Li PP, Yang ZX, Hu QH. Astragalus Polysaccharides Attenuate Monocrotaline-Induced Pulmonary Arterial Hypertension in Rats. Am J Chin Med. 2017; 45: 773-789.
369. Yuyama H, Fujimori A, Sanagi M, Koakutsu A, Noguchi Y, Sudoh K, Sasamata M, Miyata K. A novel and selective endothelin ET(A) receptor antagonist YM598 prevents the development of chronic hypoxia-induced pulmonary hypertension in rats. Vascul Pharmacol. 2005; 43: 40-6.
370. Zambelli V, Santaniello A, Fumagalli F, Masson S, Scorza R, Beretta L, Latini R. Efficacy of aminaftone in a rat model of monocrotaline-induced pulmonary hypertension. Eur J Pharmacol. 2011; 667: 287-91.
371. Zapata-Sudo G, Pontes LB, da Silva JS, Lima LM, Nunes IK, Barreiro EJ, Sudo RT. Benzenesulfonamide attenuates monocrotaline-induced pulmonary arterial hypertension in a rat model. Eur J Pharmacol. 2012; 690: 176-82.
372. Zeng Z, Huang H, Li X. The effect and mechanism of felodipine on monocrotaline induced pulmonary hypertension in rats. Zhonghua Jie He He Hu Xi Za Zhi. 2002; 25: 461-4.
373. Zhai FG, Zhang XH, Wang HL. Fluoxetine protects against monocrotaline-induced pulmonary arterial hypertension: potential roles of induction of apoptosis and upregulation of Kv1.5 channels in rats. Clin Exp Pharmacol Physiol. 2009; 36: 850-6.
374. Zhang B, Niu W, Xu D, Li Y, Liu M, Wang Y, Luo Y, Zhao P, Liu Y, Dong M, Sun R, Dong H, Li Z. Oxymatrine prevents hypoxia- and monocrotaline-induced pulmonary hypertension in rats. Free Radic Biol Med. 2014; 69: 198-207.
375. Zhang D, Wang G, Han D, Zhang Y, Xu J, Lu J, Li S, Xie X, Liu L, Dong L, Li M.Activation of PPAR-γ ameliorates pulmonary arterial hypertension via inducing heme oxygenase-1 and p21(WAF1): an in vivo study in rats. Life Sci. 2014; 98: 39-43.
376. Zhang E, Jiang B, Yokochi A, Maruyama J, Mitani Y, Ma N, Maruyama K. Effect of all-trans-retinoic acid on the development of chronic hypoxia-induced pulmonary hypertension.Circ J. 2010; 74: 1696-703.
377. Zhang E, Maruyama J, Yokochi A, Mitani Y, Sawada H, Nishikawa M, Ma N, Maruyama K. Sarpogrelate hydrochloride, a serotonin 5HT2A receptor antagonist, ameliorates the development of chronic hypoxic pulmonary hypertension in rats. J Anesth. 2015; 29: 715-23.
378. Zhang L, Li J, Shi Q, Fan R, Kaye AJ, Wang Y, Sun X, Rivera FB, Kaye AD, Pei J. Role of κ-opioid receptor in hypoxic pulmonary artery hypertension and its underlying mechanism. Am J Ther. 2013; 20: 329-36.
379. Zhang L, Pu Z, Wang J, Zhang Z, Hu D, Wang J. Baicalin inhibits hypoxia-induced pulmonary artery smooth muscle cell proliferation via the AKT/HIF-1α/p27-associated pathway. Int J Mol Sci. 2014; 15: 8153-68.
380. Zhang LL, Lu J, Li MT, Wang Q, Zeng XF. Preventive and remedial application of etanercept attenuate monocrotaline-induced pulmonary arterial hypertension. Int J Rheum Dis. 2016; 19: 192-8.
381. Zhang N, Dong M, Luo Y, Zhao F, Li Y. Danshensu prevents hypoxic pulmonary hypertension in rats by inhibiting the proliferation of pulmonary artery smooth muscle cells via TGF-β-smad3-associated pathway. Eur J Pharmacol. 2018; 820:1-7.
382. Zhang WH, Liu CP, Zhang YJ, Ji YQ, Lu WX, Zeng Q. Additive effect of tadalafil and simvastatin on monocrotaline-induced pulmonary hypertension rats. Scand Cardiovasc J. 2012; 46: 374-80.
383. Zhang WH, Lu WX, Zhang YJ, Ji YQ, Liu CP, Li G. Simvastatin prevents the development of pulmonary hypertension in the rats through reduction of inflammation. Zhonghua Yi Xue Za Zhi. 2009; 89: 855-9.
384. Zhang X, Chen J, Xu P, Tian X. Protective effects of astragaloside IV against hypoxic pulmonary hypertension. Medchemcomm. 2018; 9: 1715-1721.
385. Zhang Y, Cui Y, Deng W, Wang H, Qin W, Huang C, Li C, Zhang J, Guo Y, Wu D, Guo H. Isoquercitrin protects against pulmonary hypertension via inhibiting PASMCs proliferation. Clin Exp Pharmacol Physiol. 2017; 44: 362-370.
386. Zhang Y, Dai L, Wu S, Chen P, Zhao S. Atorvastatin attenuates involvement of RhoA/Rho-kinase pathway and NF-κB activation in hypoxic pulmonary hypertensive rats. Chin Med J (Engl) 2014; 127: 869-72.
387. Zhang Y, Wang D, Zhu T, Li C. Genistein attenuates monocrotaline-induced pulmonary arterial hypertension in rats by up-regulating heme oxygenase-1 expression. Nan Fang Yi Ke Da Xue Xue Bao. 2012; 32: 151-4.
388. Zhang Y, Xie X, Zhu Y, Liu L, Feng W, Pan Y, Zhai C, Ke R, Li S, Song Y, Fan Y, Fan F, Wang X, Li F, Li M. Inhibition of Notch3 prevents monocrotaline-induced pulmonary arterial hypertension. Exp Lung Res. 2015; 41: 435-43.
389. Zhang YF, Zheng Y. The effects of mycophenolate mofetil on cytokines and their receptors in pulmonary arterial hypertension in rats. Scand J Rheumatol. 2015; 44: 412-5.
390. Zhang Z, Zhang L, Sun C, Kong F, Wang J, Xin Q, Jiang W, Li K, Chen O, Luan Y. Baicalin attenuates monocrotaline-induced pulmonary hypertension through bone morphogenetic protein signaling pathway. Oncotarget. 2017; 8: 63430-63441.
391. Zhao J, Yang M, Wu X, Yang Z, Jia P, Sun Y, Li G, Xie L, Liu B, Liu H. Effects of paclitaxel intervention on pulmonary vascular remodeling in rats with pulmonary hypertension. Exp Ther Med. 2019; 17: 1163-1170.
392. Zhao L, al-Tubuly R, Sebkhi A, Owji AA, Nunez DJ, Wilkins MR. Angiotensin II receptor expression and inhibition in the chronically hypoxic rat lung. Br J Pharmacol. 1996; 119: 1217-22.
393. Zhao L, Sebkhi A, Ali O, Wojciak-Stothard B, Mamanova L, Yang Q, Wharton J, Wilkins MR. Simvastatin and sildenafil combine to attenuate pulmonary hypertension. Eur Respir J. 2009; 34: 948-57.
394. Zhao S, Zheng MX, Chen HE, Wu CY, Wang WT. Effect of panax notoginseng saponins injection on the p38MAPK pathway in lung tissue in a rat model of hypoxic pulmonary hypertension. Chin J Integr Med. 2015; 21: 147-51.
395. Zheng L, Liu M, Wei M, Liu Y, Dong M, Luo Y, Zhao P, Dong H, Niu W, Yan Z, Li Z. Tanshinone IIA attenuates hypoxic pulmonary hypertension via modulating KV currents. Respir Physiol Neurobiol. 2015; 205: 120-8.
396. Zhong X, Liang G, He Z. Laboratory study on protective and therapeutic effects of pinacidil on pulmonary vascular remodeling in rats with hypoxia-induced pulmonary hypertension. Zhonghua Jie He He Hu Xi Za Zhi. 2000; 23: 727-9.
397. Zhou KR, Lai YL. Capsaicin pretreatment attenuates monocrotaline-induced ventilatory dysfunction and pulmonary hypertension. J Appl Physiol (1985). 1993; 75: 2781-8.
398. Zhou Q, Wang D, Liu Y, Yang X, Lucas R, Fischer B. Solnatide Demonstrates Profound Therapeutic Activity in a Rat Model of Pulmonary Edema Induced by Acute Hypobaric Hypoxia and Exercise. Chest. 2017; 151: 658-667.
399. Zhou S, Li MT, Jia YY, Liu JJ, Wang Q, Tian Z, Liu YT, Chen HZ, Liu DP, Zeng XF. Regulation of Cell Cycle Regulators by SIRT1 Contributes to Resveratrol-Mediated Prevention of Pulmonary Arterial Hypertension. Biomed Res Int. 2015; 762349.
400. Zhu N, Zhao X, Xiang Y, Ye S, Huang J, Hu W, Lv L, Zeng C. Thymoquinone attenuates monocrotaline-induced pulmonary artery hypertension via inhibiting pulmonary arterial remodeling in rats. Int J Cardiol. 2016; 221: 587-96.
401. Zhu R, Bi L, Kong H, Xie W, Hong Y, Wang H. Ruscogenin exerts beneficial effects on monocrotaline-induced pulmonary hypertension by inhibiting NF-κB expression. Int J Clin Exp Pathol. 2015; 8: 12169-76.
402. Zhu R, Bi LQ, Wu SL, Li L, Kong H, Xie WP, Wang H, Meng ZL. Iptakalim attenuates hypoxia-induced pulmonary arterial hypertension in rats by endothelial function protection. Mol Med Rep. 2015; 12: 2945-52.
403. Zhu S, Wang J, Wang X, Zhao J. Protection against monocrotaline-induced pulmonary arterial hypertension and caveolin-1 downregulation by fluvastatin in rats. Mol Med Rep. 2018; 17: 3944-3950.
404. Zhu SP, Mao ZF, Huang J, Wang JY. Continuous fluoxetine administration prevents recurrence of pulmonary arterial hypertension and prolongs survival in rats. Clin Exp Pharmacol Physiol. 2009; 36: e1-5.
405. Zhu TT, Zhang WF, Luo P, He F, Ge XY, Zhang Z, Hu CP. Epigallocatechin-3-gallate ameliorates hypoxia-induced pulmonary vascular remodeling by promoting mitofusin-2-mediated mitochondrial fusion. Eur J Pharmacol. 2017; 809: 42-51.
406. Zhu Y, Wu Y, Shi W, Wang J, Yan X, Wang Q, Liu Y, Yang L, Gao L, Li M. Inhibition of ubiquitin proteasome function prevents monocrotaline-induced pulmonary arterial remodeling. Life Sci. 2017; 173: 36-42.
407. Zopf DA, das Neves LA, Nikula KJ, Huang J, Senese PB, Gralinski MR. C-122, a novel antagonist of serotonin receptor 5-HT2B, prevents monocrotaline-induced pulmonary arterial hypertension in rats. Eur J Pharmacol. 2011; 670: 195-203.
408. Zuo X, Zong F, Wang H, Wang Q, Xie W, Wang H. Iptakalim, a novel ATP-sensitive potassium channel opener, inhibits pulmonary arterial smooth muscle cell proliferation by downregulation of PKC-α. J Biomed Res. 2011; 25: 392-401.
409. Zuo XR, Wang Q, Cao Q, Yu YZ, Wang H, Bi LQ, Xie WP, Wang H. Nicorandil prevents right ventricular remodeling by inhibiting apoptosis and lowering pressure overload in rats with pulmonary arterial hypertension. PLoS One. 2012; 7: e44485.
